# Spatio-temporal regulation of recombinase expression enables efficient autoexcision of selectable marker genes in soybean and maize

**DOI:** 10.1186/s12870-025-07215-0

**Published:** 2025-09-24

**Authors:** Brent O’Brien, Anagha Sant, Lisa Kanizay, Stanislaw Flasinski, Olivia Haragutchi, Xudong Ye, Anthony Paisley, Matthew S. Marengo

**Affiliations:** Plant Biotechnology, Bayer Crop Science, Chesterfield, United States

**Keywords:** Autoexcision, Cre, Gene expression, Selectable marker, Zea mays, Glycine max

## Abstract

**Background:**

Most plant transformation protocols include the use of a selectable marker gene to enable efficient selection of transformed cells for transgenic regeneration. While the marker gene facilitates the transformation process, it is not needed once transgenic plants have been identified and is removed during commercial development. Autoexcision is a marker removal process, in which a Cre-lox system is used to remove both the selectable marker gene and the Cre gene itself in the T0 generation. One challenge for autoexcision is achieving efficient marker removal while preserving the recovery rate for transgene-positive transformation events. Predictable and robust control of Cre gene expression is essential to maintaining this balance.

**Results:**

In this report, we demonstrate the utility of promoters and other expression elements that were selected based on the RNA-seq-derived, tissue-specific expression patterns of their associated endogenous genes. These elements were selected for their potential to drive Cre in a specific spatio-temporal manner. Functional elements expected to drive Cre primarily in floral meristem, gametes, and/or early embryo enabled efficient autoexcision and recovery of transgenic transformants. Additionally, we confirmed the expected expression patterns of successful elements via GUS staining.

**Conclusions:**

Overall, these elements enable an efficient approach for selectable marker gene removal during commercial development.

**Supplementary Information:**

The online version contains supplementary material available at 10.1186/s12870-025-07215-0.

## Background

Transgenic plants are typically transformed with a selectable marker gene (referred to as a “marker” in this report) along with the gene of interest (GOI). The marker allows for selection of the transformed plants. Markers may be removed during commercial development to minimize redundancy in transgene stacking or possible regulatory concerns over public acceptance [1, 2]. Current agricultural biotechnology approaches may include simultaneous use of transgenes and CRISPR/Cas editing “tools” [3]. Following a similar rationale to marker removal, editing tools may also be removed during commercial development.

A variety of approaches have been used to produce marker-free transgenic events, including transformations without markers and transformations in which the marker is on a separate transfer DNA (T-DNA), which can then be segregated out of the final product [4–7]. Alternatively, markers can be removed by recombinases, such as the Cre/lox system from the bacteriophage P1 [8]. The Cre enzyme can be used to excise DNA sequences, including markers and editing tools, when flanked by loxP recognition sites [9]. Constitutive Cre activity can be introduced by a sexual cross [10, 11]. This process, however, requires an extra generation for product development.

**Fig. 1:**
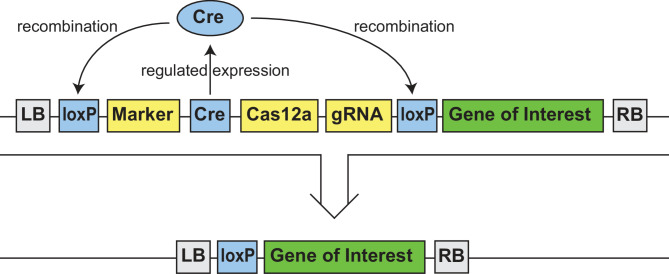
T-DNA schematic and overview of the autoexcision process. DNA sequences are shown as boxes, and proteins are shown as circles. LB and RB are the left and right border sequences of the T-DNA. The autoexcised portions of the T-DNA in this study ranged from 11.6 to 14.7 kbp

Autoexcision is a process of marker removal in which the marker and Cre gene are both located on a portion of the T-DNA that is flanked by loxP sites. Cre activity will thus excise both the marker and the Cre gene itself (Fig. 1). Broad expression of Cre will result in early marker excision and a decrease in transformed plants that survive selection. To avoid this, developmentally regulated and inducible promoters for Cre expression have been used [12–26]. Predictable and robust control of Cre gene expression is critical for efficient autoexcision without reduction in transformation efficiency. Here, we define autoexcision efficiency as the proportion of progeny plants that are homozygous for the GOI and lack any detectable marker.

In this report, we describe the autoexcision efficiency enabled by driving Cre with novel promoters, 5’ untranslated regions (UTRs), introns, and 3’ UTRs (henceforth termed expression elements). We selected expression elements based on RNA-seq patterns for associated endogenous genes. Expression elements from a given gene were tested together as a cassette, which we define as an operably linked sequence consisting of the expression elements and coding sequence for a transgene. We tested the autoexcision efficiency of different Cre cassettes for soybeans and maize.

## Methods

### Curation of novel expression elements

RNA-seq data from public and proprietary sources was visualized and analyzed using Tibco Spotfire (Supplementary Table S1). Expression elements were curated from candidate reproductive genes as follows: Promoters and 5’ UTRs: 2 kbp upstream of the coding region start codon; 3’ UTRs: 500 bp after the stop codon. In cases where there were neighboring genes within 2 kbp of the start codon of a candidate gene, the promoter was truncated so as not to overlap with neighboring genes. Sequences were mined from both public and elite soybean and maize genotypes.

### Plant transformation and cultivation

T-DNA vectors were transformed into *Agrobacterium tumefaciens* and introduced into soybean (*Glycine max)* or maize (*Zea mays)* by *Agrobacterium*-mediated transformation [27–29]. The transformation efficiency for each test construct was normalized to the transformation efficiency of a non-autoexcision control construct from the same time period. These non-autoexcision control constructs included a marker flanked by loxP sites but did not include a Cre cassette. Transformed plants were assayed for T-DNA insertion copy number by DNA TaqMan assays (see below). Progeny soybean plants were grown in Premier Tech PRO-MIX BX medium in greenhouses with supplemental lighting and fertigation. Progeny maize plants were grown in greenhouses in Berger BM2 mix with supplemental lighting and fertigation.

### Detection of marker, Cre, and GOI DNA

DNA detection was performed through qualitative or quantitative TaqMan as previously described [30]. Progeny plants were counted as homozygous marker-free if they had the following TaqMan copy calls: GOI: homozygous (2); marker: 0; Cre: absent or 0. Progeny plants were counted as hemizygous marker-free they had the following TaqMan copy calls: GOI: hemizygous (1); marker: 0; Cre: absent or 0.

### GUS reporter gene characterization

Low copy (generally single copy) R0 plants transformed with GUS reporter constructs were selected for staining. Fresh tissue fragments or cross-sections were placed directly into a 24 well plate containing X-Gluc staining solution: 1 mg/mL X-Gluc (5-bromo-4-chloro-3-indolyl-b-glucuronide), 25 µM potassium ferricyanide, 2.5 µM potassium ferrocyanide, 0.05% Triton X-100 (v/v) in a 50 mM potassium phosphate buffer (pH 7.4). The staining solution volume was sufficient to completely cover the tissue or the cross-sections. The tissue was incubated at 37 °C for 5 h. Staining solution was then replaced by destaining solution (35% w/v ethanol and 50% glacial acetic acid w/v in water) in sufficient volume to completely cover the tissue or the cross-sections. Tissues were incubated at room temperature for a minimum of 18 h before the destaining solution was removed and replaced with 70% ethanol. Stained tissues were imaged with a stereoscopic dissecting microscope. Scale bars were added post-hoc, using the same magnification and resolution, and when necessary calibrated on the same tissue from the same developmental time point. They should be taken as approximate.

## Results

We tested autoexcision efficiency in the context of T-DNAs containing a marker, CRISPR/Cas editing tools, and a GOI (Fig. 1). Soybean test vectors were identical outside of Cre cassette changes. Maize test vectors included variations in the gRNA and GOI cassettes. These variations were small: less than 2% of the size of the autoexcised region in any given construct. They were also in non-neighboring cassettes: more than 6 kbp away from the Cre cassette. Any changes in autoexcision efficiency were therefore attributed to changes in the Cre cassette.

**Fig. 2:**
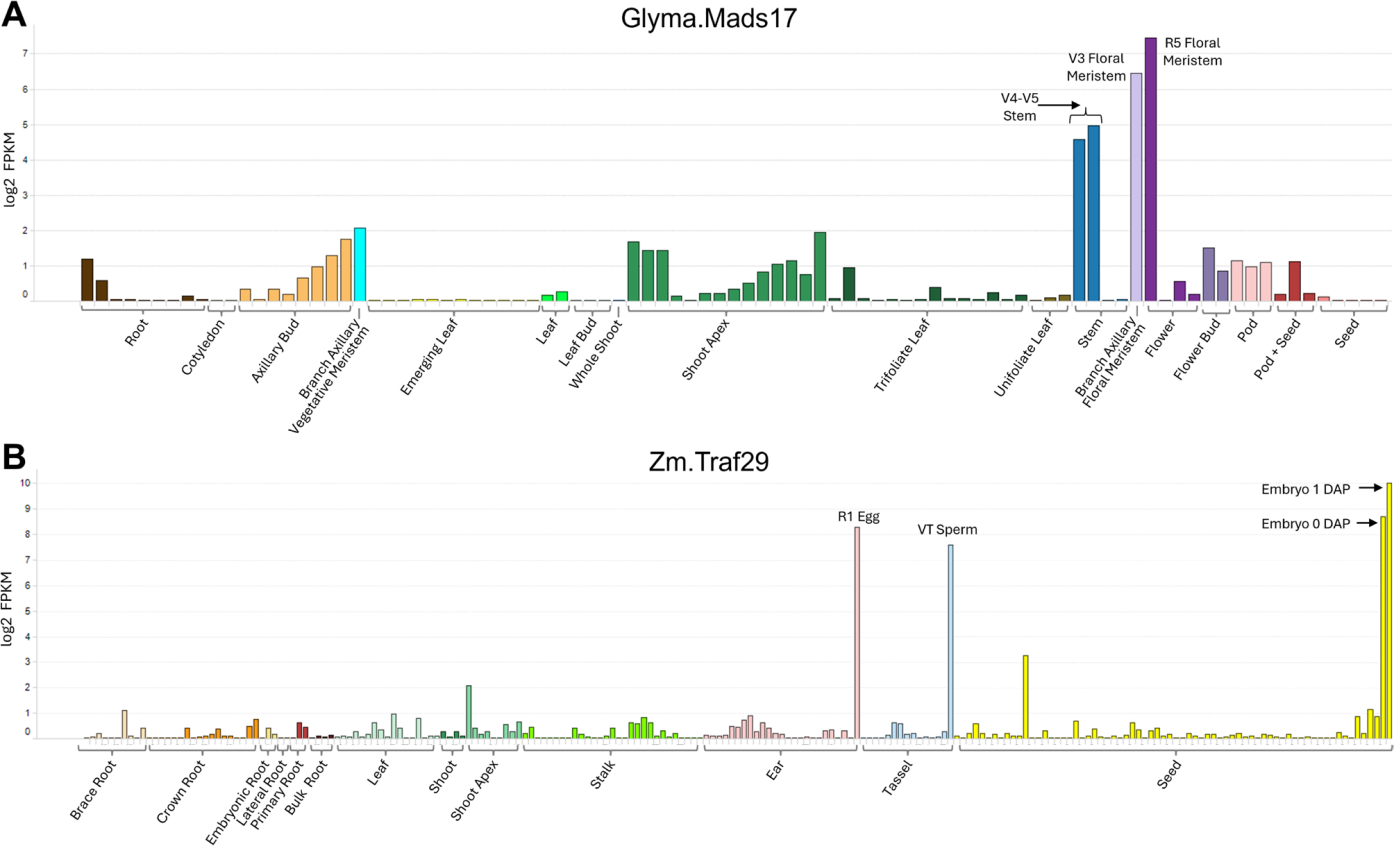
Representative expression profiles of soybean and maize reproductive genes. The bars along the X-axis are grouped by organ. Each bar represents transcriptomic data from a distinct tissue and/or developmental point (Supplementary Table S1). For clarity, only relevant reproductive tissues are labeled. The Y axis represents Fragments Per Kilobase of transcript per Million mapped reads (FPKM) in log_2_. **A** Expression profile for soybean Glyma.Mads17. **B** Expression profile for maize Zm.Traf29. Each bar represents transcriptomic data for one of 211 RNA-seq datasets from a distinct tissue and/or developmental point. Embryo samples are labeled with DAP: Days After Pollination. 0 DAP represents the day of pollination, specifically 12 h after pollination [31]

To ensure efficient autoexcision without reducing transformation efficiency, we focused on driving Cre expression specifically in reproductive meristem, late-stage reproductive tissues, or early embryo. Expression elements that enabled such profiles were mined by first identifying genes that were specifically and strongly expressed in target reproductive tissues (Fig. 2, Supplementary Fig. S2 and Table S3). We used transcriptomic data from maize and soybean to select genes that are primarily expressed in one or more of the following tissues: floral meristem, inflorescence meristem, gametes, or early embryo (Supplementary Table S3). In addition to mined elements from novel candidate genes, we also tested elements from previously reported reproductive genes. These genes included maize Cdc45 [26], soybean Rsp-1 [26], maize Traf29 [31], and *Setaria viridis* SPO11 [32].

We designed cassettes with these expression elements and the Cre coding sequence (Supplementary Table S4 and S5). In most cassettes, Cre was driven by the promoter and cognate 3’ UTR from the same source gene. However, in some instances, promoters were paired with heterologous 5’ UTR introns and 3’ UTRs.

**Fig. 3:**
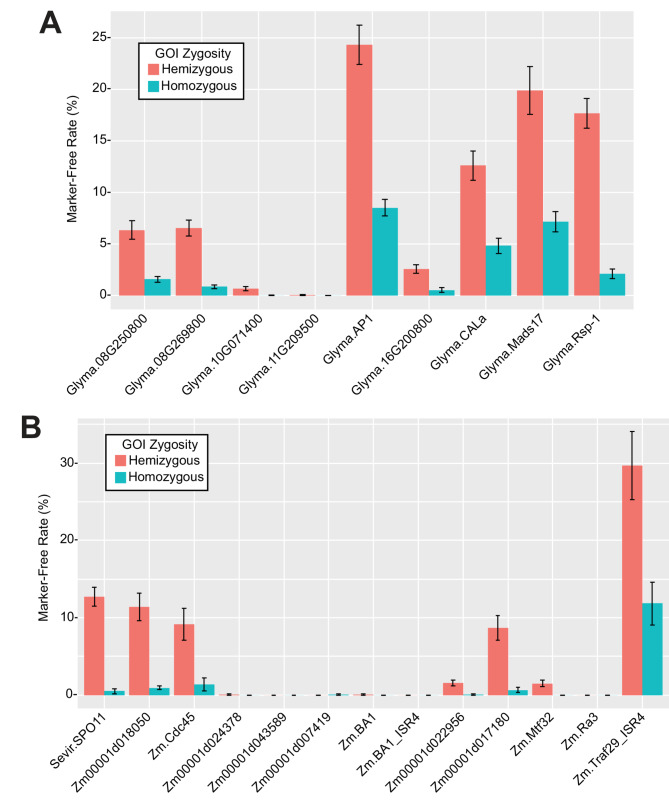
Autoexcision efficiency for Cre cassettes. Independent transgenic events were used as replicates, with at least 7 events tested for each Cre cassette. Error bars represent standard errors of the means. Cassettes labeled with the suffix “_ISR4” included a 1.2 kbp intergenic sequence at their 3’ end (Supplementary Table S4 and S5). Percentage rates are from the total tested progeny plants per event. The theoretical maximum rates for heterozygous and homozygous marker-free are 50% and 25%, respectively. **A** Results in soybeans. **B** Results in maize

Autoexcision was assayed alongside GOI zygosity in the T1 generation. The most useful progeny plants for transgenic product development are homozygous marker-free plants. Requiring homozygosity limits the maximum for this population to 25% of soybean or maize progeny. We transformed each construct once and analyzed mean autoexcision efficiencies using independent transgenic events as replicates (Fig. 3). A subset of successful constructs were independently transformed a second time to confirm efficacy. The results of all transformations were summarized (Table 1). The Cre cassette with Glyma.AP1 expression elements enabled the highest rate of homozygous marker-free soybean plants, 8.54% pooled across transgenic events. The Cre cassette with Zm.Traf29 expression elements and an intergenic sequence after its 3’ UTR enabled the highest rates of homozygous marker-free maize plants, 14.3 to 17.2% pooled across transgenic events. This intergenic sequence, named ISR4, was 1.2 kbp of randomized DNA with ATGs removed. Although the successful Zm.Traf29 Cre cassette included ISR4, adding ISR4 to the Zm.BA1 Cre cassette did not enable efficient autoexcision. We defined “hits” as cassettes that enabled over 5% (mean rates, Fig. 3) of progeny plants to be homozygous for the GOI and marker-free. Our hit rate in soybean was 2/9 (22%) and in maize was 1/13 (7.7%).

**Table 1:** Summary of autoexcision efficiency in test constructs. In this table, the data from all transgenic events per construct are pooled. The theoretical maximum rates for homozygous and hemizygous marker-free (MF) are 25% and 50%, respectively. *These promoters were also used to drive GUS in separate testing constructs (Fig. 5, Supplementary table S6)

Independent transformation	Crop	Cre promoter	Total # homo MF T1 plants	Total # hemi MF T1 plants	Total # T1 plants	% Homo MF​	% Hemi MF	Total # events analyzed	# Events with at least 1 T1 plant excised
1	Soybean	Glyma.10G071400	1	19	2677	0.04	0.71	25	10
		Glyma.11G209500	0	1	1736	0.00	0.06	24	1
		Glyma.16G200800	12	50	1739	0.69	2.88	24	19
		Glyma.CALa*	131	334	2540	5.16	13.2	24	23
		Glyma.Mads17*	201	558	2686	7.48	20.8	24	21
		Glyma.08G269800	26	198	2938	0.88	6.74	27	27
		Glyma.08G250800	45	179	2686	1.68	6.66	24	22
		Glyma.Rsp-1	59	454	2438	2.42	18.6	22	22
		Glyma.AP1*	239	687	2800	8.54	24.5	25	25
	Maize	Zm.BA1	1	3	1317	0.08	0.23	15	2
		Zm.Traf29*	154	345	1079	14.3	32.0	12	11
		Sevir.SPO11*	4	159	1236	0.32	12.9	15	15
		Zm00001d022956	1	18	963	0.10	1.87	10	7
		Zm00001d024378	0	1	639	0.00	0.16	7	1
		Zm00001d007419	1	0	1314	0.08	0.00	15	1
		Zm00001d017180	6	79	859	0.70	9.20	13	11
		Zm.Cdc45-1	19	129	1283	1.48	10.1	15	12
		Zm00001d043589	0	0	1750	0.00	0.00	16	0
		Zm.Ra3	0	0	952	0.00	0.00	11	0
		Zm.Mtf32	0	19	1310	0.00	1.45	15	8
		Zm00001d018050	13	157	1188	1.09	13.2	13	13
2	Soybean	Glyma.CALa*	175	399	2800	6.25	14.3	25	25
		Glyma.Mads17*	161	417	2799	5.75	14.9	25	25
	Maize	Zm.Traf29*	196	499	1143	17.2	43.7	14	14

**Fig. 4:**
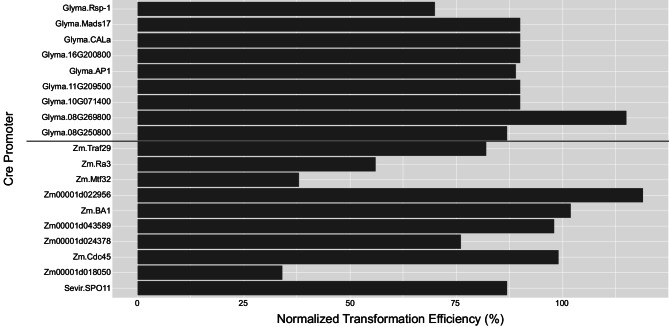
Novel Cre cassettes allow for efficient transformation. Transformation efficiency was measured as the rate of plant survival in selective media. The transformation efficiency for each test construct was normalized to the transformation efficiency of a non-autoexcision control construct from the same time period (as described in Methods). A gray line separates soybean results from maize results

We measured transformation efficiency as the rate of plant survival in selective media. The Zm.Ra3, Zm.Mtf32, and Zm00001d018050 cassettes decreased transformation efficiency below 60% of a non-autoexcision control vector transformed during the same time period (Fig. 4 and as described in Methods). Other cassettes, including the Glyma.AP1 and Zm.Traf29 cassettes, did not strongly decrease transformation efficiencies.

**Fig. 5:**
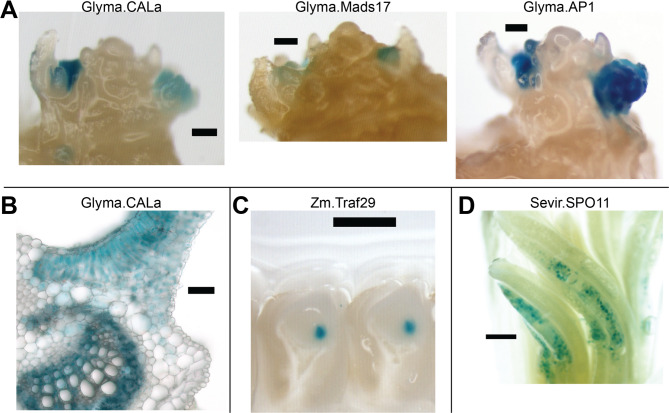
Expression elements that enabled efficient autoexcision also enabled GUS expression (blue staining) in germline tissues and/or their progenitors. **A** Whole mounts of dissected soybean shoot apices with apical meristem and axillary reproductive meristems. Expression elements that enabled efficient soybean autoexcision also enabled GUS expression in reproductive axillary meristems. Note the lack of expression in the apical meristem. Scale bars represent 100 μm. **B** Cross-section of R1 stage soybean leaf, including midrib. Glyma.CALa enabled GUS expression in phloem and mature mesophyll cells. Scale bar represents 10 μm. **C** Cross-section of developing maize seeds. Zm.Traf29, which enabled efficient maize autoexcision, also enabled GUS expression in early seed embryos. Scale bar represents 100 μm. **D** Whole mount of developing maize spikelets. Sevir.SPO11, which enabled maize autoexcision, also enabled GUS expression in developing pollen. Scale bar represents 100 μm

We further characterized the expression elements from the most efficient Cre cassettes with GUS reporter genes (Fig. 5). Tissues that stained positive for GUS expression included germline tissues and/or their progenitors, as predicted during expression element mining. As expected, Zm.Traf29 enabled strong GUS expression in early embryos, but tissue specificity was not absolute (Supplementary Table S6). In soybean, the characterized elements enabled expression in axillary meristem but also in other tissues, including phloem, nectaries, and mature mesophyll (Fig. 5B and Supplementary Table S6).

## Discussion

In this study, we used RNA-seq expression patterns to pick expression elements for Cre-mediated autoexcision. While we were able to find elements that drove efficient autoexcision, our hit rate in soybean was 22% and in maize was 7.7%. One possible explanation for these low hit rates could be that a subset of the test constructs are subject to epigenetic silencing of regions between lox sites [33], which in our case would include the Cre expression cassettes. In the future, we could increase our predictive power through analysis of gene expression regulation not reflected in RNA-seq data, such as epigenetic, translational, or post-translational regulation. These low hit rates could also be due to effects on expression from vector context. For example, the expression profile of Cre could be influenced by expression elements driving the GOI. To limit these effects, a subset of maize test constructs included a randomized 1.2 kbp intergenic sequence, ISR4. Future experiments could systematically test if other, diverse intergenic sequences and/or insulators [34, 35] facilitate predictable and robust autoexcision. These experiments could include investigations of intergenic structural features and their effects on autoexcision efficiency. Other potential explanations for the observed hit rates could be that, by taking only 2 kbp of sequence for the promoter, we are missing important *cis*-regulatory motifs. Alternatively, taking the promoter sequences out of their native context may prevent long-distance regulatory interactions that are necessary to achieve the intended expression profile, and thus efficient autoexcision.

We selected successful Cre cassettes and replaced Cre with a GUS reporter gene to look at the expression patterns driven by these elements. Ideal expression elements for autoexcision would drive expression only in germline cells late in plant development. When driving Cre, this would enable transformed material to survive selection, but prevent T1 progeny from inheriting markers, as we observed (Figs. 3 and 4; Table 1). When driving GUS, we might therefore expect these same elements to drive expression only in germline cells late in plant development. While we did observe GUS expression in the expected tissues, there was also limited expression in other tissues (Fig. 5, Supplementary Table S6). These observations could be because not all tissue types that were characterized with GUS were represented in the transcriptomic data from which the expression elements were mined. Other possible explanations include that we did not capture further distal regulatory motifs in the native promoter to exactly replicate the expression pattern in a transgenic context, or that by taking the promoter sequences out of their native context, we disrupted epigenetic regulation associated with the native gene.

There is nevertheless a discrepancy between an ideal GUS expression pattern only in germline cells late in plant development and our observed pattern, which also included limited expression in other tissues. This, however, can be explained by several fallible assumptions built into the ideal case. For example, the ideal case assumes that the GUS and Cre expression correlate perfectly when driven by the same expression elements. In reality, there may be differences in GUS and Cre coding sequence features (such as GC content) that affect tissue-specific expression levels. It is also possible that the potato LS1 intron, used in the coding regions for both Cre and GUS, contains motifs with the potential to interact with coding sequence or promoter features in ways that cause leaky or ectopic expression [36]. The ideal case assumes that any Cre expression in a somatic cell type will result in early and complete marker removal in that cell type and selection against the transformant. However, it may be that expression from these elements occurs later in development, after removal of the selection agent. Additionally, it’s possible that a low level of leaky Cre expression does not cause efficient marker removal in early somatic cell types, or that transformants can tolerate marker removal in a subset of somatic cell types and still survive selection.

## Conclusions

In this report, we used extensive maize and soybean transcriptomic data to identify reproductive-specific genes, from which expression elements were mined to drive Cre-enabled autoexcision. Functional elements driving Cre in floral meristem, gametes, and/or early embryo enabled efficient late stage autoexcision. This demonstrates that targeting Cre to reproductive tissues can provide effective, heritable autoexcision, without causing a strong decrease in transformation efficiency. This approach provides a rapid, economical, and efficient means for selectable marker removal from transgenic traits in these crops.

## Supplementary Information

Supplementary Material 1.

## Data Availability

Availability of data and materials: All data generated or analyzed during this study are included in this published article and its supplementary information files.

## Abbreviations

FPKM: Fragments per kilobase of transcript per million mapped reads
GOI: Gene of interest
LB: Left border
MF: Marker-free
RB: Right border
T-DNA: Transfer DNA
UTR: Untranslated region
X-Gluc: 5-bromo-4-chloro-3-indolyl-b-glucuronide
