# Supplementary material for: Spatio-temporal regulation of recombinase expression enables efficient autoexcision of selectable marker genes in soybean and maize
Source: BMC Plant Biol. 2025 Sep 24;25:1215. doi: 10.1186/s12870-025-07215-0 (PMC12461952; doi:10.1186/s12870-025-07215-0)
Supplement: Supplementary file 1 — Supplementary Material 1. [file 12870_2025_7215_MOESM1_ESM.docx]

**Spatio-temporal regulation of recombinase expression enables efficient autoexcision of selectable marker genes in soybean and maize**

**Supplementary Material:**

**Table S1** Examples of public data included in RNA-seq dataset. Dataset also included proprietary data (not shown).

| **Species** | **Organ** | **Tissue** | **DevStage** | **Note** | **Seq Reads** | **Reference** |
| --- | --- | --- | --- | --- | --- | --- |
| Maize | Shoot | bulk | V2 | stage is an estimate; 14 days old | SRP001359 | 1 |
| Maize | ShootApex | Meristem | V2 | meristem+P1;stage is an estimate; 14 days after planting | SRP001550 | 2 |
| Maize | ShootApex | Meristem L1 | V2 | stage is estimate; 14 days after planting | SRP001844 | 3 |
| Maize | ShootApex | Meristem L2 | V2 | stage is estimate; 14 days after planting | SRP001844 | 3 |
| Maize | ShootApex | Bulk | V6 |  | SRP013564 | 4 |
| Maize | Leaf | Bulk | V2 |  | SRP006463 | 5 |
| Maize | Leaf | Bulk | V2 |  | SRP006463 | 5 |
| Maize | Leaf | DevZone1 | V2 | basal zone, leaf 3 and 1 cm above leaf 3 ligule (sink) | SRA012297 | 6 |
| Maize | Leaf | DevZone2 | V2 | leaf 3 but 1 cm below leaf 2 ligule | SRA012297 | 6 |
| Maize | Leaf | DevZone3 | V2 | leaf 3 but 4 cm above leaf 2 ligule | SRA012297 | 6 |
| Maize | Leaf | DevZone4 | V2 | 1 cm below leaf tip (source) | SRA012297 | 6 |
| Maize | Leaf | DevZone1 | V2 | stage is estimate; 9 days after planting | SRP001315 | 3 |
| Maize | Leaf | DevZone4 | V2 | stage is estimate; 9 days after planting | SRP001315 | 3 |
| Maize | Leaf | Bulk | V2 | 7-8 mm leaf primordia | SRP013564 | 4 |
| Maize | Leaf | DevZone1 | R1 |  | SRP014792 | 7 |
| Maize | Leaf | DevZone1 | R1 |  | SRP014792 | 7 |
| Maize | Leaf | Mesophyll | V2 | from leaf tip | SRA012297 | 6 |
| Maize | Leaf | BundleSheath | V2 | from leaf tip | SRA012297 | 6 |
| Maize | PrimaryRoot | Bulk | VE | radicle | SRA049019 | 8 |
| Maize | Root | Bulk | V2 | stage is an estimate; 14 days old | SRP001359 | 1 |
| Maize | Ear | Bulk | V11 | 7-8 mm ear primordia | SRP013564 | 4 |
| Maize | Ear | WholeCob | V15 |  | SRP006463 | 5 |
| Maize | Ear | WholeCob | VT |  | SRP006463 | 5 |
| Maize | Ear | MatureOvule | R1 |  | SRP006463 | 5 |
| Maize | Ear | MatureSilk | R1 |  | SRP006463 | 5 |
| Maize | Ear | DevelopingOvary | VT | Ovaries during sporogenesis; stage is estimate | SRP005476 | 9 |
| Maize | Ear | MatureOvule | R1 | Maize ovary 1d after pollination | SRP014792 | 7 |
| Maize | Tassel | Bulk | V18 | pre-pollination | SRP006463 | 5 |
| Maize | Tassel | Bulk | VT | post-pollination | SRP006463 | 5 |
| Maize | Tassel | WholeAnther | VT |  | SRP006463 | 5 |
| Maize | Tassel | Pollen | VT |  | SRP006463 | 5 |
| Maize | Seed | Bulk | R1 |  | SRP006463 | 5 |
| Maize | Seed | Bulk | R2 |  | SRP006463 | 5 |
| Maize | Seed | Embryo | R4 |  | SRP006463 | 5 |
| Maize | Seed | Endosperm | R4 |  | SRP006463 | 5 |
| Maize | Seed | Aleurone | 8DAP |  | SRP049347 | 10 |
| Maize | Seed | BETL | 8DAP |  | SRP049347 | 10 |
| Maize | Seed | Endosperm | 8DAP |  | SRP049347 | 10 |
| Maize | Seed | Conducting | 8DAP |  | SRP049347 | 10 |
| Maize | Seed | Embryo | 8DAP |  | SRP049347 | 10 |
| Maize | Seed | EmbryoSurrounding | 8DAP |  | SRP049347 | 10 |
| Maize | Seed | Nucellus | 8DAP |  | SRP049347 | 10 |
| Maize | Seed | Chalazal | 8DAP |  | SRP049347 | 10 |
| Maize | Seed | Pericarp | 8DAP |  | SRP049347 | 10 |
| Maize | Seed | PedicelVascularRegion | 8DAP |  | SRP049347 | 10 |
| Maize | BraceRoot | Endodermis/Pericycle | V8 | stage is estimate - 60 d old | SRP062897 | 11 |
| Maize | BraceRoot | Endodermis/Pericycle | V8 | stage is estimate - 60 d old | SRP062897 | 11 |
| Maize | CrownRoot | Endodermis/Pericycle | V2 | stage is estimate - 14 d old | SRP062897 | 11 |
| Maize | CrownRoot | Endodermis/Pericycle | V2 | stage is estimate - 14 d old | SRP062897 | 11 |
| Maize | PrimaryRoot | Endodermis/Pericycle | V1 | stage is estimate - 7 d old | SRP062897 | 11 |
| Maize | PrimaryRoot | Endodermis/Pericycle | V1 | stage is estimate - 7 d old | SRP062897 | 11 |
| Maize | EmbryonicRoot | Endodermis/Pericycle | V2 | stage is estimate - 9 d old | SRP062897 | 11 |
| Maize | EmbryonicRoot | Endodermis/Pericycle | V2 | stage is estimate - 9 d old | SRP062897 | 11 |
| Maize | Ear | Egg | R1 |  | SRP105764 | 12 |
| Maize | Pollen | Sperm | VT |  | SRP105764 | 12 |
| Maize | Seed | Embryo | 0DAP | Day of pollination, specifically 12 hours after pollination | SRP105764 | 12 |
| Maize | Seed | Embryo | 1DAP |  | SRP105764 | 12 |
| Maize | Seed | bulk | 0DAP |  | SRP037559 | 13 |
| Maize | Seed | bulk | 2DAP |  | SRP037559 | 13 |
| Maize | Seed | bulk | 3DAP |  | SRP037559 | 13 |
| Maize | Seed | bulk | 4DAP |  | SRP037559 | 13 |
| Maize | Seed | Endosperm | 6DAP |  | SRP037559 | 13 |
| Maize | Seed | bulk | 6DAP |  | SRP037559 | 13 |
| Maize | Seed | Endosperm | 8DAP |  | SRP037559 | 13 |
| Maize | Seed | bulk | 8DAP |  | SRP037559 | 13 |
| Maize | Seed | embryo | 10DAP |  | SRP037559 | 13 |
| Maize | Seed | endosperm | 10DAP |  | SRP037559 | 13 |
| Maize | Seed | bulk | 10DAP |  | SRP037559 | 13 |
| Maize | Seed | embryo | 12DAP |  | SRP037559 | 13 |
| Maize | Seed | endosperm | 12DAP |  | SRP037559 | 13 |
| Maize | Seed | bulk | 12DAP |  | SRP037559 | 13 |
| Maize | Seed | embryo | 14DAP |  | SRP037559 | 13 |
| Maize | Seed | endosperm | 14DAP |  | SRP037559 | 13 |
| Maize | Seed | bulk | 14DAP |  | SRP037559 | 13 |
| Maize | Seed | embryo | 16DAP |  | SRP037559 | 13 |
| Maize | Seed | endosperm | 16DAP |  | SRP037559 | 13 |
| Maize | Seed | bulk | 16DAP |  | SRP037559 | 13 |
| Maize | Seed | embryo | 18DAP |  | SRP037559 | 13 |
| Maize | Seed | endosperm | 18DAP |  | SRP037559 | 13 |
| Maize | Seed | bulk | 18DAP |  | SRP037559 | 13 |
| Maize | Seed | embryo | 20DAP |  | SRP037559 | 13 |
| Maize | Seed | Endosperm | 20DAP |  | SRP037559 | 13 |
| Maize | Seed | embryo | 22DAP |  | SRP037559 | 13 |
| Maize | Seed | Endosperm | 22DAP |  | SRP037559 | 13 |
| Maize | Seed | bulk | 22DAP |  | SRP037559 | 13 |
| Maize | Seed | embryo | 24DAP |  | SRP037559 | 13 |
| Maize | Seed | Endosperm | 24DAP |  | SRP037559 | 13 |
| Maize | Seed | bulk | 24DAP |  | SRP037559 | 13 |
| Maize | Seed | embryo | 26DAP |  | SRP037559 | 13 |
| Maize | Seed | Endosperm | 26DAP |  | SRP037559 | 13 |
| Maize | Seed | bulk | 26DAP |  | SRP037559 | 13 |
| Maize | Seed | embryo | 28DAP |  | SRP037559 | 13 |
| Maize | Seed | Endosperm | 28DAP |  | SRP037559 | 13 |
| Maize | Seed | bulk | 28DAP |  | SRP037559 | 13 |
| Maize | Seed | embryo | 30DAP |  | SRP037559 | 13 |
| Maize | Seed | endosperm | 30DAP |  | SRP037559 | 13 |
| Maize | Seed | bulk | 30DAP |  | SRP037559 | 13 |
| Maize | Seed | embryo | 32DAP |  | SRP037559 | 13 |
| Maize | Seed | Endosperm | 32DAP |  | SRP037559 | 13 |
| Maize | Seed | bulk | 32DAP |  | SRP037559 | 13 |
| Maize | Seed | embryo | 34DAP |  | SRP037559 | 13 |
| Maize | Seed | Endosperm | 34DAP |  | SRP037559 | 13 |
| Maize | Seed | bulk | 34DAP |  | SRP037559 | 13 |
| Maize | Seed | embryo | 36DAP |  | SRP037559 | 13 |
| Maize | Seed | Endosperm | 36DAP |  | SRP037559 | 13 |
| Maize | Seed | bulk | 36DAP |  | SRP037559 | 13 |
| Maize | Seed | embryo | 38DAP |  | SRP037559 | 13 |
| Maize | Seed | Endosperm | 38DAP |  | SRP037559 | 13 |
| Maize | Seed | bulk | 38DAP |  | SRP037559 | 13 |
| Soybean | Cotyledon | Bulk | VE | stage is estimate | SRP038111 | 14 |
| Soybean | Cotyledon | Bulk | VC | stage is estimate | SRP038111 | 14 |
| Soybean | FlowerBud | Bulk | V6 | stage is estimate | SRP038111 | 14 |
| Soybean | FlowerBud | Bulk | R2 | stage is estimate | SRP038111 | 14 |
| Soybean | Flower | Bulk | R2 | stage is estimate | SRP038111 | 14 |
| Soybean | Flower | Bulk | R3 | stage is estimate | SRP038111 | 14 |
| Soybean | Flower | Bulk | R3 | stage is estimate | SRP038111 | 14 |
| Soybean | UnifoliateLeaf | Bulk | VC | stage is estimate | SRP038111 | 14 |
| Soybean | TrifoliateLeaf | Bulk | V6 | stage is estimate | SRP038111 | 14 |
| Soybean | TrifoliateLeaf | Bulk | R6 | stage is estimate | SRP038111 | 14 |
| Soybean | LeafBud | Bulk | VE | stage is estimate | SRP038111 | 14 |
| Soybean | LeafBud | Bulk | VC | stage is estimate | SRP038111 | 14 |
| Soybean | LeafBud | Bulk | V6 | stage is estimate | SRP038111 | 14 |
| Soybean | Pod | Bulk | R3 | stage is estimate | SRP038111 | 14 |
| Soybean | Pod | Bulk | R3 | stage is estimate | SRP038111 | 14 |
| Soybean | Pod | Bulk | R4 | stage is estimate | SRP038111 | 14 |
| Soybean | PodAndSeed | Bulk | R3 | stage is estimate | SRP038111 | 14 |
| Soybean | PodAndSeed | Bulk | R3 | stage is estimate | SRP038111 | 14 |
| Soybean | PodAndSeed | Bulk | R4 | stage is estimate | SRP038111 | 14 |
| Soybean | Root | Bulk | VE | stage is estimate | SRP038111 | 14 |
| Soybean | Seed | Bulk | R4 | stage is estimate | SRP038111 | 14 |
| Soybean | Seed | Bulk | R4 | stage is estimate | SRP038111 | 14 |
| Soybean | Seed | Bulk | R5 | stage is estimate | SRP038111 | 14 |
| Soybean | Seed | Bulk | R6 | stage is estimate | SRP038111 | 14 |
| Soybean | Seed | Bulk | R6 | stage is estimate | SRP038111 | 14 |
| Soybean | ShootApex | Bulk | V6 | stage is estimate | SRP038111 | 14 |
| Soybean | Stem | Bulk | VE | stage is estimate | SRP038111 | 14 |
| Soybean | Stem | Bulk | VC | stage is estimate | SRP038111 | 14 |
| Soybean | Cotyledon | Bulk | VE | stage is estimate | SRP038111 | 14 |

**Fig. S2 A-L** Expression profiles of endogenous maize and soybean genes mined for expression elements. These charts are generated from maize and soybean expression atlases, which were built using extensive transcriptomic data. The maize and soybean atlases are comprised of 209 and 100 individual tissue samples, respectively. The X-axis shows tissues grouped by organ. The Y-axis values are log_2_ Fragments Per Kilobase of transcript per Million mapped reads (FPKM). For clarity, only relevant reproductive tissues are labeled.

**Maize**

**A** Zm00001d017180

**
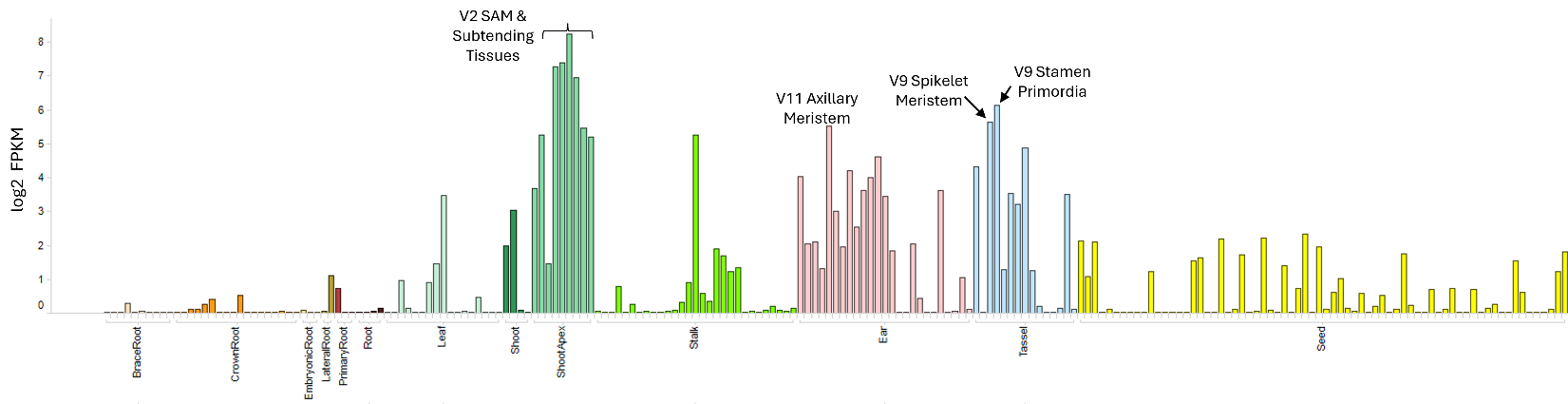
**

**B** Zm00001d022956

**
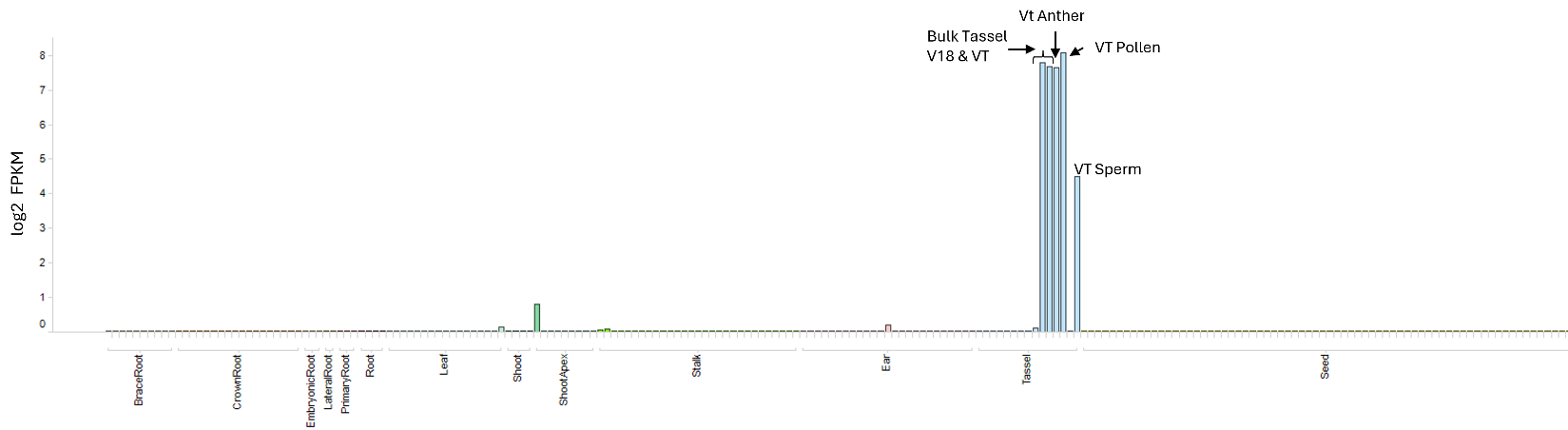
**

**C** Zm.BA1

**
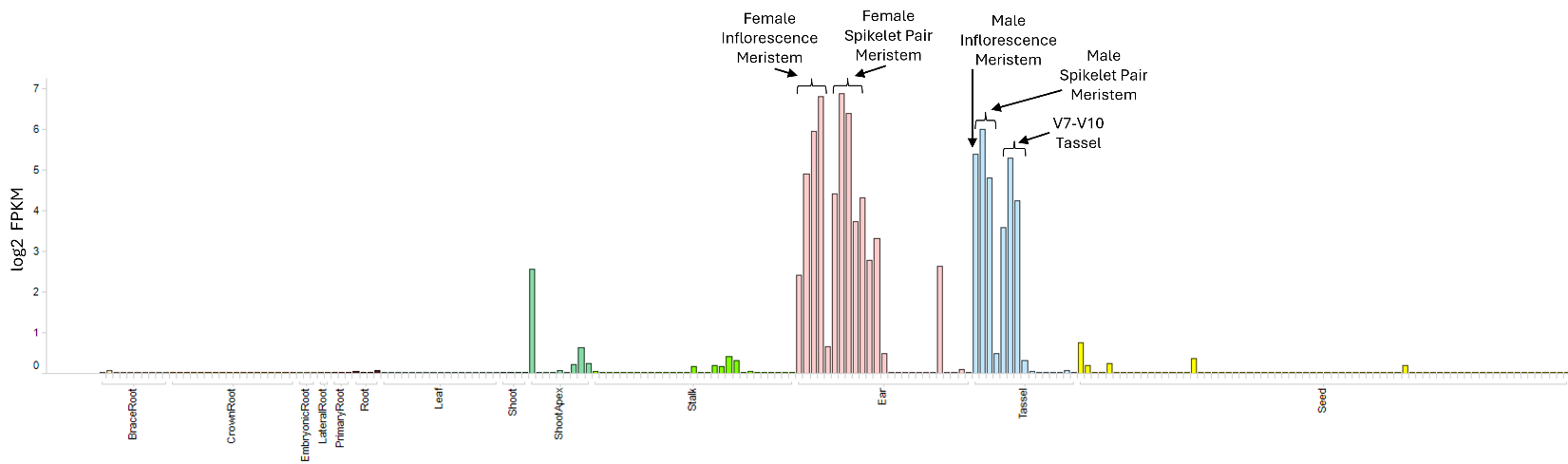
**

**D** Zm00001d007419

**
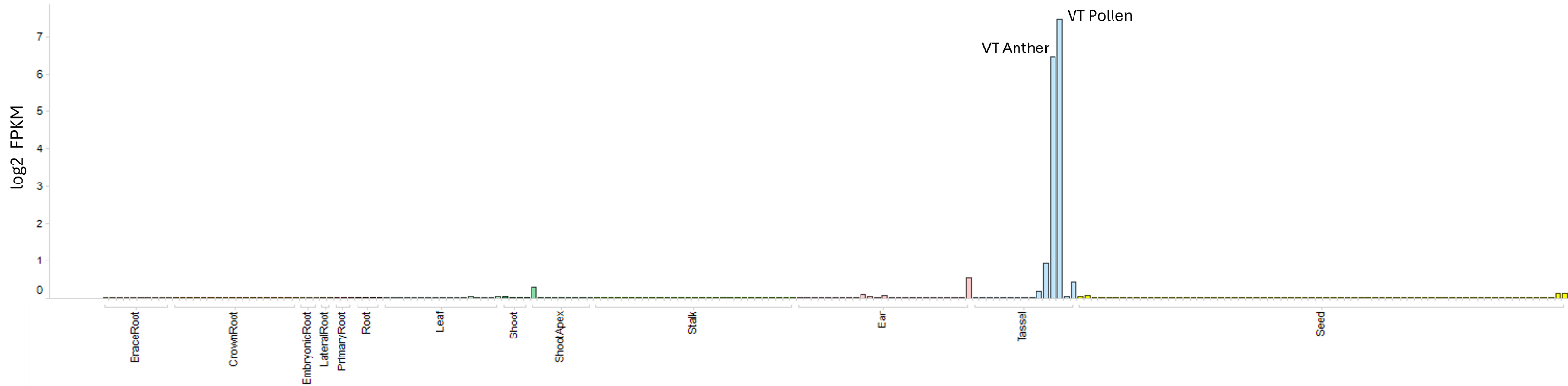
**

**E** Zm00001d043589

**
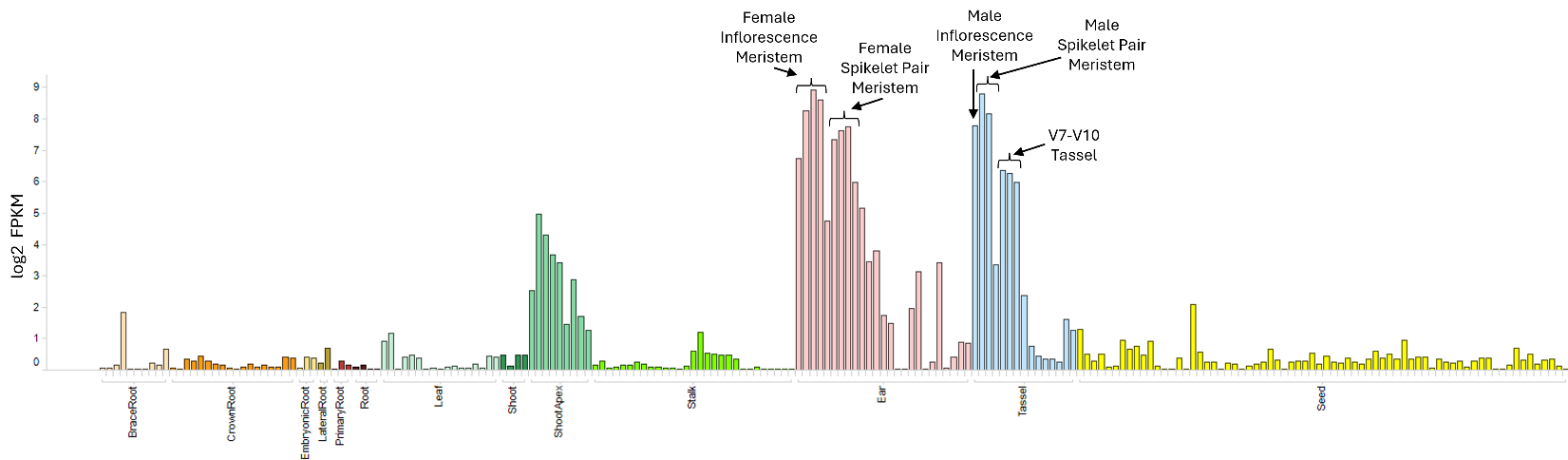
**

**F** Zm00001d024378

**
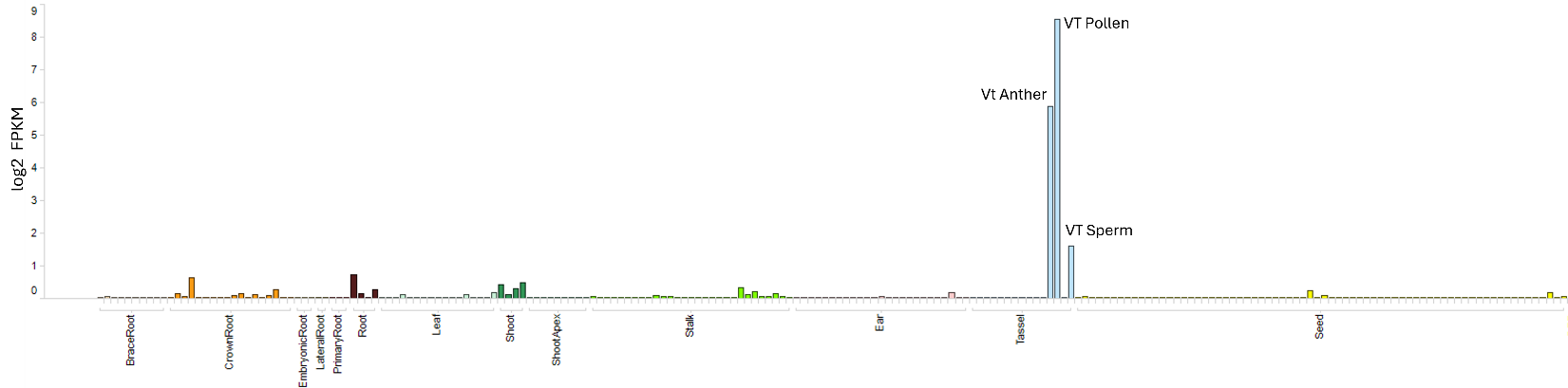
**

**Soybean**

**G** Glyma.CALa


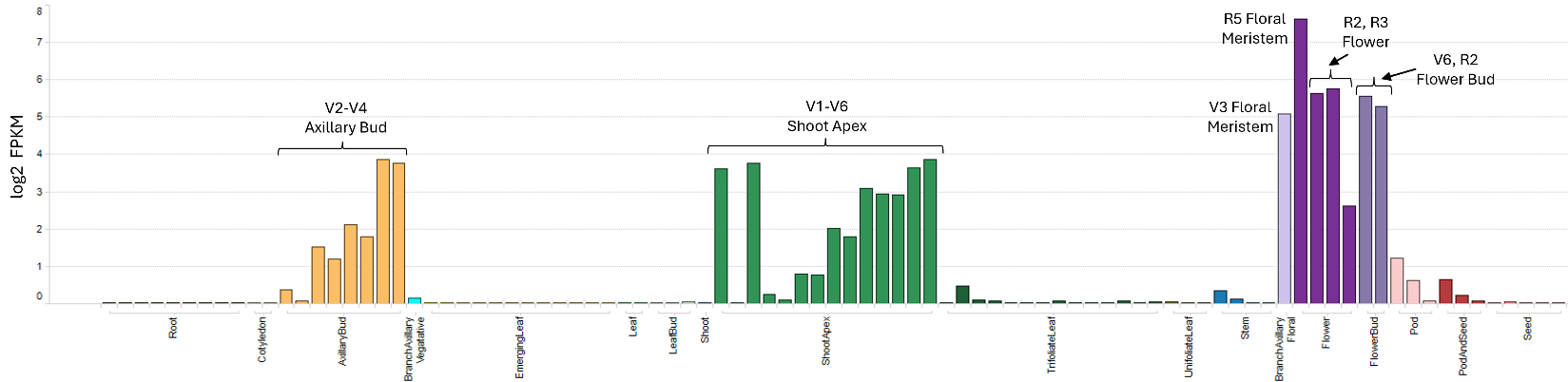


**H** Glyma.08G250800


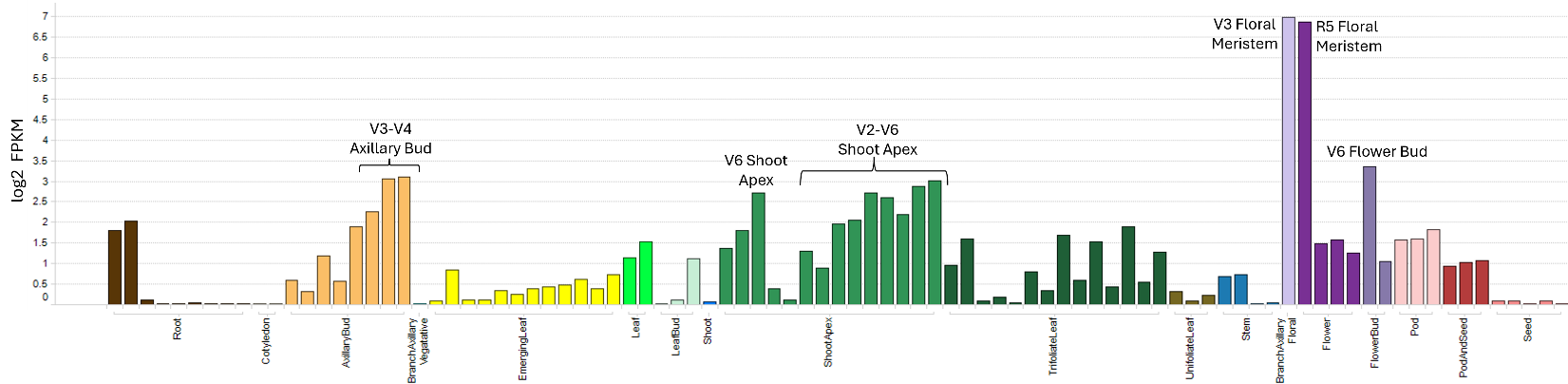


**I** Glyma.10G071400


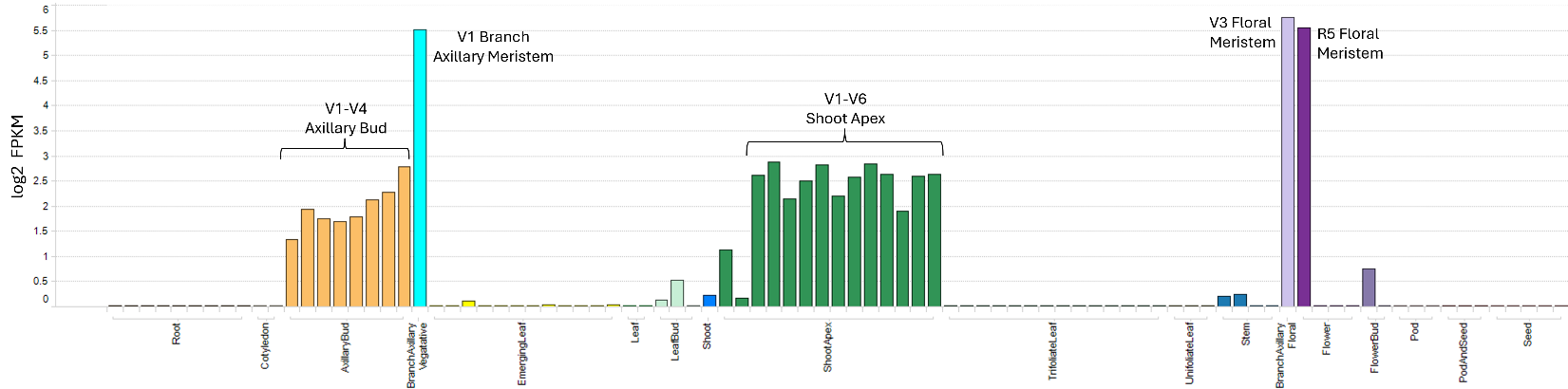


**J** Glyma.11G209500


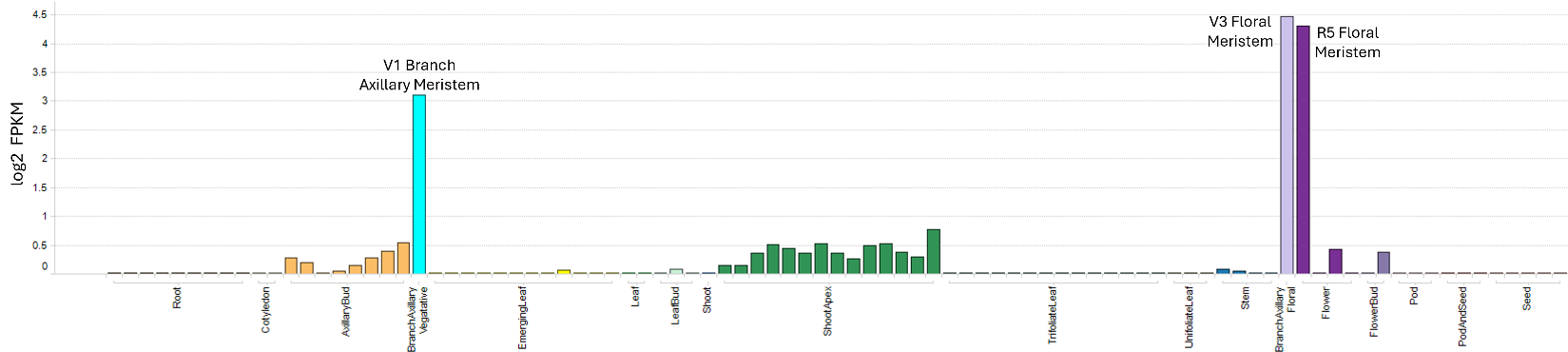


**K** Glyma.AP1


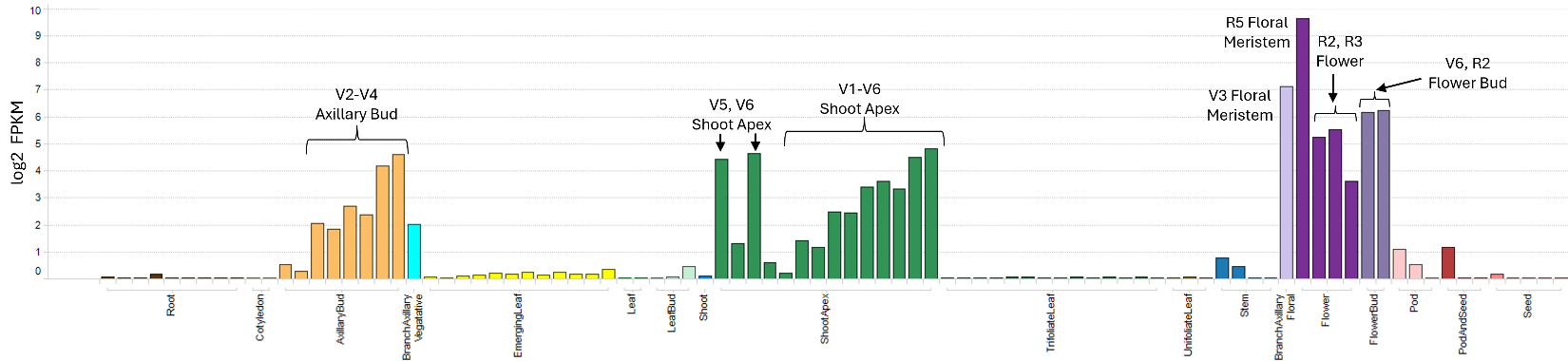


**L** Glyma.16G200800


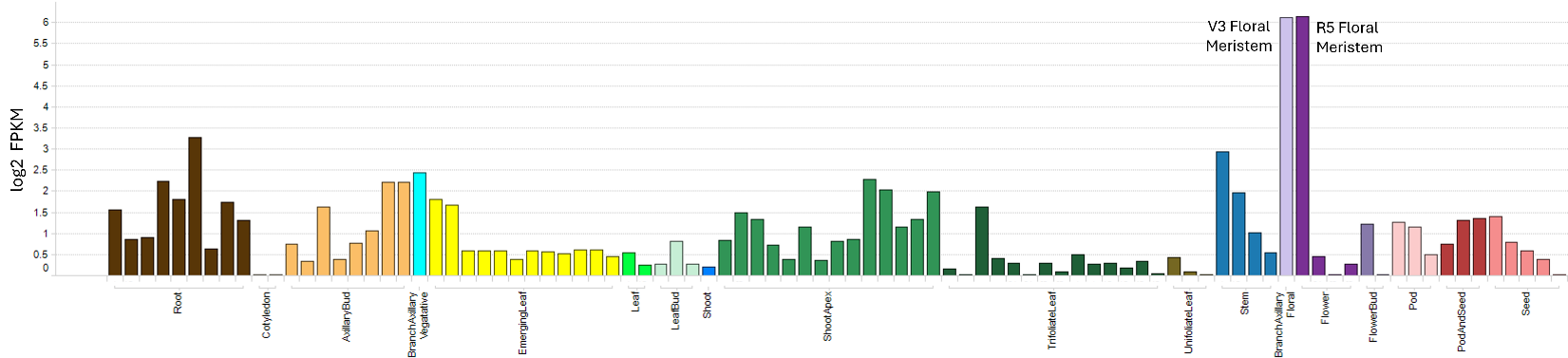


**Table S3** Summary table of mined maize and soybean genes, with target reproductive tissues. Only reproductive tissues with predominant expression for a given gene are included.

| **Crop** | **Gene** | **Target Reproductive Tissues** |
| --- | --- | --- |
| Maize | Zm.Traf29 | Egg, Sperm, Early Embryo |
|  | Zm00001d017180 | Axillary Meristem (Female), Spikelet Meristem (Male), Stamen Primordia |
|  | Zm00001d022956 | Pollen, Sperm |
|  | Zm.BA1 | Inflorescence & Spikelet Pair Meristem (Female & Male) |
|  | Zm00001d007419 | Pollen |
|  | Zm00001d043589 | Inflorescence & Spikelet Pair Meristem (Female & Male) |
|  | Zm00001d024378 | Pollen, Sperm |
| Soybean | Glyma.CALa | Floral Meristem |
|  | Glyma.Mads17 | Floral Meristem |
|  | Glyma.08G250800 | Floral Meristem |
|  | Glyma.10G071400 | Floral Meristem, Branch Axillary Meristem |
|  | Glyma.11G209500 | Floral Meristem, Branch Axillary Meristem |
|  | Glyma.AP1 | Floral Meristem |
|  | Glyma.16G200800 | Floral Meristem |

**Table S4** Summary table of expression elements and coding sequences in test constructs.

| **Crop** | **Construct** | **Cre Expression Element** | **Source Gene** |
| --- | --- | --- | --- |
| Soybean | pMON896 | Promoter and 5’ UTR | Glyma.CALa |
|  |  | 3’ UTR | Glyma.CALa |
|  | pMON897 | Promoter and 5’ UTR | Glyma.08G250800 |
|  |  | 3’ UTR | Glyma.08G250800 |
|  | pMON892 | Promoter and 5’ UTR | Glyma.08G269800 |
|  |  | 3’ UTR | Glyma.08G269800 |
|  | pMON893 | Promoter and 5’ UTR | Glyma.10G071400 |
|  |  | 3’ UTR | Glyma.10G071400 |
|  | pMON889 | Promoter and 5’ UTR | Glyma.11G209500 |
|  |  | 3’ UTR | Glyma.11G209500 |
|  | pMON888 | Promoter and 5’ UTR | Glyma.AP1 |
|  |  | 3’ UTR | Glyma.AP1 |
|  | pMON894 | Promoter and 5’ UTR | Glyma.16G200800 |
|  |  | 3’ UTR | Glyma.16G200800 |
|  | pMON890 | Promoter and 5’ UTR | Glyma.Mads17 |
|  |  | 3’ UTR | Glyma.Mads17 |
|  | pMON555 | Promoter and 5’ UTR | Glyma.Rsp-1 |
|  |  | 3’ UTR | At.Cdc45 |
| Maize | pMON949 | Promoter and 5’ UTR | Zm.Cdc45-1 |
|  |  | Intron and 5’ UTR | Zm.DnaK |
|  |  | 3’ UTR | AGRtu.nos |
|  | pMON958 | Promoter and 5’ UTR | Zm00001d022956 |
|  |  | 3’ UTR | Zm00001d022956 |
|  | pMON959 | Promoter and 5’ UTR | Zm00001d024378 |
|  |  | Intron and 5’ UTR | Zm00001d024378 |
|  |  | 3’ UTR | Zm00001d024378 |
|  | pMON960 | Promoter and 5’ UTR | Zm00001d007419 |
|  |  | Intron and 5’ UTR | Zm00001d007419 |
|  |  | 3’ UTR | Zm00001d007419 |
|  | pMON971 | Promoter and 5’ UTR | Zm00001d017180 |
|  |  | Intron and 5’ UTR | GSI85 |
|  |  | 3’ UTR | Zm00001d017180 |
|  | pMON176 | Promoter and 5’ UTR | Zm00001d018050 |
|  |  | Intron and 5’ UTR | Zm00001d018050 |
|  |  | 3’ UTR | Zm00001d018050 |
|  | pMON217 | Promoter and 5’ UTR | Zm.BA1 |
|  |  | Intron and 5’ UTR | Zm.DnaK |
|  |  | 3’ UTR | Zm.BA1 |
|  | pMON219 | Promoter and 5’ UTR | Zm.Mtf32 |
|  |  | Intron and 5’ UTR | Zm.DnaK |
|  |  | 3’ UTR | Zm.Mtf32 |
|  | pMON220 | Promoter and 5’ UTR | Zm00001d043589 |
|  |  | Intron and 5’ UTR | Zm.DnaK |
|  |  | 3’ UTR | Zm00001d043589 |
|  | pMON222 | Promoter and 5’ UTR | Zm.Ra3 |
|  |  | Intron and 5’ UTR | Zm.DnaK |
|  |  | 3’ UTR | Zm.Ra3 |
|  | pMON720 | Promoter and 5’ UTR | Zm.BA1 |
|  |  | 3’ UTR | Zm.BA1 |
|  |  | Spacer | ISR4 |
|  | pMON721 | Promoter and 5’ UTR | Zm.Traf29 |
|  |  | 3’ UTR | Zm.Traf29 |
|  |  | Spacer | ISR4 |
|  | pMON276 | Promoter and 5’ UTR | Sevir.SPO11 |
|  |  | Intron and 5’ UTR | Setit.Eef7 |
|  |  | 3’ UTR | Trav.Hsp16.9 |

**S5** Sequences of expression elements and coding sequences in this study. Sequences may include small linkers on ends. Expression elements should be placed immediately next to coding sequences to recapitulate the tested gene cassettes.

>Cre_coding_region_1

ATGTCCAATTTACTGACCGTACACCAAAATTTGCCTGCATTACCGGTCGATGCAACGAGTGATGAGGTTCGCAAGAACCTGATGGACATGTTCAGGGATCGCCAGGCGTTTTCTGAGCATACCTGGAAAATGCTTCTGTCCGTTTGCCGGTCGTGGGCGGCATGGTGCAAGTTGAATAACCGGAAATGGTTTCCCGCAGAACCTGAAGATGTTCGCGATTATCTTCTATATCTTCAGGCGCGCGGTCTGGCAGTAAAAACTATCCAGCAACATTTGGGCCAGCTAAACATGCTTCATCGTCGGTCCGGGCTGCCACGACCAAGTGACAGCAATGCTGTTTCACTGGTTATGCGGCGGATCCGAAAAGAAAACGTTGATGCCGGTGAACGTGCAAAACAGGCTCTAGCGTTCGAACGCACTGATTTCGACCAG

>Intron_potato_LS1_used_in_Cre_and_GUS

GTAAGTTTCTGCTTCTACCTTTGATATATATATAATAATTATCATTAATTAGTAGTAATATAATATTTCAAATATTTTTTTCAAAATAAAAGAATGTAGTATATAGCAATTGCTTTTCTGTAGTTTATAAGTGTGTATATTTTAATTTATAACTTTTCTAATATATGACCAAAATTTGTTGATGTGCAG

>Cre_coding_region_2

GTTCGTTCACTCATGGAAAATAGCGATCGCTGCCAGGATATACGTAATCTGGCATTTCTGGGGATTGCTTATAACACCCTGTTACGTATAGCCGAAATTGCCAGGATCAGGGTTAAAGATATCTCACGTACTGACGGTGGGAGAATGTTAATCCATATTGGCAGAACGAAAACGCTGGTTAGCACCGCAGGTGTAGAGAAGGCACTTAGCCTGGGGGTAACTAAACTGGTCGAGCGATGGATTTCCGTCTCTGGTGTAGCTGATGATCCGAATAACTACCTGTTTTGCCGGGTCAGAAAAAATGGTGTTGCCGCGCCATCTGCCACCAGCCAGCTATCAACTCGCGCCCTGGAAGGGATTTTTGAAGCAACTCATCGATTGATTTACGGCGCTAAGGATGACTCTGGTCAGAGATACCTGGCCTGGTCTGGACACAGTGCCCGTGTCGGAGCCGCGCGAGATATGGCCCGCGCTGGAGTTTCAATACCGGAGATCATGCAAGCTGGTGGCTGGACCAATGTAAATATTGTCATGAACTATATCCGTAACCTGGATAGTGAAACAGGGGCAATGGTGCGCCTGCTGGAAGATGGCGATTAG

>Glyma.CALa_promoter_and_5_UTR

CTTTCGTGTGTGTGAACAAAATAAATGATCTGCACATGTATCATGGTTATAGTAACAAGTCCCAGCTCCCAAGGGTAGTAGCTGCTTAAAGACACACGGAAACCCTCGAGGATAATGGTGGAGAGAGAGTGTTGAAATTTCAACCATCTCACTTTTTTCTTCTACCTTATTCTTTAGCCGAAAATTAAGTTCTAACTTTAAAATACAGATTTGCTTCAGAAGTTGATTTTTTAGGTTTTTTTTTTTCTGGAAGACCAATATTAATTTGTGATAGTTAGTTAAAAAAATTATATTATGTTTTGTGATAGTTAGTTGAAAAAATTATATTGTGTTTTGCTACTAATTTACTTTCAAAATATAAATATCCAAATATATATATAAATCTTATCACTTGAAAATAGATGGTGGGTATATGGACTCAAATCTATGGATCGGTCTGTTTAACCCACGAATTTTGCAGATAACAAGCCTTTTTTTAAAAGAAAATTCGCATAACTATATCCAAGTTTTTCTTAGTTCGGTCCATTAAGCTAGCACATCTAGTAGTCTTTATCCATGGACATATTATTAATTTGTTATGTTAAAATTTTGATTTTTAATCTAGTGGTATATTTTAGTATGATAGATTTTAACTTAAAAAATGAAGTCAATTTTTTATTACTAATTTTATACATTTTTCACTTTTAAAAAATATTTATTTCATAAGAAATATTTTTAAATTACTATTTGGGTCCATGGATCAATTTATTTATCTGAGTTTTTATGGGCTAGATACAAATCTTGGAAAAAAGCCAATTTATTTCATGGATTGGCTTATTTGATCCGTCTAAAATGCAAGCTTCACAAGACAAACCTTAAACATACCATACCATCCTTTTATCTATCTCTACTTCAAAATCAATTTTTGCAACATTCACTCAATGGTGCACTTAATTAACAAACCCTCATCCATATACACATATTTAGAGATCAACAATATGATTATCTATCAAAAATACACAAAATCAGTGTGTGTTTGGGTAGGCGTTGACAAAAATTAGTTTTGAATGAAATTGATTTTATAATTTTTTTTTTGTTAAAATTGATTTTGAAGTAATATTATTTATGTTTGGATGTTTTATTAAAAAATTAAGTTATGAATAAAATGAAGTACATAATTTTGGACCAAAAATTATTCAAAATTATTTCAACCCAAAATTAATTCTGTATCCAAAGTCAATTTTAAATTTTTCTTTGATGTGAAACTAAACATGTAAAAATGTATTTAAATTAAAATTAATTTTAGACTTATAATTAATTCTCTGTGGTCAATCCAAATACACACTCACACTTAGAATGTATGAAATTTCAATTTTTAACTTTCTCATCTATGAGCTGTTCCTATTCCTCCTTCCCCTCCTATGCCCTCACTAGGGAGCCAGCCAGCCATATTCCAAAAGCCCTTATTATCACACATGGGTCCCTCCATAGTCAAAATAAAAATAATATCATGATCACTGTTTGGCCATAAAGAGCTATACGACACACATGGACACAGTAGTACACTGCCCAACCAATCACACGTCGACAGCACACCGTTCCCAAACTACTTCACCTTTCCCAAACCAGAAACCAAAACCACTTGTCATCAAACCCCTGCCCAGATAGTTTTTCTCCATTTCAATATTTTACTTCACCTTTTGAGGCTTTGTGGGTACTACAAAACATAACACAATTGAACTCACTGTGCTTTCCCATGACACACATCTATACTTGTCCAGAAGAAGAAAGATCCATGAACTAACATTCCCACACGCTCGCTTCACCATATTTGCCACCCTTTTAACCCTCACTTCTTGGGTTTATTTTGCTCCTTTTTTTTTTCCTTGTTTGGGGTTTGCATTTTTCCTGGTTGAAAAAGGGAAGAACTTGAACTAATTGGTTAAGTTACCTATCTATCTATCTATCTAGGGTATAATATTTTATATCAGTTATTAAAGGAAAGAAAGAAAGAAACGAAG

>Glyma.CALa_3_UTR

AAATTGTTTCCTCGAGGTGGATAATATTATATAACTGCTGGCATATATGAACTACGTAGTATAAGTACTCTGTTTGAATGTTTTATATGTATTTTGGTCATTGGCTTTTCCCTACTTTGAAGACATTATTTTGAATTGCTTCTGTTGTAAACATATTGAATTTGAAATCTAATTGTGTATTTATGAAATATGTTAATTATACGGTTTTCCTTACTCTCAAGATCACAGAAATTTGAATGTTTTTTTTTGGGTGATTACATCAATTGAAAGATTTTCCTTCTGTAGTATTTTTTTAATTTTTGGTTTTGATCCTATATACTTGATTAAATGAATCTATATATACATTGTGTTTGGTAGGATGAAAAAAAATAATTGAGAAATAAGATGGATGAAAATAGGGAGAAAAAAAATAAAGTGTATAGAAATAGCTAAGAAGTGGAGTTGAAATAAGGGTGCTTAGAAGTGAAATAGGGAAAAAAGGAAAAGGAAAAATAGGAAAA

>Glyma.08G250800_promoter_and_5_UTR

TATGACGCGGAAAAGGCATAAGAGAAATCATAAAGTTGATAAATGAAAAGACGCATGATGACAAATTCACAATCATGGCAGCAGACTTGCTTCTTCCACATTACAGTCGTGGGGAAATTCATTTAGGGCAGCGAATAGTTAATGGACAAAGCATTTTTATTTCTTTTCTTTTTGCCAACTGAAACAGAGAAAAGAAAAATAACGGAATGTTAAATTATCAGCGAGAACTAAAAATATTTGTTTATATAAAAGTGAGCACCAAATAAAATAAATATGGAAATCAAAATCTGATATTTTTTTTTGAAGAAAACATGCTTAATCTTAAAAATTTGAAAAACTTTGGGTTAATTATCTAAATTTTTGCAAAATTCACATTTAGTCTATAAACAAAAAACTTCTAGTCTTTGTTCTTGTACTTTTCCTAAATTAATATTTTTACTCTAGGTCATTAAATGTCACCGTTAAATAATTATGTGTACAATCATATTAACATTCATTTTTCACAACATCAACTAGTCTTTGTTCTTACATGAGAAACACATGTATTTTTTTTATAGACATTATATTTTTAATCCCTTATATTATCATGTCAAACTAGTAGATGATATATTGAGTCAAGTGTTCACCAGCGTGGCTAATGACTATCGTGCCATCAGTACTTAAGAATGATACTTGATAATAAAGACCTATTACAGAAGAAGAATAGGAACTATTGCACTTCATATAGAAAATTGTCGTTAGGATATCGTTACCCAATTAATGCAGTTAGAGGTATACAACCTAGGGACTCAACGCAAGGAATGACATATTACACTTCATACTAAATGTTTTTGGGATTTGGTCTCTTGCTTCGTTAGACATGATCCAGTTACAAAAACCAGAATAAAAATAAAAGAATAAATAAGTTCAAAAAAATAAATAGTACACAAATACTTTTTCAAATATTTTTTAAAAAAAAGCACACACGCACCCACAAAAGACAAAACACACGTAAATGCAATATTGAATTTAATAAAAGTTAAATAAATAACACACTAACACAAACACATAGTACAATTTTGTGTTTGTTATCTTTTTGTCCTTTTTTTAGATTTCTGTTTTCACTCCTTTAAAAAAAATTGTTCCGATTTGTTGGTCCAAATGTATTTTTTTTGAGAGATTAAAAAAGTTAATAAACCTAAAAGAATATATAAAACGAAGATGGGTCTGAGTTGATCAGCCCGAAATACTGCATGCACCTCACAAATTGACAAGTGCTTTTTTCCTTTTAATATTCTCTTGATTTGTTCTATTTGGGAAGAAGAAGAAGAAGAAAAAGAAAGGCCTATACTATAACACTACACTACAGTGAGGGGGCGCCGTCAACCAATGAGAAGCGAAGGCTGAGTGCCACAAAGACAACAGAACAAACCCGTTTCCCGTTTCCCGTTTCCCGTTTCCCGTTTTCTCACTTATCCATGGGACCCACATCATCGTACGGTCTGTCTCCAAAACGTTACGCCCTGTCAACACGACACATCAATACTCTCACACTCTTGTTCAATTCTAGCTCGACAACGCATTGTACCTTAACCCTTTAACCAATCACAACTCGACAACGCATCGTACCTTAATTCCCCCTCCTTTCCCCAATTTTTGTTCTTATTCTTCTTTATTTTTTCGTTCACAATTAATTAAAGTAGCCATTATTTCCGAAGCACAAAAATAGAAAAAGGAAATTCCCATGCTATCCTATTATTAGACATCCTCCACTTGTTTTGCTTTCATGTTCCTTTTTTATTTTTCTCTTTCTCAGTCTTCGTTTGCTCAGGTGAATACCACTCACTCTCTCTACTTCCTCTTCTAGCTAGGGTTTCCTTCTTTATACAAAATACAACCTAACAAGTAACAACCTTCTTTATATGTACATATTCCTTAACCCTTGTGTTTCTCTTTTGAGCTAATTCTATTTGTGCTTTTCATAGAATTGCTAGTTACGTTGGAAAATTAAGCAAAAGAAG

>Glyma.08G250800_3_UTR

CTAGTTAGGCTGGCATGATTCTGCCAAGTATGTGACATGTGATATTATGACAAAGGCTCAACTATATATATATGTTAGAACTTAGAATAGCCAATTATATGCCGATCGAGCTTAATAACTGCTCAAACTTCTCGCATGAACTGTGCCCTTCACTTTGAAATCAATGGTATTAAGCAGTTATTTTGACAAATGGCACGTAATGGATGGTGCTAGTTAAATTGGAAGTCATCAAAGAAAACATTTAGCCCACAAAAAAGACACTACGTGATGTGTACCACCACTACCAAAAGAGTTTTTCCCTACAAAGCTCACACTTTTTTTCCTCCGGGTTAGGCTTCATTTATTAAACCTAGTTGGATAAAGATGAATGAAAAAGATTGAAATGAAATGTAACTTTTACCTAGTGATTACGTAGATTATAATTTGAAAAGGTTTTAACAGGCTCGAAGCCGCCGCATGTATGATATTTTGGATTGTGGCCTCTTCGTGGCTACGTTTTT

>Glyma.08G269800_promoter_and_5_UTR

AACAAAAGATCACAAAATTGAACAATAATGACAATACCTCGCATTGAAGTAGTTCAATACATATTCTGAGTATGACTCGCGAGTACATGTATCTCATGTTTCAGTTTTTAACCACAGCTGGATGCCATGGACACTTTTAATTCTATTCTTTTAAATGAGATACCTGGCCTATCAATTTTAAGGGTAAATTAAAACTCAGAAATCAACATACTAAAAAATTGCATGCATGTCCGCAAAAGAAAATGTTTAAGGCAGTATATATTCACTAGTGCACAAAGTTTTGACCTTAAATATTGAAATAGTGACCATACGAGTGTAATGTTTCAGATATATATAATGCTAATTATGGACTAATTAATTAAAATAGAGGATTGCTTTCAATAGGAAAATAGTAGTGTAATGGAAACCGGAAAGTGAACTAGAAAGAAATGTTAAAAATGGTCTAGGGAAAAAGTTAAGGAGAAAATGGATTATACATAATGTCAAAAATTTTATACCGCTGATTATAATTTATATATTATGTAGGAAAAATTTATTAAATTTTAAAATAATTAAAATTATAATTTATGATTGAATGATATTATAAAACAATTTTACACAATCAATTTATAAATATTAAACTTAAAAGATAAACCTAAAAAATACTATTTTTCTTTATCATAATATTAGTATAAAATAATTCTACTAAAAAAAATAATTAATATTTATAAAAATACATATAAAATAAATATTTTTTTTATAACATAATTTATTTTATATATAAAAATTAATCAAATATTTTAACTTTGGATTACTTAATTAAAAAATGATTCCTAAAAAAGACCATTTGAATATTATTTGGTGGACGAAGGGATGGACCAGAGTTCAATACTCGTGGGTTTTGGCGGCGTCAATGAGAGGATGCACGTAATGTTCGGGTCCGTACGGCATTGCCACTGTTTAAACCATCGGACACTTGAAAGCTCAAAGCAGTGACTGTGATGAGCAGCTCGCAATAGAGAATCCAAAATTCCCAAAAGCAAAAACAAAACCCAAAAAGCAAAAGAGATATCATTGTGTGGCTGTTAGATACCACAGACAGCAACATGGTAACTGACCACCCAATCCGGGCTCGACAGCGCAACGTTAGCAAAACCGCCCTGGTTTTTTCCCAAACCAGAAACCACTTTATCGCCAAACCCCAATGTGATTAGAGTGTTGCTAGGTGCACCTACTGTTATTGCTGGTGCACCCAACCTTCTTTGATAATGATAAAATTATCCGTCTTCTTTTTCCCTTTACGGATCAAGTTGATCCGCAAGTTGATTTTTAAGACTTACAGATCAAGTTAATCGGTAAGTCTTTTATGGATCAACTTGATCCGTAAGTCTTAAAAATTAATTTACCAATCAACTTGATCCGTAAGAAGCTTGCAGATCAAGTTGATCCATAAAAGACTTAAAAATCAATTTGCGGATCAACTTGATCCGTAAGAAGCTTACGAATCAAGTTGATCCGTAAAAGACTTACGGATCAACTTGATCCGTAAGTCTTAACAATCTACTTGCGGATCAACTTGATCCGTAAGAAACTTGCAGATCATCTATATCAACTGTAGATCAACTAAAACATATGCGGATCGACTCATTACATATGTGGACCAACCTGAATGAGCTAGGTGCACAAAAATATTTTTAAAAATACATAGGGATATTTTTGTCTTTTCATGTTAAGTGTTAGGTGCCCAACAACAATAATGCTGGGTGCACTTAGTAACACCCATGTGATTAGGGATATATAACAACATAGCATACTCACACTACTACTCACTGCGGTTTTTTTCTCCTCCAGAAGAAGAAAGATCCTTAAATCAACATTCTCTCTCCCTCCCTTGGTTATAATAGCAAGCTTTTCAAGCATATTTCCGGGGTTTGTTCCCTTTTCCTTGTTTAGGGTTTTCTTTGGAAAAAAAAGGTAATTAGGTATAATAATATAATTAAAGCAAAAAAAAAAAAAATAAC

>Glyma.08G269800_3_UTR

ATACTTTCTTAGAGGTGGATGATATTATATAACTATATATGTGGTATGGCATTTGACAACATATTTGAGGGCTTATATATGCCTCTATGTTTTGTGGTTTTCCCTAATCTGAAGACGCTAATGTTTATTGTGTGTTGTAAGTTCATAATTTGGTTAGGCTTTCTTAAGTGGAAGCTTTGCTATATATATTTAAGTTCCTGAAAGACAAGTCTCGTATTTGAATTAATTATGCGATGAATTTGTTTTTGTTTGCTAGTAATTGATGGAATCCTAATTTGTGATCTAATTTTTAGTATCAATAAATTGGGAAACATACTTGAGATTCTATGAAATTATGAAAAATAAGAAGGAAAAAACGAAAAAAGTAAATATAAAGAACAATGTCACAATCTTGAAAGATTGATAAGTTGGGGATAATTCGTTACAAAGAATAAAATTCTTTGATAAGAACCTAAAGTTTCACACAAGAATCAAGAGATATTCTCCTAAATGTGTCTCAT

>Glyma.10G071400_promoter_and_5_UTR

TTAGAAAATTCTTATTTTGTAGTGATTTTGCAAATAGATGAATAATAGTATTTATTAATTAAATTAATTTTAAATTTCCTAAAATTCAAATTTTCAGATCTATATTTTAAACTATTTTTAAATAATTTCTTTATATTTCACTTATATTTTTAAACTGATATTTATGAATATCCGTATAATTTTCAGAATTCTTGAAAGTCCGTGTACAAATAACAAAATTCCAATGGTCGCACCTGGTTTTGAGTTCCGTACTGCTCATTCTCAGAAGTTTCTCGTTGAGCTCGTGTCTTCCATATATCTAGTGTTCTACTTTGATTTTAATGATAACAATAATAATAATAAATCACTTACTTTCTGTAAAAGAAAAAATCACTTACCTGCACTTGCACATGTCTTAAAAGATTAAAAAATATATAGTCTTTGTCTCGTTTGTTTTTCTTTTAAAAATGATTATTAAATGTGTAAACTTGTGAGAATTTAATCTTGATTAAACATAATTTTTGATTCAGTTCCTGCATGTGTAAAAATATGTTTTAACTTATTGTTACCCGAATTAATGGAAATCCATATTAATTTGCTAATTAAGACTCAGTAAAATTAAATATGTTTCTTTTGTACAATAATGATATTAAGATTTAAATCTAAAACCTTATTTTTTTATAATAAATCTAAAACCTTATTCAAACTATCCGAACCTTCAAATATGAAAGAATTAATTGATAATTGATAATTTTATACAAAGGAAAACGTTGAATCATATCATTCTTCTTAATTTTCTTATTTTTCCACTTTCATTTACACAACATTATAATTAGACACTAAACTACCACTAATGCCCTTTACAAATTGTAATTTGAAACACCAAATTAGTCCAAAGCTTAGCATGAACCAACCTCTATACTGTAACTTTGTCTTACTATAAGTAGGTAATTAATAGATAGTTTTTCACCGTGATTTTAATTTCTTTGATATACTAGTAAATTTTAAAACTTCAACTTATCAAAATAGAATAACATATTTGAGAAGACGATTATTCTTTATTGTTGACAAATTGTAAGAATGTGCTTTGCAACATTTTGGATTGGTGAATGTATTGTGGATCAATAATAGAGAAGGAAAAAAAAATCCTATACATCCCAAAGATATCAAATCACTGTGACACTGCCGCAAACGCCATTACCCACAGTGGACATTAATCGTGAGACATTCTGTGAAAGAAGAATACAATGAAAAAGAATGAACCTTAATTTTTTGTGTGTAAATTATCAATTTAGTCATTTAATATTTACTTTAATTAATTTTTATTTTAGTTCTCTTTGTTCAAAATGACAAGTTTTTGAGATATTTAGTTGTCAATAAAGGCTTAATAACGACATCAATGTCACGTACAAAAACGTAAATGCATGCACTTTCATAAGGTAACAAAATTACCATAAAGAAAAATATTGAAACTAAAAGTTAAGTTAAAAAACCAAATTGAAAAAAAAGAGAGAGAACAACAAAACTGACTTAACATCGAAACGTGTACTTTTAGGTATCTTTGACAAAATTAACTAAAAAACTTATTGGTAATAAAAAATTGTTAATTGAAAGTTTTTAAATTAACTCATTAAATCATAATTACTTGATAAAATTATCTGTTAAATTTTATATCTAAAAAGATAATAAAAAAATTAAATAAATATTTTAAAGATAAAAAATGATAATAAAAAATTTAAAACTAGTATTTTAAAAAATATTACTTCACATAGCATTTTAAAAAATATTAAAAATTAATTAATAAAAAATTTATTTATCAAACTATTAAACAAATTTTTTTAATTAATAAAAAATTAAAAACTAACTAAATAATTTTTTCCTAAAAGAAACCTATTTTAATATATAGTTGCCTTTATTAAGAGGCTATAAATATGTAGTTCATGCTCCCATTTCCCTTCACAAGTAATCAATCATTAAGTTCTATTCTCTTTCCTCTCTTTTTTTCTCTTGTTGCCCTTACA

>Glyma.10G071400_3_UTR

CTAGTAGCTAGATATAATAGCTACTAGTGCAATAAGCAATTAAATGTACTAAATAAAATATGTTCACGTGGGGGACCTATATATGTGTGAGGAGTTCTTCTAGTGCTAAATCTTCATGTGGCTTGCACAAATGTTGTCCTTTTTTTTTTGGTCTTGTAACTTTTGGTGCGTTTGGTGAAAAACCGTATGTGGTTTGTATGTTTTGTTAGGAAGAGTTATATGTCATGTGTCATAAGGGTGAATTATTCCAAGACTTGTGGAAATTGAACATTCCTATAACTATTTGGAGTTATAACTAACCCTTCATAAATAAATGGAATTATATATAAGATTTATGTAATTTCTCTTGAATTCTCCATCTCATGTATTTGGAGTTAATTTGAAGGTGAGGATAATTAATTAGGATTAACGAGTTTTTATCTGCCTGCATCCTTATCCGCTCTATGATTTTTAACATTTTATAAACGAGGTGCTAGCTCTTATTAGAGGAGTGGCACACC

>Glyma.11G209500_promoter_and_5_UTR

TCTCAGAAGTTTCTGGTTGAGCTCGTGTCTTAATAATAATAATAAATCACTTTCACATGTCTTAAAAGATTAAAAAATAGTCTTTGCCGGGATTGTTTTTGCTAAATGATTATTTTGTTCATTAAATGTGTAAATTGATAAATTTTTTGTTTTTGAAAGAATATAAATTTTATTGTAGTCTATGCATGTGTAAAAAAATATAACAATTATGTCTTCTACATATTTAATGATAAATTGATATCTATATTTTATAATTTAATGTCAAATCAATTCTTGTATTGTATTAATTGTTTGTCGACCGACCTAAGACTTCAAGTCTCATCATATTCACACACTCGTGGCTAGAGGATTGTATATAATATTGGTTGTCCGTTGATCGACCTAAGACTTCAAGTCTCATTGTACTTGTGTATTTAAGGCTGGAGGACTATGTACTCTATCTGTTGTATGTTGTCCGGTCCAAGACTATGTACTTAAGGCTGGAGGACTATGTACTCTCATCGTGCTTATGCAGTTGAGACGGAAGGATTGTGTATCGTAACAAGTGTTTTTCGTTTGACTCAAGACTTTATTAAACCCCATCATTCTTAAACACTTGAGACTCGATATATAATTGTGTACTATATTGGTCATTCATGGACTGATTGACAATCAAATGACAATTAGTATCCCATTCTAACGTATCGTTATAAACAATTAAAAATCTTAAAGAGAAAATGCTAGTAATATTAGATTTAACACTTACTTTACTATTAGCTAAAATTTATTGTAAATCACTCATTTTGATGGATCCCTTTCTCATATAATGACATCGGACCTGAATTAAAAGTATGATTCATCAAAATTGATGATTTCCAATCAATTTTAGACAATAGTAAAAATAGGATATAAAAAAGAGTGTTAGATATAATGTTTCTAGCATTTCTCACATCTTAAAACATGGTTTAATTTTTTGGAGAAAAAAATTATATTGAAACTTAAAAATAAATATAAAGAATTATTTGATATTTTGATAACTTTATACTAAGGAAAACCTTGAATCATTCTTCTTAATTTTCTTAATTTTCCTAATTTTCTACTTTCATATACACAACATTATAAGTGGACACTAAAGGACTAAACTACCACTAATTAATGCCCTATACGGCTATACCCACCGATGGACATTAATCGTGACACATTCCGTGAAAGAAGAATAAAATGAAAAAGAATGAACCTCAATTTTTGTGTGTAAATTATCAATTTAATCATTTATATTTACTGTAATTAACTTTTATTTTAGTCCTCTTTTTGTTCCAAATAACAAGTTTTAGAGAATTTAGTTGTCAATTAAGGCTTAGTTAACAACATCAATATATGGCAAGTAAATGCATGCACTTTCATTAAGCAAAGTAACAAAATTAACATAAAGAAAAATATTGCAACTAATTAAAAGTTTAAGTTGAAAAAACAAAATTGATAAGAAAAATAAAAGATAAAACAACAAAACTGACTTAACAACGAAAGGTGTATTGTTACTAAAAGAAAAAGAAAAAGAAACCTATCTTAATATATAGTTTCCTTTATTAAGAGGCTATAAATATGGAGTTCGTGCTCCCATTTCCCTTCACAAGTAATGAATCATTAAGTTATATTCTCTTTCCTCTTGTTTTCCTTGTTGCTCTTACA

>Glyma.11G209500_3_UTR

CTAGCTATATATATATATATATATATATATATATATATATATATAGTTATAGTTATAGTTACTAGTGCAATATATGCAAGCATTTAAATGCACTAAATAAAATATATGTTCACGTGGGGGACCTATATATGTGAGGATTTCTAGTGTAAATCTTCATGTGGCTTGCACCAATGTTGTCCCTTTTTTTTTCGGTCTTGTAACTTTTGGTGCGTTTGGTGAAAAATCGTACGGTGGTATGTATGTTTTGTTAGGTAGAGTTATGTCATGTGTCATAAGGGTGAATTATTCCAAGAGTTGTGGAAATTAAACATTCCTATAACTATATGGAGTTAATTATAATTAACCCTTCATAAATAAATGGAATTATATATATATTAGATTTATGTAATTTCTCTCGAATTCTCTTTCTCGTGTATTCGGAGTTAATTTGAAGGTGAGGATAATTAATTTGGAATAAAAAGTTTTTATCTGCCTGTATCCTTATTCGTTCTATCATGTTT

>Glyma.AP1_promoter_and_5_UTR

AAAAAAATAAGCATTTGCTTCAACAAAATCAAAAAGCATATACAAATAAACGCCAGACTAAAAAGAGGCACACAGGAAAATACAAATTTTGGAAACAATGCAACATAATCGAACACAACAGTCACCCACAGAAACATCGAACCTTTTTGGTTGCACAAAGTCGTGAGAAGACGAACTTGCAGAAAGATATGGCCTTTTTTCGAACAAAGGCATAAACGACAAACAACAGAATCAATGTCTAAAGATAAACATTAGAAATATGAGGTTCAGGAAATAATAGCAACCATCCATCCAAAAATAAGGCAAAAAAAATAAACATGTGCTTCAACAAAATCAAAAAGCACAACAAAAATCGCACAAAGGTTGAACCTTTCTTCTTGTTGTACAAAAAGTAAAAAACAAAAAACAAAAATACAAAAAGATTGAACCTTTTTGGTTGAACAAAGTCATAAATCGCACAGAGAAGGGAAAACATGGCCAGAAAGGTCAGGAGAACCCAAAAATAACCGAACGCAAGAGGTACCCACATGAAAATACATACCTGGCCAAAAAATAATGGTGACAAACACAAAGCTCACAAAGCCAAACACAAAGGTCACAACTTTAATGATGGATAGGAACACAAAGGTCACAGCGCTACACTGATGGAGAGGAACGAAAACCTCTAAACCATGGAGAGGAACGAAAATGAAAAAAGGAACGGTGAGAAGCCGAGAGGAAAAAAAAGGTCACAACCACGGCCATGGAAAAGAACGAAGAGCTGTGGAGAGGAACAGAAAGCAGTGAAGCTACGAAAATGAGAAACTGAATGGTGAGTAGCCGAAACCCTTCGGTTACCCAAGCGCACCGGGAACCAAAACGCGTGTACGCGTACCACGTATCCCAATTCCATAAGAAATTCTAAAAACCCACACGAACCATTTTGGGCAGGAACAAAATCCCCCAGACGACGACACCTCAGAAAGTTAGGGGCATTTTGGTACTTTCTGCATGTGTTAGAGCCTTGTTTGTATAAAGGATATAGATATTTGTCTAAGGTGTATATTGTAAACAAAATATGACTTTCCTATTCTTACATGGTCTTTTGTCTTATAAAAGATGATAGTGACCGAGTTGATAATTGTTCACAATAATAATATGTGTAAAGTGATAGTCAGTTAACAAGTGTCGACTACCCGTCGATTCCGTACAAGATAGTAATCATACGAGTGTAAAGTTTCCTATAGAGAGAGAGACATGAATACTAAGGGGGATTATTTAATTAAAATAGAGGATTGTTTTAAATAGGTAAATAATAATAATAATAATAATAATAATAATAATAATAATAATAATAATAATAATAAGCAAAGGAGAAAGAGATGTTAAAAATGGTGGAAGGAAAAAGATAACCCTAAAAAGGAACATTTGAATATTTGGTGGACGAAGGGATGGACCAGAGTTCAATACTCGTGGGTCGTGGCGCCGTCAACGTAAGGATGCACGTAATGTTCTGGTTCATATACGACATTGACACTGTTTAAACCATCAGACACTTGAAAGCAAAGCAGTGAGTGTGACTGTGAGAGAGCAGCAATAGAGAATCCAAAATCCCGAAAATCAAAAACAAAACCCAAAAAGCAAAAGAGATATGATTGTGTGGCTGTTAGTTACCACGACAACAACATGGTAACTGACAAGCCAATCAGGGATCGACAGCGCAACGTTAAGAAAACTACCCCGGTTTTTCGTAAACCAGAAACCACTTGTCGCCAAACCCCAATGTGATTAGTGATATATAATAACATAGCATACTCACACTACCACTCACTGCGGTTTTTCTCCTCCAGAAGAAGAAAGATCCGTAAATTAACATTCTCTCCCTCCCTTCGCTATAATAGCAAGCTTTTCAAGCATATTTCTGGGGTTTGGTCCCTTTTCCTTGTTTGGGGTTTTCTTGGGAAAAAAGAAGTAATTAGGTATAATAATAGTATAACTAAAGCAAAAAAAAAGACAGTAAT

>Glyma.AP1_3_UTR

ATACTTTCTTTGAGGTGGATGATATTATATAGCTTTGGTATGGCATTTGACAACATATTTGAGGGCTTATATATGCCTCTATGTTTTGTGGTTTTCCCTACTCTGCAGACGTTAATGTTTATTGTGTGTTGTAAGTTTATAATTTGGTCACAAGGCTAGGCTTTCTTAAGTGGAAGCTTTGCTATATATAGTTAAGTTGCTGAAAGACAGGTCTCGTATCTGAACTAATTAAGCGTTGAGTTTGTCCTTGTTTGCTAATTGCTAATAACAGTATATACAATATATAATTATTTTAAATCGACTTTATAACCTGACCGGCAGAGTTTAACCAGTAATCTATGAACTTTTTAAAACGTAAACTACCCTGGAAACTTAAAGGGAGAAATTTATTAATTTTCTATAACATATACTTATCCATATTGTTCCAGATAAGGTAAATGTTTAATATGTTGTTCTGATAATAAATTTAAAATAATTATTCGCTTTTAGAAATTTTGGTC

>Glyma.16G200800_promoter_and_5_UTR

TCTTTATTATTTATAAAATAAAAAGTAATTGAATGATTTTTTTATCTAAATAATAAATATTTATTTGATAATATATTTTTATGTTTTTAATCAATAACCATACAAAGGCATACAATGAGAATTGTTCTTATTTTAAGAATGCAAGAATTATAAATTTAGGTCATACAATTATTGATAAATAATCAAATTTTTATTTTTTTTATTTCTTAAAAAATTAGTTAATAGTCACATGACTAATTAAACATGATCTAATTTAATTATTTTTTATAATAACTAAGAGTTCTCACATGAAATGTTTTCTACTCATAGGTGTATATTACATAAAGATAAAACTAAAATAATTGGTTTATTTGCTAAAAAAACATTATATTGGATAGGATAATTATAATTGTTTAAGGTTTGATTTGAAAAAGAAAAAAAAAATACATGAAACAATACAACTTACATGGAGATGGACCATATAAAATTTTTAAATTTTGTTTATCAATATATCTTAAGATAATATTTTTTATCTTGAGTACAAAATATGTATCTATATCTATCTCCATCAAACAAAACACTAGATAAAAAAAATTATCTTATAACTTAAGTATTTATCATGTGTTCTTGATGAGTATAGATTGTTAGCATTTCTCTCTCGCACTCTTTCAAGATTGTAAAATCATGTTATAAAAATGTGTAAAAATATGAACATATTATCCACTTTTTTCTTTGTTTTTAACTAAGTCATGTCACAAACATTGTTTTTGAGATATTAATTCTTAACTCTAACAGTGTGGTGATTATAAAACTCTTTTAGAGTGTGTTTAGATCAAGAAATTTAAAATTCTGAGAAATTTAAAATTCTAAGAATTTCAAATACTTCAATTGAAATTCTTTTATTTTCAAAATTTTGTGTTTCGATAAAAAAAGTTAAAATTATGAGGATGAAAAAAAAAATATGATTGGTGTGCTAGTTATACGTGTTCCTCTACACTCAGATCCGATCGATATTTCACAATCTCATGCAGTGTTTCTTGAAGAAGACTGTAAGAAGAGAATTTCAATTTCTCACCTTTTAGAAGGAAATTGAAATTCCAGATTTTTAGTTGTTTAAAATTCTGTTTTAAAATTCCAAAATTTTAAATTCTTCACAAAAAAACATCCAAATAATGAATTCTAAATTACAGAAATTTAAATTATCTGATAAATTACTTTTCTGAATTAAAATTTTCTATCTAAACATACTCTTAAAATTTTTTTTCTTCGGATTTTGAACTTTTGACTTCCACATAGACGTGGGGTTCCATTTCCAAGGCCATCCATGTTGGTAATTTAATTTGCATGCCATGAGTTTAGACATGACAAGGCGAAGTGGGGAGGAGTATTGTCTTTCTAATCTTTGACCCTTGCTTCTCAATATATACTCATATCCATTCTCGATATCCCACATGTTAAGATTTATTATCTTATTTTTATACCGATCCGGTATAAGATATCTTCGTCTCCAATTTAGATTTGTAATTTTTTTTGTTAAAAAATTATATTAAAAATATTATTTAAAATAAAATTGATTGTTACACATTTATTTTTAATTACTTGTATTTGTAGAGTATTTATCTACAAAAATTATCAAAAAAACAATAACAAGTAATTAATAAAATTTTAATAATATCTTCATTTCCGACACCAACCCCGATTAAAAAAGTCAAATAATTTTTAAATTCGTATCCGATCAACTTAGATATTTTTCATCAAAATCGAAACGAATTTATACATATACCCACGAGTTTCATTGTCATGAGTACATTAGTTAGTTGTGTGTAAAGGGCAGAGAGAGAGAGAGAGTCCCTGTGCCTATATAATGCCCCAGTCATTCTGATTTCCCCAATAAGCGGCCTTGGCTCTTCTTTCACCCTCTCTCTCTCGCTCAGTTTCTCTGTTTTCCTCTGCTTCTCTCAAAACACACCTTTTATTATTATTATTTTACTGCTATAAACTAATTTTGCATTGTTAATACA

>Glyma.16G200800_3_UTR

AATGAAATTTCAAGCACATATTATTATTAGCCTTTGGTAATCTCCTATGAATCAATAGTAGCAAGTGTCAAGTGGAACTAGTGTGATTGATGGCGGTGAAAAGGGCAGCAGCAAAAACGCACTGAGTTGGAAAAATTGAAAGAAAATTCATGTTTTTTTCATGATTTGAGTGTTGCACGAGTAGTGATTGGAGTAGTATAACAAGGGATATAATATTTATAAGGGCGTCAATGAGAAAAATTCGCCAGCTAATTTTGTAATTTTGTTTTGGTTATAGAAAAGTTATTGTTAAACTTTCAATATTTTTTATTTTTTGCTTAACGTGCATAACGACATATGATATGGTAAATCCAAGTAAGTAAATTTTCTTAAAGGGTTTGGAACCATTAGGAAGAGACACTAATACTGACTTTATAACTTAATGGCCCTTAACTTCTCTAAGATTCGGAGAAGGACTTGATACTCTGTCTATTCCCAGATACTCCCAATGTAGATATTGC

>Glyma.Mads17_promoter_and_5_UTR

ATAGGTTTGGTCAATGTCCCCCATACTTTTTAATTTGTACATGTTGATCCTATTGTCTTTTGTTAATAATATTTGTTTGGGGTGCTTCAAATGATGGATAGTGCCCGAGATGCTAACTGTTTGGAATCCTTTAGTTATTGTGATGACTTTGTTTCATATTTTTTGCCTTTTTTTATGATCGTCATCTTTTTGCCTTAAAAAAATGTGTATTTATTAATTTAATATACTTTAGAAAAATAATTTAGTTTACTAGTTTCCTTAAAACTTAAAAGAATAATTATTTCCCAATATAATCTTAAATAATAAACAGTTATTTTTATTGGCTATTAAATAATTTTGATCCTTCAATTCATTTAATTTTTTTTATTTAAATTTAGTCCCTTAAATATATGACATGCACGTTCCATTTTAGTCTACCAATATATTTTTTGTTAGACTAAGTTAATTCTTTTTATCAATTGTAGTAAATTAATTTGGTCCACTTGATAATACAACTTGATAATACATGATAAAGACTATTAGATATGTCACATCATATAAACATTTAACATGAGTTAAAATTGCTTGAAAGAAAGCCAGTTAAAATTATTGCTTGATAGCCGAAAAAAATTAATAAAGGAATCATTTTGACACGAGACACTTAAAAAAAAGTATAGTAAATTTTTTTATTTTTTAAACTTAAAAAGGCGAAATTGAAATAAAAAATATCGACCAAAGACTGAAATTAAATTTCAACCATTTTTTATTAAAATACTGGAGTATTATCAGAATAAACAGTAACTTTCCCGTGGCCCCCCCACACAGACAATAAAAGTGGGAGTCCAAATCAAAATTATTTAAATTTCACTTTAAGTTATTAATAGTAATTAACACTAAGATTTAATGTCCAACTTTGTTTTACGTGCATAAAGATCAAACTTTAATTTCAATCGAAAAATAATGTTGAAAGTTTAAACGCAGTAGCACCGTTAAATTTTGGCCTGGCATGAAGGTGTGTTCCATCTCTGCAACACCTCTGGGCCGTGACTCCATGCAACGTGTCGACACTCAAAAGAATCCTCGTCTTTTTCCCACGACAGAAACGAATGGTTAACTAGGTTAGAAACTAGAACAGCCACTGGGATTCTGAAACGTGGCTATGCTAACTGTGAGTGGACCTAGAAATTAATAACCTATTATAGTTCTAATGTAATAGCAGTATTATTATAAATTTTCATAAAGGAGAAAGTTGAAAACTTGACTTGATTGAATTGACCCCGTGTCTGTGAAATGGGAATCCACACAACGGACGTAGACTAACCGCAATGAGAAATGGGAAGGATGGCTTGAAAATTTTCGATGGCTATGATGATTGAAAACGAAGCTTATGGTAAAAATCCTATGTACGCTGTTCGACACGTGTATCGGTGTTTTCTTTTCATAATTTTACCTTCACAGGCAGTATAATTGCTTGATGTTGTCTTCACAACAACCACATAGACTCTACTTGGATTTGGCGCAGTAAAAACAATCATATAATTGATATAGTTGGTAAAATATGCAGTGCTGGCTCAATTTCTATTTAAACGGTTCAAATTCAACCACATTTTAAAAAAAATTGCAACTCTCTCTCTTTTATATATAAATATATATAATTATGCTTAAGTTATGTATTTTAATTTATAATATAATAAAATTTTAAAACTGTATTTATTAGTAAATTTTTAAACTCTGACTACTTATTTACAATATATACATGGAGCTCTATTTTACTATTCTTGAGTATCAAATATATAGCAAGACATAGCAAGACAAGATATGAGGGAGAAAAGAAAAGGGTGGTGAATGTGAAAATAGAGGGAGCATGTGGAATGGTTGTGGTCACTATTATATATGTTGTCTCTTACGCTTTGCATGTGACTTTCTTTCTTTCTTTATATCCTTTATATATTAAGTATATGCTGCAATATATTTAATTAAAGAGAGAAGTAACATCAAGGTGCAGAGAGAGGAATTGAAGC

>Glyma.Mads17_3_UTR

TATTAATATATTGCAACTTTGAATGTTCATAGAGTTAAAATATATTGTTCGTGGTCGTGTGTGTATCCATCAAATATATAGTTCCAAGCCTCAAAATTCTAGCTATATAGTACGTTAAGCGTCCATTGATGTAGCTTTGATTAATTTAGTGTATGTTTGATACAAGATATTTAATTTAGAGAATTAATAGTTTTATTTTTATCGATTTCCTAAAAAGCTTTTAAAAAAAATATCGTGTAATTTAAATTGAGTTAATCATGTTATTTATATCTCAATGCATGTCATCTTAATATCTGAAACTAAAAAAAAAAATTCATTTAAGTCCTTAAAATTAACTCAATTTTTTATTTTGGTCTCCAAACTTATATTTTTTTAGAAGAATCTCCAAATTTATGTTAATTATTATTTAAGTCCTTTGACTAGGGACCAAAATGAGAAAAATAAACTAATTTGAGGGATGGAAATGAAATCCTAAAAATAATTAAAAACTTATTTAAATT

>Glyma.Rsp-1_promoter_and_5_UTR

AAATAATATATAAAAATATTACAAAAATCTTATTTATTAGTGATCTCTTTTTATATATAAATAAGAAAAATTTAGGGGGGCATGACTCCCCCCAAGATTAACTAAGCTTCACCACTAATCACAGAGTATATACTACATTCAACTCAAGTATTTATCTATCAGAAATAAAATAAAATAACAATGGTATCGGTTGTTCTGTTGGCACAATTTTTTAGAGAGGCTAATCAAGTTAAAAATACATTAGATATTAGTTTGGAATTTTCAAACTATTCTTTTTCTACTTTTTTTTATCAGAAAAAATAAATAGTTTGAAAACTCCAAACTATTTTATTTATGTATTCTGTTTTTTATTTCTCATGCTTTATGAGCACATCTTGCTTCCATGTATTAGTTTTTCTTGAGGTTTCTCAGCTTTAAATAAATTTTATTTCTAAGCTTTAATAATATTTGAATTAACGAATAAAAATTATTATTTGAATAAAAATTATTATACCTAATAATATATAAAGTCAAATTGTTATAAATTGATACGTCTAAATAATAAATGTATAGTTTCTTACAAAATTCGTGTTTCATATCCACCTAAACCATAAGTCCTATTGGCTCAAATGCAACATATGCCTCATAATGCCATCTCACCCTTCCTCCAAAAGGTCTATATATATCTTTGGTTTCTCTGTGTCTCAATATCACATTCTCATCTCTAACCACTTTGCTTCA

>At.Cdc45_3_UTR

ACCTAGGCATAGTCTCATTGTTCTTCGATTCAGTGTGTTTTCTTTTATAGTTTTCAGTTTTATCTCACTGTTTGCATTTTTTACGAGCCTGTGTAATAGGCACAATCTGTTATCAATCATGTAACTTGTTTAATCAAATAACCATAGAGTTTTATGTGAAAAGGTCCTTTACTCATTTGGTGTTAACTCTTTACCTCTTCAGGTCTTTAACTCTGTTTATATAAAACATCTAATGAACAATCTGTGTTTTGAACGGATAGATTAAAAATACACGCAGGCACAAGACAAGACCAAAGACGATATGGGTTTGGCTAAATCCCCAAAATTTGTACAAAAACATGAACAAACATAATGAAATTCAACAAAGATTAATACTCCAACAATCCAGTTCCCTCTAGTCTAACCCAAACTTGAAAGAGAAAAAAATGGAGACCTGCTCAGACTCGTGAAGCGAAATCTTCATTGAGATGATGGAAACTTGAGAACAAAATGGCTAAAGACATTGTCTATTTCTTAACTTGAAGGCTTCTTTCTCCACAGAGTTGTCCCGCGGTTGATCTCCTGACCATCTCCAGACAACCTCAGGAGGTGCAAGAACTGGCTATCGTTGTGGCCCTGTGTGCCAGACGTGGCAGAGCCATTGGTTCCACCAATTTCAGAGGTGGTGGTGGTGAGTGAATCCACCACATCGTCTTGTTGACATTCTCTTCTATAATCCAGAGTTATGACCTGAAGCTCGTG

>Zm.Cdc45_promoter_and_5_UTR

AGCCACATGCAGTGAATTCTATACTCGATGCGGAAATTCATTTGTGCCCAAGGTGACACTGATAGGGAGGATCATCGCGAGGATATCGCAATAAAAACTTCTTCGGGTTTGAGGTGAATTGAGAGAATTGGAAGGGATCAAATCCCTTCCTATATGAATTTATATAGGAGGCGATTTAATCCCCTCTAATCCCTCTCAATTAATTCACCTCTAATCAGAGATTGGTCTATGTGAGCTTGATAGAGGAGATCTGCATTTGTCTCGAATGTGTGTGGAGAGAGGAGGTTGAGGCTAGCCCCACCATTGAACAGGGCATGCCCGACTTTGACGTTACTGATGATGAAGCTACAAGGGGCAAAATTCGTGTGCCTGCCATGCTGCTAGGACAAGCAGAGGAGTCAAAGGAGATGGAGGTATCGAATTCGTAGGATAGCGGCTTGAGATGGTTTTTACATTGGTTCAGGAAGTCCCCTACGTACAATTTGATCTTAAAATCTTTGTTGCTGATTGGAGGTCTTACCAGGCGGAGCACATATCTCCCGTGCTTGTGTGAAGTACTCGCTACTGTCATGCTTGCTAGTTCCAGTGCCTCGATGCTAAGTTTGCTGGGTTCTATGAGAGGCTTGTTCCCCGTATATAGTCCAACGAGTAGGTAGCTCTGCCCCTCTCCTTTCCGTTGGACAATAGATGTGATATCCTATTCAGGTGGATTGTATGATATTCTGGCCCAAGGTTTAATAGGATTGATATAATACTTATACCAACAATGTCTATCTTCTTTTTTGGAAGCCTATCTTAAAAGAATCTCTGGGTTAAGGATCTTGGTCTAGAGAAGTTTTCCCGGTGCGCACGAGTGAGGGCAAAATACGTATAAAAACTCGTGTTGGTCAGTTAGGATATTTTATGATTATAGAGAGCTACCAGAAATAAGTACTACAGGTGTGAGAGTGGACTAGATGTTACAACAGACACCATGTGCAAAGGTGAAGTAATGATGAAGAAAGAGGTGAGTCCACTTGTCGGTAAGAGATTACGAGAGTTGTATGTGAAGGACTATGGATGGCATATGTGGTTCGCTCAACATTCCCCCCCCCCCCAAAATGTACAATTGCTCAACTAAGCAACTATTGACCTTCAACTTAGGGAGTGTTTGATACTGCTCTGTGTTCCATCTTTATCATGAAGCTGCGGCGTATAATAGAAAATTAAACGGTTTTAGAAAATATTTTTAATCTAAATAAGAAACAAAATGACTTATCTAAATGTCTTCTGAAAGTTTGCAACTTCAGCTTCACGATTTTCTGAAACTCCTCATGACATGTTTCATCAACTTCACTAGATTTTGTGAAGCTGAAGCAATTCCAAACAGATCCTAACAACATATTATTCATATCCCACCAATTACATATTCAACCAAAGGACAAACCAGCACATTCACTTGATCAATTTCTTGGATCAATTCAGAAAGACTAAGATGTTCTAATAAGGCTATCTCTACCACCTTACTCATTCCTCAGCACCTATTCCGAACTCTAGTGTTTTTCATAGTCATATGCACGATCTACTCACCATACCCTAAGACTGTGTCCAGCAATTCACCCATATGGTCATCTAAACTGTTTTGCACTATAAATTTGTTGTCCATAAAACAGAGTTTGAATATGGTTATAGAGATGAGATATCCTATTAAGATAGACCTGGGCCGAGTCGACAAGCCATGGTAGCCGACTTCATAAGTGTGCAGGTCCGCCCACGATCGGAAGGACCCTCGTGCCATGAGAAATCCAAATTTTTTGCTTGGCGATGGCGGCCTTCGACTTCTCTCCCAAATTCAAATTTCAAATCCACCCAAATCCCAATTTCACCGATCCCCGCGCGCCCTCACAGAAAGCTCCCGCCGCCACAACATTCCTCAGATCCCTTGAATTCCTTCAACCTCTCCGAGCTCCGACAATTCCATGATGGCCCTCTCGATCCGACCTAGCTGATTGATGAGGCACGGACCGACC

>Zm.DnaK_intron

GTCTTCGGTACGCGCTCACTCCGCCCTCTGCCTTTGTTACTGCCACGTTTCTCTGAATGCTCTCTTGTGTGGTGATTGCTGAGAGTGGTTTAGCTGGATCTAGAATTACACTCTGAAATCGTGTTCTGCCTGTGCTGATTACTTGCCGTCCTTTGTAGCAGCAAAATATAGGGACATGGTAGTACGAAACGAAGATAGAACCTACACAGCAATACGAGAAATGTGTAATTTGGTGCTTAGCGGTATTTATTTAAGCACATGTTGGTGTTATAGGGCACTTGGATTCAGAAGTTTGCTGTTAATTTAGGCACAGGCTTCATACTACATGGGTCAATAGTATAGGGATTCATATTATAGGCGATACTATAATAATTTGTTCGTCTGCAGAGCTTATTATTTGCCAAAATTAGATATTCCTATTCTGTTTTTGTTTGTGTGCTGTTAAATTGTTAACGCCTGAAGGAATAAATATAAATGACGAAATTTTGATGTTTATCTCTGCTCCTTTATTGTGACCATAAGTCAAGATCAGATGCACTTGTTTTAAATATTGTTGTCTGAAGAAATAAGTACTGACAGTATTTTGATGCATTGATCTGCTTGTTTGTTGTAACAAAATTTAAAAATAAAGAGTTTCCTTTTTGTTGCTCTCCTTACCTCCTGATGGTATCTAGTATCTACCAACTGACACTATATTGCTTCTCTTTACATACGTATCTTGCTCGATGCCTTCTCCCTAGTGTTGACCAGTGTTACTCACATAGTCTTTGCTCATTTCATTGTAATGCAGATACCAAG

>AGRtu.nos_3_UTR

AGCGCTGATCGTTCAAACATTTGGCAATAAAGTTTCTTAAGATTGAATCCTGTTGCCGGTCTTGCGATGATTATCATATAATTTCTGTTGAATTACGTTAAGCATGTAATAATTAACATGTAATGCATGACGTTATTTATGAGATGGGTTTTTATGATTAGAGTCCCGCAATTATACATTTAATACGCGATAGAAAACAAAATATAGCGCGCAAACTAGGATAAATTATCGCGCGCGGTGTCATCTATGTTACTAGATC

>Zm00001d022956_promoter_and_5_UTR

ATTTTTTTATGTCATCCTAATAGATCTAACTGAACTCAAAAGATTCTATATTATCCTTATGCATTCATTTTGCCCTAATTAGCATATTTATCCCACTCTAGAATAACAACCATGAGCTAATCCAAAATTTACTTAGATCTTAACTAGTCTAATCCAGTCATATCCAACCTAATCTAGATTTTATCTAAACTTTGATGATCTAATCAAGAGTAAGTTACTTAATGCGGGTACAAAAGATAAGGCCATACTCCTAGTTCACTCGAGCCTAAATTGGTGACTTAGATCAACTCGGCCATACCCAACAACTTGACCTACATCATGTAATCTATCTCATCCTGATCTATCTTAACCACACTCCCCAACCCTGATGATATACACTAACATCGAATCAATGAGGTCAAGACCGGAGAGGAAAAGATCAACATTAACAAGGAAGGATGTAACAGTCAAAGCGAATCTCGAGATGAATCAAGACTTAGACATTTCAGATGAAGTTTTTCCTATCTTTCTTACCTCAACATGTCCATCTTATATCCTACATAATAAGTAGCTGGATACCTATGCTCTCCTAATAACATACTATTGACTATATAAAAAAATGAGACTCTAGACTTTTAGAAACAACTATAGACTAAAAAAATGAATTACAAACCAATCTTTCTATATCCCTTTTGTTACTAATCTCGGAGACGAGATTTCTGTTAAGGGGGTAGGATTTGCAACACCCTAATATCCCACTGTTGGTATTTGGGGAAAATCTTTCTATAGAATTTAAATAGATAGACCAATAGATACCCCTCTTAAAAATATTATTAAGATATCCCTTTTAATAATAAATTTGAAGATACTCTTAAACAAGAATACCTTTTAATAAAGATTATATATTAGAGATTATATTCTTAAAGTAAATAAGTAACTAATAAATAGATACGGTAAGTTTGATCTCTTGATGATAATTTTGGTGAAGCATTTTATGGAGAACATAACATTGACCTCCAATTCTAAATAAACACATAAAATTGTAAATGAGAAAGGGAATGTTTATGAAACCATTTATTCCTCCCATAAATAGATGGCAAGGAAAAATAATAAATAATAGTAATGTAAACAGTAGAAGTTATATTCACTTGGGAATTTGAAGTTCAAAATGGGAGTTTGAATTTGAAGTGAGAACTTTGAAATAGAAAAGGAATTTTTCTTGCCTTTGAAGTAATTTTTAAACCAATGAAATAATAGATGTTTATGCCTCTAATTTAGATTTGACTTGTGAAATTTTAAACTGAATTTCTTTGGAGCAAAATCTTATCTCAAACTCAATTTATTTAATTATTTGAGTTACATATCTTTTATATGCCTACAAGCATTCATCTAAATGAGAAGTAAAAATGATAAATTGTATTTTAACCGGAGTATACATTTGAGGGGTTATACCTGAGAAATTATTCTTAAATTATATTATTTTGTGTGTATGAATTGTACATTTAAAAGCATTGGCATAAATAAATGTGATGATAAATAAATAACTAACATAAAATTTTATCTTGCATGCCGGAGTTTTATGTTCTTGAGCATTTAATATTAAAATTTAAACTCATTTGAATTTGGAATAGAAATCTAAAAAAACAAAAAATAAAAAAAGCCACTCACCCTGAGCCATGACCGCTCAGCCACCCACGTCCACTTTCCCATGCATGTATAACTCAAGCTGGTAGACTTCCCTGGTTAGCCGCGTTCTTAGCTTAGGTTAGTTGGAAGCTGCCGAGCGAATCACCGTAACCGCACCACGAGCTAAACGCGCGGCTACCATAACCATCCGCCCAATCTGCGGTCTATAGATTTAATGACGGCTGGTTGCATGCGCCCCTAGGTTGTTAGCCATCGCTAATAAATACTGGGCTGCTGTGATTCCTTCGTCACCTCGAATAGCCAGAAACTATCGCCAGCAAAGGGAGAACGCCGAGGTGAGAGAGATACGGACCG

>Zm00001d022956_3_UTR

TTAATTAAAATCACGGTATGAATCCTCGTAGCTGTTTTGCCTCAGTCCATAGGTTGCTTAGCGTGGGTAGGACTGCTGTCTGGATGTGAGGTTGCGTGTCGGTCTGCGCCGACCATGGCGCCGCCGCGAAGCCTGCTGCGCGCCACCGCATTGGATCGACGGGGGAGGAAGAATCGTAGGAGCCGTCCGATCGGTTTTGGGCGATCTGGATTAGATGTGGTGTACCCATTCGATAGGTTAAATCCGAGCCGTTGATTAGATATCAGATCAACTAGATCTGATCGCACCCTAATAAATCTAGATCATAGATCTTTGATCCAACGGCTTGAGTTGCGTACCGGTTCACAGTTATGAATATCTAATCTGATCCGTAGGTAGTCGATCCAACGGCCTAGATATCGCGATACCCCTTCGGCCGCCTATTTTGCATAAGAGCCCCCGGATTTTCCTAAAAACAACCCGCGGTCCAGTACTGGATTCACTGAGTCTGCGTAAACTTTATGCTTTAG

>Zm00001d024378_promoter_intron_and_5_UTR

TGTTTTGATTAATGAGCTCAGTGAAAGTGTTTTTCGACTTTCGGCCCGAGGCCTTCATTTATTCCACGGTTTGAGCTCGTTATGGAAACAAACTAATACCACGAGTGGCTACTATTGGAGGCCCTCTTCGGCCGAAGGTCCTCAAAACATTGTTTTTATAATTATCTCTAAATCTGTTTCATGTAGATAACTTTTATCGAAGGTTACCTTCGGATGGAGATGAGGCACAGTAACAGCGTTTTGCAAAAACGAAGCTAAAAGGCTTCGGCTCAGCGGCACGCATGCATGACGAAGAAGTTAGCAGGAGCAAGGCGGAGTCCTCAAGACTTGTTCAGTAAGAACTATGAAGGGAAAGGACAACAATACCCTTAATCTGCTCCATAGTTCATATGTATGGGTCTAAGGGTATGATTGTAGTTTCCACAAGGTTATACCCCACGACTATAAATAGAGGAACAATGTTATGCATATGGCACGCTTTTTTGCCGGAGAAGAGTGACTCGCTCCCCCTATGAAGTTCTTTTCTCTTTCTACCTACACGTTGTTGTTCATCAAACCTAGGTATAATTGTAATCATTCATCATATAATAAAAGATGGAGAAAAGTAATGTCGAATTAAGATTAGTTATCCATTATATCTTCCATACCGGATTAATTGTATACATGACACTTAATTTTTACAAAATTAGATGGTTATTTCATTTTGATGCATGGAGTGACATGAAAATGAAGTAGTTTCCAATTTTTTTAAAATTTAAGTGGCATGTTTCTAACTGACCCTTAGTATTTTTCATCTTTTGTTCATTCTTTTAACCTTATCCATTTAATTAATCTCTTTGAGAAGGGTTAATTAAAGGGACAAAGTAAAAAAAGATTAATTCATGTGTGTTGTCTTGTTTATGATATTCTTCCGGAGTTAAAAACAAGCGGCAAACCTAAAGTATTGTTGAACACCAAAAGTGGTGGACGGTTCGTGCTCTGGCGATCAGATTAACTGGCGATTATCCTTATCTTGTGCGTGGTTATTCATCTAATCACGTGGGATTTGTTAACTATCATCTAAGAACGGATCCAGACTTCTCCCTGTATATATGAAGGGGTACGGCCGATTGAGAACCCCCGAACACATTCCAATCGAACCAATCTATTTACATTATCTTTTTTTATTATTCTTGTCCTAGGAGTAGATGTAGCCTAGTCTTAGTTGTAGCTTTCTGCATATCCATCTCCACCCCTATTCGACTCTACGTCGTCTAGATCCATCTTAGGTGGCCTGCCGACCCTAGGATCTCGCCCTTCCTGAGGGGCAATATCCACCCTCCTCATCCCTTTAAGTAAAGATCTCTTAACTTGATTCCTTAATTTCTAGGCAAATCTACATCATCTAGAGACGTCCCAGATGATCTGTTGATCCGGAGCGCCCTAAGATCTTTCCCTAGTGGGCGGGATCTAGGACCCCACGAGAAGGAAGACGGCCATGCGCCATCGCGGACCATCTAACCCTGTACGCGGACCGTCCGGAACGACGCAGGGAAGGAGCAGCCCCTGCCGCCAGGTCGCAGACTGTCTGACCCAAAGCCGCAGACAATCTGCGCCACCGCAGAGGGCAGACGGTGACCCTAGTGATTGGCGCTGCCCACGTCAGCGGCAACAAAGTATCATTTAATGTCCCATTTCGGGGTCACTCCCGAAAGTTTAGCTGCACGTGCAGCTCGATCACCAACACATGTAACGTGCACATTGTCTATTCCGGGACACAAGAAACAGAGTGCCTGCGCAATACTCAAGCCGATTAAGCCGCGTTTACGTCGGAACAGCATGGAGGCAGGTCTTCCATGAAGCTAACCTAAGCCAGCTAGCTAACAAGTCAGCCTCCCAAACATTGCCAAGAACAAGACCTGTTCTTAATAGACAACGACAGCAAGAGCAAGGATACCAGAAGCTGATTCTTTGGCCTTGGTCGATTGGATTGCAGAGCCAATTAGCTAGGTAGCAGTGCGGACCGCAGGTAAGTAGTGAGTTCCTCCACGGCACGGCCGCATCTCTGATCGGTTTTCCGGTGGGCGCGGTCACCGCGCGCGGCACGCAGTCACCATTTCTGCACAAATATATATGACATGTTGTTGAATGCATGGACGCGACGCGTGCCGCGTGCGCATCGCATGCACTGCTTGGCCGGTATTGCAGTTTTCAGGTGGTACC

>Zm00001d024378_3_UTR

TTAATTAAAAGCCCTACCAATTAATAGTGTGATTAGACATGGGCATAGTTCATCGAAACAGAGAAATCGATCCGAAATCAAAACCAAAAGAAATGATATACGGAGTACATGAATTATGATTCACTTGTATTGTATAACTATATTTCATTTATGTCTGATGTCTCACATTGATTTGCTATGTATTTATTTACTGTATTACTATTATTCTCTTATCAATTATTGTTATTTAAAATATAGATTGTGTTGTCTAAATTTGTTTTTAAAACCAGCATATTGTTTTATTCTGTTAACATCTCCTGAAAAGGTTCGGTTAATTAGATTAGAACCAATGGCAAATCTAGCACAAAATATGAGTGAAGTCACACTTACAAAAACAAACACTAAAATATCAAAACGAGGACATCTAAGTTAAGCTGTGTGACGCACGCGGCACGATGTTGTCTGGTTTCATGTGCCAAGCCATAGGTGGTGGTGGCCGAAGTAGCTTTGTAGCTGTTGTGTCAGTC

>Zm00001d007419_promoter_intron_and_5_UTR

TTTTTTTTTAATTTTAAAATACGTTTTGCCGAGTGCCAGATCGCGGGCACTCGACAAAGGCGACTTTGCCGAGTGTCACCTGACAGGCACTCGGCAAAGAGTTTTTCTATGTTCTTTGCGAGTGTCAACCGACTGACACTCGGAAAGCTATCTTTACCGAGTGTCAAAATTTAACACTCGGCAAAATACATTTAAATTTTTTAAATTTTGTCTCCCAAACTTTTTGTGGTATGTTCCTACACTATGTAGACCTACATGTATCATTTGTGGACAATTATAACATAGTTTCCATAGTTAGTAGATTTAGTTCGTTTATTTGAATTTCTTCGGAAAATTCAAATTTGAACTGCAGGTCACTCGAAACTTGGAAAACCGTGCATGAAAAAATGATATTCATGTTACTTAGCATAAGTTACGACCGATTGCAGAAGCGTACCGGAAACTTCGAGCAACATGCTCACTAAACATGGCCGTGAACTTGGCATCCACATGTTTAAAAATTGTATAAAACACACACAAAGTCAGAAAATCATGAAACTTGTCCACGTGTCATGATATCATATGTATAGGCTGTGATAAAAATTTTAGAATGTTTGGAGAAAGTTGTGAGACACTATGTGTAGACACATGATTGTCTTTGCCGAGTGCCTACCTGTGCCGAGTGTTTAGCACTCGGCAAAGGGTCTCTTTGCCGAGAGCCTAACTTTACCGAGTGCGACACTCGGTAAAGTCTTCTTTGCCGAGTGCCCGACAAAAGGCACTCGGCAAAGAATACAACACTCGGCGAAGCCTCGGATTCCGGTAGTGTCGTGGCATTGCTTGCCAAACCAATAGGCACTTGAGCTTTGCAGTCGCACACATTGTTGGTCTTCCGGATGTCTAGTACAGCTCATGGAGGTTATTGATTTCTCTAGTACAATCTAAACTTTTATCTAAAAGCAATATTTCATCCTCTCTGTCTTATCACACAATCTTTGGCAGTATATCAACTACCACACATTCTATTATTTATTTTCAACTCTCCTATCCACATCCAACTACCTACCGACGGCACCCGCCTCTGGCCCCCGCCTTTGGCCCCGCCCCCGAGGCTCGCTGCTACCCTATCCAGTTGCACAGAATGGCGCGCTGTATGTAGGGTTATTCCTAATTTCCTAGCCAACCGTAGGTTAGGTCATCTCGAGCGGTCCGTGTACGTGTAGTGTGTATATGGACACGTAAAATATTGTTTCACACTATATACTAAATTGTTTATAAAGTGGACTTTGAAATAGATAGCAGGATAGGGAGGCGGCTGAAGGTATCCTTATAGTGTTATCTTTTCAGTCGACCGGTGCAACGTGATTCCAACCATATTTCCTCACCCTCCCGTGCTCGAGTACGGACAGCCTTAAGCTCACATACTTGAGCAACAATATCTTGGTGCAGATTTCCTTAAGGTAATGCAGGGTCTTCTCGAAACAATGACAGCCTTCTAAATCAATTACAATTAACAATTACTTTTATATACAGACAAGGATAATAAATAGTACTTCCTCCATTCTTTTTTATTTATCGTGTTTTAATTCAAAAAAATAATAGCCGGTGACAATATTCGTAAACGGAGGTAATACTTTATAACGTAAAGATAAATAATAAATAAAAAGATAACAATTTAATTATACTAGGTGATTATATAAGAATATTGTCTTTACTTATGCATCTAAAATATTGGAGAAGTGCATTATACATGTGTTATTTTGTAGCTGTGTGATGCGCGTGCTATGTTTAGGCGTGGTGATTCCCCTCCTCCGATCCGATCCATTCTTTGCACCCCAATAACAACCAGGATGCGCTGTAGGCTGTAGCTTCTCCTTCCTATGGATAGCATAATGATGTCCGAAACGGGAAAACGACGAGAGCTTCCACCTCCCTCCTCCCCGTCCCGGCCGCTCTCTGCAGGGCAGGCCCATGCGGGTGAGGTACCGGTGACGACGCGGCGGCCCACGGCCGGAGACGCGGACCGCAGGTAAGTAGTACGTACGTTAATTAAATGATGATATGGGTATGATTAGAAGCTAGCTACCGCGATCGTTGCTAGCTAGTAGTAACTAGCAATTGCGATCGAGGATTCCCTCGTGGCCGATCCTGATGTGTGGTTGTCTGAGACGCGCGTGGTGCTGGTGTGGCGGCAGTTTGCAGGTGGTACC

>Zm00001d007419_3_UTR

TTAATTAATGATCGAGCGCATGCACGCAGGGGAACGAGGGGACGGGTCGAGAAGAAGCAGATGCTGCCGTGTTCTCAGTCTCTCAGCTCGATCGAGAATGTGATTTGTGCGTGTGCAATTTTGCGTGATGTGTCGTACGTAAGTGTAAGTCGTAGCATCAGCTTCGTTCTCCTCGTATATGTCTGGAAATGGGATGCCATTCTACTTCCGGTGCATGGTACCCTGTAGTTCAAGGATTCATGTATATATATAGCAGTATTATACACATATGCATATACATAGCAGGCTGCCTATGCACATGAATTGGTGTCTAGTCCCATGTTGGTATACATATATAATATGGTTCTATAGTTAGATTTTGTGGGATTATTAGTCCAATAGAGAAAATCAATTAAATCCTAGAAAATCTCAAAGCCTCGTGTATGACAAGGGGATGGTGGAAACAATAATCCCACATTACTAATTTAAGTGGACCTAGCTAGACTAGTTTATAGGTCACCTGATCG

>Zm00001d017180_promoter

CCTTCCTTCCATATTTACTTCTTATTTCAACCAAACCAAGATGTTAGGTTTTAGGCCCAGAACCATGCATATGACTGATTGTAAGCTGTAGGCACAGATGCCAATGTGTTTTTTTTTTCTGTTTTCAACTTTTAATGACTACATCTCTGCTGAGGGTGGCGGGTGATGAGCAACCTGTCTCTTTATTTATAACAAACTCATACCAAGAATGACAGGTGCTTTTGCTTCTATATTTGTTGATCTACTATATTTGCTTCTATATTGGGAGTTCAATATAATGATTAAGTAATATAAGTCTCGGGATATATTTTAGTTGATCTACTATATTTGTTGTCTGATTGGAATCCTAATACTTTTGTTAAATTATTTTTGTTGACCTAATACTTTCAAGTAACTGCAGATTACTTCTGTGAAAATTATACGGAACAAGCAGACTGGGCACTCAGAAGGCTATGGTTTTATTGAGTTTTCCTCTCGAGCTACTGCAGAACATACTCTGATAAACTTCAATCGGCAGATGATGTCGAATGTTGAGATGACTTTTAAGCTGAACTGGGCTTCTGCTAGCACTGGTGATAAGCGTGGAGATAGTGGTTCTGATCACACAATATTTGTTGGTGATTTGGCTCATGGTGTTACTGACTCCATGTTGGAAGATGTGTTCAGAGCTAAGTACCCTTCAGTTAGAGGAGCTAATGTTGTTGTTGATACTTGATAGGATGACTGGATGGCCCAAAGGATATGGTTTCGTGCGTTTTGGAGATCTGAATAATGCATTGTGAAGATGTACATTTGTGCTTGTTTGATCTGAAAAGGGGACAACTTTTAGATATAGCATAATAGGATATGAAGATGAAAGATCTGTACTTAGAAATTTGTTCATGAATGCTTTGTTTGACATTTAACTTCGTATGCTGGCATCTCAGTGTATTAAGTGTTAATGTTACAATTATTCTTGGATCAACACCATTCGAGTATTTGACTATTGTTTGTCACGTGCACTTATTATTCTTGCATCGCTATTTTGAACCAATTTTTAAGTTACCGTAGCAACGCAAAACTTGATCTGAGTGACTGATCGTGAGCTAATGTAACAGTATGAATTATATGGCTTGAACATGGTAGGTGTAAAATGTTTTGTTCATCGGTAGAGTATTCCAGATGTGATCGGCTTAACAGTATGAAATATATAGAACTATGTGAGCCAATGTAACAGTATGAATTATATAAGTTATCAAGATATTATATGCCCTCGTTGCAACGCACGGGCACTGACTTATTATGATAATATTTGTTGTTCTGTATCTATGTTTCGTACTAATTTTTTACTCCCGTGGCAACGCACGGGCACGAACCTAGTAGAAAGTGATAGGAGCAAAATGGAAAGCGAGGAGTGTAAATGAACTCAGATTCTAGGATTTTTTAATCGATTGAATTTTTTTATTTCAAATAATTAAAAATACATTTGTCTGACGTGATGCTGTGCATATGGATCAATCAGAACATGTGACATCGTAAAAGCAGTCGGGCTAAGGTTCAAAATCGGTACTGACACGTCACGTCAGCTGTATCGAGCGAAGGATAATATCCGACATCAGTTCTAACATGATTGGTACTACCAACTAGACTGATCGATCGACGGACCTACCGATCGGTGTTGGAGTCGAAACGATCGGTACCGACCATTGACTCGCCTAGTCATAGCCCATTGTTAGAACAAGACTTAGACAAATACACGGTAGATCATCTCTCCAATTTTTTTCAAGAAGAAAAAAAAATAGAATTTGAGATAACTAGTCCCGACCTCCCAAGGGAACATTTTTAACAGGACGCCTGCTTCCTCCCTTTTGCCCTCGGCAGCCATAACTCATCCGGGACCGTCCTCTCCCTCCCTCCGTCTCGCCCTATATAAAGGCAGACTAGCTAGCTCTTTCTTCTCCACCGTCGTTCTTGCCACACACCTGACCGACCTTGCCTCCTCTCCCTCCTATGCCACCAAACGGACCGTCTCAG

>GSI85_computationally_derived_intron_and_5_UTR

GTAAGCTACTTCGATCCGCCTCACGGATCCGCTCGGGTTTCTCCCTTCTCGATTGGTGGTTCGTTGTTGCTCTACTCTACTGCTCGCGTGCCTGGATTCGGTAGATCCGCCGCGCTGTTGCGCTGCTCCGTGCTCGGATTCGTTCGTATCTTTTTTACACTGCGCGCCGCCGCCCGGCTCATTATTATATGCAGCAGCTCTCTCCCTCGATCTGAGCGGAGCAATATTTGAACGGATCTGCCCGCGATATCTGATCCCGCCTTTGCTTTGTTTCTAATTGGTTTGGGGGATGATTACAG GTCGAGGTACCGCGATCGCACC

>Zm00001d017180_3_UTR

GCGATCGCTTAATTAACGGCGCTATCCTTACCGAGCTTTCATGCATGTACCACGCCCTCTTTCCTCTTCGCAGTCTTTGTTTCATCACTCAGCAGAATTCACGATGTATGTCTTCTTGACCTTGCAAGACTTTTCGCTAGAGGATGAATCCCAACTTCTTGTGCCTTTCCATCTCGCACCTATCAATCCTCCCATCTTCTTCGTCTCCGTCTATCCCTCTTGAGCTTGTACCGCTTTGCAGTTATAGCTAGCTAGCTAGCTACGCCTACTTCCGCGTGGACTCCGCTCTCTGGAAGTAAGTGTCTCCTCGTCGCGTGCTGCCGTGCATTGTGTTGCCGGCCGGCAGCTACTTCCGCACGAACTCTACTAGTACGCTATTATTCTGTGTAATGGAAGGTTTGTGATGTACTCCCTGTTTATTTGCTAGTTGTCCATCGCCTTTCAGCGGAGGCAGCAATAGCTTGAGCTTTGCTACTCGTGTAATTATACGTCTCGGAAATGCAGTTATTAGTTGTTCCCGATGTGTTGTGTTGTGTTGTGTCTCATTCAGGCACTCTGTGCCATGCAGTTGCTTGATCTGTGTGTGTATGCCTGTTCTCGCTAGTCATGTCTTCTTGTCCCAGGTGAGGCCTGGCCTGAGGAAGAAGGGGGGTAGTATTCAACCGGTTGAAACACAAGTCAGCCCAGCGCCACTGAAGTACTGAACTGAGGCTAACTTAT

>Zm00001d018050_promoter_intron_and_5_UTR

CCGCGCTCTGTATCGGACGGTCTGCGATGACACGAGGTCGTCTTCCCCCTTGCTGGAATCTAGATCTCGCCACTAGGGAGAGATCTTATGGTGCTTTGGGCCGATAGGTCATTCGAGAGGCGTCCTCAGATAATGTGGAGTTGCCTAAAAATTAAGAGACAATCGAGGTAATGTATCTTGAATGGACAACTAGATTTTGTCTCAGGGAGGGATAAGATCATATGGTCGTCTTGGAGTTGGCAAACCAACCAAGACAGATCTAGACGACGTAGAGGGTGGAGGTGGATATGCATAAAGCTACAAGTAGAACTACACTATATCTACTTCTAAGACATGAATGGTAAATAAAATAATTGGTTCGATTTAATATTGAAATGTGTTCGGTGGTTTCTCAATTGGTCGTACCCTTCATATATATATGGGAGGTTTGGACCCATTCCTATGAGATAGCTAACAAATCTCACATGATTAAATGGATAACCACGGACGAGATAAGAATAATCACCCGAGTTAATCTGATCGTACGCCTTAGGGCCGAACCGTCCGCCACTTTTTGTGTTCAACACTGTAGCTTTAATCCGACTGCGGCTTCTACGATATTTTGTCTTTATATATTATAGGTGTAGCAGTCCAAATAGCTGCAAGCCTGCAACCAGGCCCGGGCCAGAGGGCGTGCGAGGTGTGCGATGGAACAGGGCCCCTAAATCTTTGGGGCCCAAATGTGTAGGTTTATAGTACATTTATAAAATAGAAATACAATATTAATCCCTATTTTTTTACTTATCTGTCTGTACTTATGCTCAGTAACGCAAGTAATAGTGAAGTAGACGGGGCTCCAGCCCCTACTCCACTAAAGACTCCTCTAAAATTTAAAATGTATATATATGATATAATGCAAAAAAGATCTTTTACGTTAGCAGTTTCATTTAGTCCTCTCATTCGTTTTGGCTCCGTCTCTGCACATGCTTCTATTATATGACTGTGAGACGTGAGTTTAGCCATAACGTGTATATCTGTAGTTTTACGACGTTTCATTCACCGGCGACGGGTGCCCTCCACCAATAAAAAAGGCCTCGTTTTAAAACTTGTTCAGGGCCTCAGATTTTGCCGGCACGGCCCTGCCTGCAACAATGCAAATGTTGTAGGGCATCAGCCTATTTATATGTAGTAATAGTAATAACCACATAACTTTATCTGCTAACTAAAATTCTTAGAATAACAAAACAAATGCTGTCGGGGTTCACCAATTCTATGTGAGGAGTTCGCCAAACCAAATACGGCTGCTGGGCTGCGTTGGCAGCCGCAGGATCACGGCCCCTTGAAGCGTCCGCCGCTCTGAGCCGTTGGCAAGCAGCCTCGAGAAAAGCTCCCGTTCCTCTCCCTGCGGTCTCGCCCATGTACAACGTTCCATCCAGTCCGTCCGCCGTCAGGCCCGTCACGAGACCGGACGGCGCAGATCGACCAGGCGAACCGGAAGCCGCATCCGCACCGTGCACAAGTCACTCCGTCGTGTGGGGCCTCCGCACAGCCCCATGGCACGGGCCGCCTGCCGCCCGTCCCTCCTACAGGCTGGCACGCATCACGAGGGGCGTCGTGCACGAAGGACGCTTAATCACACAAAAATTATAAACACCCCTTTAATTACCTATTCCCTTACGTCCCCAGCAGCAGTAGCCCCAGCCCCCAGTGCCGCAGTTGCGCTCAGCTATCTCTGCTGAGCCGATTAGATAAATCCGGCGGAGGAAGAGGGAAGAATTATCGTGCGATATTTCCCCCCTCCCACTCCCTCTCTCTCTCTCTCGATTTGCCCGAGAAATTTTTTTGGTTGTTTCCCGCATCACTCTCCACTAGGCCGCCGTCTCCCCCGCTGGTGCTCGTCGCTTCCTGCCCAATTCGGCGCCATTCGCCCGGCGCACTCCCTATTAATTCCCTGCCTCGGTAGTCACTGGCCGATTGCCACGTGAAATTTCCTGTTCCGTTGCCTTTCGGGTGCGCCTGAAGTACCCACACGCTACTAGGGTTCAATCGTCGTCAGCAGGCTTACCTCTCAATCACTGCGGGGCGGCGGCTCGAGGTACTGGTTCTTCGGTTTCTCCGATTCTGGATGCTGGATGGATGGATTATAGGCGGCTTTGTGGTTTGTTATTCCTTCTTCGGATGGATCTAACTATGCGTTTTTTATTGTACAAATGTTTGTCCATCCCCTCTCTTGCAGAGTTTG

>Zm00001d018050_3_UTR

CTTCCAAGTTCAATGCAGTGATTGTCGATCGAGCAGATTTGGCCAGACGTATATGCAGCTTAGTTGGTACATATACAGATAGGAGAACCGGTTGCAGTTTGTCAATATTCCTGCTTTACATGACTTGGGATTACAGTTAAATCGACCCGCTAGCAAAAAAAAGTGTGCAAATGTTAGTAGATTTGGCCAGACATATATGAAGATTTTGGGGTTGTATCATAAGGCCTCGTAGGATAGATCTTTCTGCAAATGTTAGCGAAGCCAGTGTTTATGTTCTGTTTGCTAGATTAGCGTTATCAGTTTGCACGTTCCCCTTTTTTTTCTGAATAATTGTCCTTGTATGATCTTGAGGGAATGCGTTTACCTGGTCGTTTGCCATTCTTTTTACCTTTGTGCACTGTGGTGCTCGTCGTGGGCCCAGGATATGGAGTGCAGCGACGCCGTGACCTCGACCTGAATTTCACGAGTGTAAACCAGGTGTTGGCCGCAGCGTCGGAAGAGACCAAGACTTTTCCATGGTTCCAATTCACAACAACTTGCAACCAGAACTACGGAACTAGCGATTTTACCTGGTCGTTGGTTGATCTCTCCAGCTACCATGTGAATCTATTTACGACTTGAGGAGTTTCTGCAATCTAATCAAAGTTCAGGTGCTACCGTGCTCCCACACACCACAAAAAACACACCGATAATCACACGGAAAATTACAACCGAAATACTGTGAACAGCGGAGAGAGGAGGAATAAGAACAAAGATGCAACATGGAGGGCAAGAAAGGACTCGGGAATATAGGAGACTGATTAATGTTTTCATATTTCGGATATTCTTTCATTCCCTCTACTTAGCACGCAGGTTGGCGAGGCAGAGAAGAAACATGCAAGCAGGCACCACTGGACTGCACTAGCTAGTCCATTCTTTACCGGTCCACCGTTGGTTGGGCTTCCTGGCCACGCATCGCGGAAGGCTATCCACTTGGGTAATCCAATAGGAGAGGTAAGCTTTTTAAT

>Zm.BA1_promoter_intron_and_5_UTR

ATGAACAAACGATATATCACAAACCCTAGCAAGATATTATTAGTAACAGACATGTTTGTATTTGTCAATTAAAGATGCAAATGTGTTATCGAGTGTGAATAGATGATGTTTATAAACTTCCTTTACAGGAATTTCAAAAAGGAAATCCAGATAACTTGATCAAAGGTGCATTGTATGGTATAGTTGAAGACCTATGCACATTCATATTAAAAGAGGTCATTCTAGCAGAAGAAAAATATCACAACACTTCCAGTACATTAGCTATGCCAGAGTTTAGAATTTTAACAGAAGTTAGTAGACTATCTCTCAAATGAGATAGTTACTAGCTAGAGCATAGGTGAAAACTTATATGTTCAGTTTTGATGTATAGAATGTATCGAATTTAATTCAATTTTGATTGATTGAAAATTTATGGTTGTAAACAGAAAAATATATATTGTAAACAATATATTATAAACAAATTTGTGTTAATAAATATAAAATTCAGTTTTGTTTGAAAATTTGAAAAAAGGCAGGAAAACTTATACTGGCGGAAAATGGTAAGCAGACCGTCGGTGCTAATGTTATTTCCACTGGTGAGCTGCTAAAAAACCGCCTGTGAAATCATGTTTCCACACTCGGTTTCTTAAGCACACCGCTAGTGCTAATATTATTTACACTAGCGAGCTGCTAAAGAAAACCGCCAGTGATAATCATGTTTCCACATGCGGTTTCTTAAGCAAACCGTCAGTGCTAATGTTATTTCCATTGGCGGTTTCTTAAGCAGCCCGCCAGTGCAAATCATGTTTTCACATGCGGTTTCTTAAGCGAACCGCCAGTGCTAATGTTATTTCCACTAGTGGGCCGCTAAAGAAAACCGTCAGTGATAATCATGTTTCCACATGCGGTTTCTTAAGCAAACTGCCAGTGTTAATGTTATTTCCACTGGCGGGATGCTAAAGAAAACCGGCAGTGCTAATGATATTTTCACTGGCGATTGGCAAACAACCGCAAGTGAAAAAGGCCGATTTCTACTAGCCCCTAGCACTGGCGGCACTGGAAAAACGCGAGTGCAAATAGCTCTAGGACCGCCACTATAGAACTTTTGTGTACTAGTGACAGGCACGAGTAGTAACACAGTACTCAAGCAATACAACAATCAACACTACACTAAACCAGGGCGAAAGAGTGGACACGAGTATAACTCTCTGTCTCAAACCATCTGCTCGAATTCCAAACGATTCATCATTCGGAGTGAGTCTATGTGGCATCCCTTCACGAACACAAATTCTATTGTCCACTGATTATCTGAAACCATGATACTGATCTTTTTTATTTTTGGTTGTCGGAGTAATTTATGTCCTTTTTAGTCAGGTACAATCTTTCTTTCATATCAAAACCAGTTCTTTCCTTGATTCTTTCTTTTAATGCATGCCTTTTTTTGAACTTTTGAAATGATAGAATTGACTTTTCAGTGACCACTATTTAAATTTCTCCTTTTAAGCTAAGCTACTGTAAGCGGGATGGACAATAGTGATCTTCAAACCCAGTACTATATATATACGATCGCATAGCCTCATTGAAAAAAAATCTTTAATGCGTGTTAGCATTGATCTCTTTGATCAAATGGTTGTGCATTTTCTCACCAAATTTATTTTATACTTCATCTGTTCTAAATTATGTAGATATAGTGTACGTCTAAATATATAGCAAAATCAACATATGAAAATATTAGAATGGCTTATAATTTAAATCAGAGAGAATATGTCACTGCTTTTCGAAGAGCATGCAGAGTGGAAATCCCATGAATTTCACCAATTTGATACAGCGAAAATGCAATTCAGATTGTTCTCTGCATGTGCTTAACTGCTCATACACGCTTCCATGCAATGCCATACGCTGAGTATATATACCCGTCATGGGCTCCTCCCAAACCCATAGCTCTAGGGCTGAAACCAGCAAAGCCAACAGAACTGCACAGTGTAGTAGTTGCACATAGGCGTCCGCGCGTCGTCCTAGCTCGGACCGACC

>Zm.BA1_3_UTR

AGTAGTAGTAGAAGTTTCTTAAGGTAGCATCCCGTGTGTGTTGGTGTCTGCTAGACGCTAGTACGTCTAATTAGCAAAGTTTAGCTAGTACTCGATCAATTGTCTGTCTAGTTCGCTCAGAGTTAAAGTATATGATGATGCATCTGCATATATGGGCTCTGTAATTCTGTTATCCGCTGATCGCAGATGATACACCGTATGTAATCACATGTATGTATGTTGCCTAGCTACTTCCTCTCGTGTCGATCAAGAATCTGATCTAGCTAGATGGTAAAACTATAGTGCTGCAAACTTTCTGTTTTTCTCATTAATTACCCTCTCGGTCTCAAAAGTAATTTAATTCTATAATTGAACAAAATCACCTTGTAGTATGTCGTATGAAAGACTAAAATGACTCATTTTAAATGCAGTAGTATTAAGAAGGGGGTGTATCTCCAAAGCTAGAAATTAAAACAAATAGCTTCGCAGTTGGCTTTTAAAAAGTTGGGTTGTGAGAAAAA

>Zm.Mtf32_promoter_and_5_UTR

GTTGCAACCAGGGAACGACAAGCCAAACGCCGGCGGCCACACCCACGCACCACAAAGACAAGGCTACCCAAGACTGCCTCCAGGCTCCAACGGCACCCCCGCCCCCTCCACCTAGCGGCTATGGCCTATGGGTGCCCGGCCACGCCCCCTCGCCGTCCAACGGCGTTCCCTCCCTCTCCCTGCATGTAAGGGGGTTAGCCAATTTCCCCTCGAGAGTGTAGCTCTAATCTCTCCTTCTCGGCTAATTGATTATATTTAATGTAAAATTAATCTACTTTGAGTTTAATTAACAGATAATACTGTGTATTATGATAATGTAAATATAGTGGTAATTTTTAAAACTTTAAAAACTGAGGTATAAAAGTCAAATAGTTTAGTTAAAGAATAAAGAGGAGCTGAACAGGGGATGATTAGAAAAGAGAAGAAATAGGGGAAGAATGGTTAAGGAGAAGGTATTTAAATATGAACATAAAGTATGAATATGGGATTTAGAGGGGGAATGAATGAATGGTTGGAGATAGCCTAAGGCTGAAGAGAAAATCCATTCAATGTTATTCAAAACTATAACAATCCCTTGGATGCCATTGAAAAATACATGTTCCCTTCAATGCCACCAATTTAAAATTTTAATCACTTATATGCCATTGCCATCTAAAATCAGTTCAAATTTTGTTAGTTGGTGTTGGAAAAGAATAATGTACCCATGTGTTCTATACACTAACCCATCCTCCTAAGCCCTTTTACCAGCTTAGCTTTGAAAACTACGTAACATCGATATATATATGTAATAGTAATATGACAACAATATTTTATAAAAAATAGTATTATTTATAAAATGGTGTTGTCATATTATTGTTACATTCTTATATTGATACTTAAATTAATGTTCTGGAATTCTCAAATATAAGTGGCTAAAAGGACATGGAAGGGAGGGGTCCATGTACCTAACACATGGGTAAATTAGTACCTTCAAACTAACATCTCTTTAAACGGTTTTGAACGGCAATGGCATCTAAGGGACTAAAAATTAAAAGTAATGGCATTGGAGGGAACATGTATTTATCGATGGTATTGAAGGAATTGTTATAGTTTTAGATGGCATTGAAGGTTTTTTCTCAAGGCTGATTGAGCGTGAGCCTGAAGTAGCTAGACTACTACCATAAGAATTCACGAATAGAACATTGGAAACCCGTTAAAAAGTCGTATAGTATTTGCAAGGCACAAAAGGCCTTGTGTTAATATGTACAAAAGTCGAATAGAACTTTGATTCGACGAGAAACAACTTGGCAGTATGCTTGCTTCTTTTGTTCATATAATTTTCTTCTTTAAACTTGTATGGTGTTAATGTATACGAAATTTGAATTCTATGCAATAATAGTTTTTTAAATGTTTTGCTTGGTTTTGTAATGTGATTTTTTGGCCCCTATGAATTTGGTCCTGCGTCTGCTACTGACCGAGACTCACTTCACCTCATAGGCTCATATGTTGATAGACGACGTAACCATCTTATTTTTTATTTCTAACTATAATGGATCTTGTTCTACACGCTTAAGGCTAGTTTAGAAACTTAATCCCTAGAATTAAAAAGGACTAAGGGAGAAATTAGTTTTTTTCTCAAAATCCCTCCAATTTTTAAGGGGTTTGAGTTTCGAAACTAGTTCGGAAACTTATTCCAGTTTAGAAGCCACAAAATAGAGAGGATTGAACGAGCTAAAATCATTTTTCATATTTAGGCTCCCATACTAACCCATAAATAAAAAAAATACCGCTACTGCTAATCAACTCGACGCGGACTACAGTCTCTCTCCATCTTCTCCCCCATTGCCGGAAGCTCAACAAAAACAGGCAATTGGGCAACGCATAGGCATAGCTTGGCTCGCACGGTGTCGCTGGCTCCCCTCCCCTTCCCTCCCCACTGTTACATCCAGCGAGCTGTTCACGGCCGTGCCGAGCTCAGTGAGGCAGCGTTTGCGAGTCCGGGACGAGGGGAGCGGCGGGCGACCGGACCGACC

>Zm.Mtf32_3_UTR

CGCTGGCTGGCTGGAAAGCTCGAGGACTATGCTCTGTTCCTTGGGTACTGTACTCTGTAGGTTTTGGTTGAGTGGTTGTTGTCAGCAGTTGTCCTTAGATCAAACAAGTCTCGAGTTTGATGTGTTAATGTACTCCATTATTCAGGTGGGATCAAGTACCTTTGCTGCTCCGATTGAAGTATATTGTTTTTGTTGGCCGCAGAAAAAAAAGGGCTACTTTTGACAACTCCATTTTTACAGAGATTCCTATTTTCATAAGAGAAAATGAAAAAAAATAAAATCCTTTGGAAAAAAAATAAAATGGGGTTACCAAAAGCCCGAGGGAGACAGCCCCCGGAATATGGCAGGCAGATCCCCGTTTCTGAATCCAGAGTGTTTCGGTAACAGTAACACGCCAAAAACAGACTAAAGCCTGAATCCAAGTAGAACTAAAAATGGGATATGTGCGCATTGTGGCAGGAGCTCAGCAAGAACGTAGCAATATAGAGATCGATAATCAC

>Zm00001d043589_promoter_and_5_UTR

TAGTTACTACTAGATCGCAGAACTAAAATAAAATAAACAGATGGCTTGGTGGTAATGTCATATTAGCAAAACCGCTCATTAAAACTGGTCAGGGCTTATTTACCCGGTTTTGTGAAGTTGACAAAGTTAAAACAAGTAATTTTGAAAGTGAAACTATGCAGTAGACTATGCTCTACAAGGGTTACCAAATCGGTAGGCTAATCCGGCTAACCGGTACTCGCGTCGGTCGTTTAGTTCGGACCACCGGTTTCAAATTTTCAATTTGTGAAATTTTGTAAAATTTGAAAAAAATAGTGACAATTTTATTATAAATACTATGGATTATTTTAAGACTTTCTATTGAAACTAAATTTAAAATATATGATACCAAATTTAAACTAAAAATCAAACAAGTTTTAAAATATTAAAAAAAACTCCTAAGTGGGTCGGTCGTCCATAAGGCCCAATAGCTTCATATAATACCCTAACATGTCGTGACCTTCGTCAGGCCATTCCTCATCCTCCTCCCCGTCGCGTCCAGCCAAGAACAGCGCCCTTCCACCGCCCGAGTACGCGGTGTAGCGCTCCTACATCCACTAGCCCGGAAAGCCGTCGGTTAGTACTTCTCATCCGCTCAGTACGTAAGCCGCTCATTTTACCTATTAATTGCCAATTATATACGATAAATTAGTTGGCTAGTCAACGGCTAAATAGGTGATGTAGCCTGGACGGGCCATCGTTAGTCGCCGGCTAATCGGTCTGCTTTGTCCTTTAGCCGAGCGTGTTGGCCGACTAATCGGCCAGAGATGGTGATAAGCCAACCTGCTGAGTAGAGCTGTAGAGGATAGGGTTAGGGAGATTTACCCCTTTTGGCTTCAAATCCTTTGAAATCATCACTGCTACTTGTTATGTAGGCTAATAATACTTGTAATAATAGTTGTTGTTGCTATTTCTAGGGTTGTTAGATCGGATTGATCCAATATGGGAACACGGGGACAATATATCTCCGATTCAGGTGCAAGTACTGCAAGAAACAGTGGAAGGGGGTGGTGCTACACGTTTCAAGCAACACAATGCATCGCTTGGGAGGAATGTGCATGGTTGTCGTAATGTTTCGCTAGACATTGCCTTTACTTTCGGCGTGAGATTGACAAGGTCTATGATAAAAGGAGAGCAAGAGTAGAGTGGTGGGTTCGCACCGTGTTTCCGATGATGCCGATGCTGGTGTTGATGCTGAATTACAGGTCGCAGGCCGCATTTGCAGGATTATTTCACAAAGTTCAAGGATGATTGGCCAACATATGAACCTACCATCATGTGCGACTCATGGACAGGACCAACCCAAAAGTGAGCGATTAATTTCATGTTATTTTGCAATGGTGTTATGTTCTTCCACAAATCAATTGATGCAACTGGTAAATTTCAAGATGCGAACTATTTACACAAGGTAATTTCATTAATCGTTTTATGTCTGTATTTGTATCTTCGATGTTGTTTGTGAACTATAAAACAATGCCATGTAATGTATTTGAACTGTTAAATATTGTTATTGGACTTTTGTTCAAATTATGGGACTTCAATGTGATTTATTTGCAAAAAAACATATACCTGAGTTTCTGCATCAGTGCCGCCAGTTTATCTGGTCAGGAGCCGATTAATCGGTCTTATCGGTTGACTAATCGGTTTTAAATTGTTTAGAATTCAAACTAGGTTGACTTTAATTTCTATTGGTTTTCGTCGATTTATCGTGGTTTACAGATTATCGATGGACACCGGTTTGTGTGTCCTCAATCCGGTTTGGTGAACTCTGATCCTCTAAAGATTTTACAGATTTCCGTAGCAGTACTTGTTGCTTGAATCCACATTTTCTTCAAAGGTACGTTTAGACTGTGTCATTTAAAAAATTGTTTTGTCCTGTTTAACAATATAAATTGCAACATTTATAAAGATAACCTTATAACAGAGGAAGAGCAATGGAAATCACATGGGTCACGTCTATCTACAATCTACTAGACTACTACTGTACGCCTGTATGGCTGTATCGTACACCCTAGGTGCGTGTGCGTACTTGTTGCAGTAATACACAAGTCACCACTATATCACTCTCTACAAGCCCACTACTGCTCCTGCCACTCCTACTATACAGTATACACTGCCCAGTTGCGCTAGTGTAGCGTAGGAATACTGTAGGCGATGATCGACGGTCCACTGTGCCCAGCCCTCGACAAGTCTCAGTCGCTTCATGCACGTCGCGGTGCCAGTCTCGTTGCTCCTGCTCCCTTATCCTGCAAAGCTGAAAGCTGCAAGGCCTGCAGATCACTTCCAGTCTTCAAAAGTACAGTTCGCGAAAGTTGAAACGAGCGAGCGACGAACCGAACCAACCAACCGGCGGTGGCCACGTAGCCCCCCCTGACACAAAGACAAAGCTACCTAGAAGCTAGCCCGTGGAGCGGCTAGACTACTAGACTATCCGGACCGACC

>Zm00001d043589_3_UTR

CGCTGGCTGGCTTGGAAGCTCGAGGATGGATCGAAGACTATGAGCTCCTTCATTGTGCCCTGTCAGGCTACCTACCCCTGCACCCTGTTGGTTATGGTTTAAAATGGTTATTGACCAAAGTTGTCCTGCATCAAACAAGGCTCAGCTTGATGTTAATGTGCTGAGGGATCTAATGTATTATTAGCGTTATCCAGCTCTTGTTTACGTGCTCCATCTCAATCCATGCACGTTAGGATGGATTGTCTAATCTAGTTCAAATTTCGTATCAATCTATTCCATCCCATACAAATTAATCCATGTGGATTGGGTGGTATTGAGTCGGTTTAATTCTATAGCAAGTCAAAATCCATCCCAATCCATCCCAATACACACCAATACACATGGAATTGAAATAACCGAACCAGGCCTAAGATGGATATCTGAACCCTAAACCCTAAACACATGTATTCATATTAAAATTAAGGTGCATACATGACCTTAGCACATATATTCAAACTGTG

>Zm.Ra3_promoter_and_5_UTR

CAGCCGTGGTATAACCTTACCTAGGAGCTAACGGTAAGATAACATATCTACTAATGATTCAATATTATATGTGGTTCTATGAGGTGGACCAATGAAATGGCACACACCATCTGTGTCAGCTTCAATTATATATTAACAATCAATTTTGCTATTTGATATGATATTTCTTTAATATTTTTATACTTAAATCTCAACCACGAAGTTTTTAATTGTATTTAACTAAAATCAGTCAGTAATCTTTCGATGATCAAAGGTAACCAACTAAGAAGGTTACTTGCAAGGAACCATGACACTTGTAATGGTGGTTGTAAATACAGATACTACATCATCATCTGCCCATAAGGTATTCTAGAATACTTAGCGCAGTTTACATGCAACATGTGAGTACATATGGTGGTTATGTAGGAGACACAACTGGTTGCTACATGGCAACTTCAACAATTTGTTCAGTTATTCATGCATCAAAAACTATTGTCATTCCATCACCGCAATTTGGTTGGCCTAGGTAGTTATTCAGAGCTTCTAGTATTCTCACTTTAAAATCATCAATGAACACATACATATAGCTTGTTGTACTCATAAAACTATAGCGTAAAGCAATATATCTACTGCTCATTCAAGAATATATGTGGTTTTATAAAGATGGACCAATTAAAGGTGCACTCAATCTATGTCATCTATGGTGAAAAGGTGAAACTTCTATATATATTAACAACCAATTTTGCTACATGATACGATATCTGTAATAATAATTTTATACTTAATTATGCACCGCTATATTTTTAAATTATATCTTTATCTAAAACCGAAAGCTTTTCCGTGATTAATGGTAACCGGTATAGAAGGTTGCTTAAAAGGAACCACAACACTTGGCATGGCAGTAGTAAATATAGGCACTATACCATTGTCTGGCCACAAAGGTACTTTAGCACACTCCGCATATCTTTCGTACAATGTGTTGATACATATGGTGGATATCTAGAGGACATTGCTTGTTGTTACATCACAACTTCAACAATTATAGTCATTCATGCATCAGAAATCACTGGCATTCCTTCACCACAATTTGTTTGGCCCAAGTAACTTATTAATAGCTTTTGGTATCCCCCCTCTCTAAAATCACTAATGATAACATACACATAGCTTGATTTACTCATGAAACTATAGCATAAAGTAATCTATCTAGTGCCCATTGAAGAATATACGTGGTTCTATGGAGATGAACCGATGAAACGCGCACTCAAGCTATGTTATCTTCGGTGCGAAGGCACAACTTCTATATTGACATCCAATTTTGCTATCTGATATGGTATCTCTAATAATATTTTTATACGTAAATATCCGCCGTTAAATCTGCACCGCTATGTTTTTATAATTATATTTATTTGAAACCGAAAGCTTTTCCGTGATTAATGGTAACCGGCTAAAAAGATTACTTATAAGAAAGCTTAAGATCTTTAACATGAATGAATGCCATACATCAAGGATACTATGCCATCCTATATATGTCCACAAATATACTCTAGCATGACGGTAATATAATGGGATATATATACCCTCATAAATCATGGATACTATTTGTAAGGACTTTGGTATTTGTACAGTCATTCATGCATCAGAAACTACTAAAAAGCAAAATGAAAAGTTAATCTATGTAGATTTTGCATTGCATTCCCCTCAACCAAGCAAGCATCCAGAGGAACGAAGGACAAATGTTATTCAATTTTGTATTGCTTTCCCCTCAACCAAGCAAGCAGCCCAAAGAACAACAAATGGCATTCCCATGAGAGAAAAAAAAAATACTTGTAGGTGGGGCCACCCTCCCCTACCTATATATACCCCTGTTCACGCCGTTCCCAAGACCACACCACCAGTCCATCCATCCTGCGCTGCGCTGCGCTCGTGACAAGCATCGCAAGCAGCTCCTCCTCCTCCCCCTGTAGCCAACACCTTCCTGTTCCGGGCGCAGTAGCGGCTGTCGGCCCTCGATCTGACGAGCCCGGACCGACC

>Zm.Ra3_3_UTR

AAATGAAGACGACCTCGTGCATGCATGTAGCTAAGCTAGGCATGGCGTGGACGATGGATGGATGGACGAGCACAACAGCAAAGAGGCGCTTATTAGCTACAACTACTGGCCTTTTTTGTTGTTTTCTTTTTGCTCCTACCTATGCATAGATAGTAGTCTCTACTCTCTACTCTCTACCTAGCAACAACAACAACCTAGCTGATGCGCGCATGCATGCATGCATTGGTCGTCGCTTACTACATGCATGGCCATTCAGTGCAGTGCGTGTGTGCGATGGCGGAGAAACAACCTAGCTAGCTAGCTGCTCGATCGATCGATCGGCCTTGTCTTGGAAGAAGGCGGCAGGGAGGGGGTCCCTGTGTAACTGCTGGGGATCCGATATATCGATCTGTCCTTCCGGCCGGCAAGATCTTGTAATTAGCCGCTGTGGTACTAGCTAGCAGCTAGAGAGAAATGGATAACAAAACAAATAACATGCGTCGTCGTCTTCGTCTCCGTTC

>Zm.BA1_promoter_and_5_UTR

ATGAACAAACGATATATCACAAACCCTAGCAAGATATTATTAGTAACAGACATGTTTGTATTTGTCAATTAAAGATGCAAATGTGTTATCGAGTGTGAATAGATGATGTTTATAAACTTCCTTTACAGGAATTTCAAAAAGGAAATCCAGATAACTTGATCAAAGGTGCATTGTATGGTATAGTTGAAGACCTATGCACATTCATATTAAAAGAGGTCATTCTAGCAGAAGAAAAATATCACAACACTTCCAGTACATTAGCTATGCCAGAGTTTAGAATTTTAACAGAAGTTAGTAGACTATCTCTCAAATGAGATAGTTACTAGCTAGAGCATAGGTGAAAACTTATATGTTCAGTTTTGATGTATAGAATGTATCGAATTTAATTCAATTTTGATTGATTGAAAATTTATGGTTGTAAACAGAAAAATATATATTGTAAACAATATATTATAAACAAATTTGTGTTAATAAATATAAAATTCAGTTTTGTTTGAAAATTTGAAAAAAGGCAGGAAAACTTATACTGGCGGAAAATGGTAAGCAGACCGTCGGTGCTAATGTTATTTCCACTGGTGAGCTGCTAAAAAACCGCCTGTGAAATCATGTTTCCACACTCGGTTTCTTAAGCACACCGCTAGTGCTAATATTATTTACACTAGCGAGCTGCTAAAGAAAACCGCCAGTGATAATCATGTTTCCACATGCGGTTTCTTAAGCAAACCGTCAGTGCTAATGTTATTTCCATTGGCGGTTTCTTAAGCAGCCCGCCAGTGCAAATCATGTTTTCACATGCGGTTTCTTAAGCGAACCGCCAGTGCTAATGTTATTTCCACTAGTGGGCCGCTAAAGAAAACCGTCAGTGATAATCATGTTTCCACATGCGGTTTCTTAAGCAAACTGCCAGTGTTAATGTTATTTCCACTGGCGGGATGCTAAAGAAAACCGGCAGTGCTAATGATATTTTCACTGGCGATTGGCAAACAACCGCAAGTGAAAAAGGCCGATTTCTACTAGCCCCTAGCACTGGCGGCACTGGAAAAACGCGAGTGCAAATAGCTCTAGGACCGCCACTATAGAACTTTTGTGTACTAGTGACAGGCACGAGTAGTAACACAGTACTCAAGCAATACAACAATCAACACTACACTAAACCAGGGCGAAAGAGTGGACACGAGTATAACTCTCTGTCTCAAACCATCTGCTCGAATTCCAAACGATTCATCATTCGGAGTGAGTCTATGTGGCATCCCTTCACGAACACAAATTCTATTGTCCACTGATTATCTGAAACCATGATACTGATCTTTTTTATTTTTGGTTGTCGGAGTAATTTATGTCCTTTTTAGTCAGGTACAATCTTTCTTTCATATCAAAACCAGTTCTTTCCTTGATTCTTTCTTTTAATGCATGCCTTTTTTTGAACTTTTGAAATGATAGAATTGACTTTTCAGTGACCACTATTTAAATTTCTCCTTTTAAGCTAAGCTACTGTAAGCGGGATGGACAATAGTGATCTTCAAACCCAGTACTATATATATACGATCGCATAGCCTCATTGAAAAAAAATCTTTAATGCGTGTTAGCATTGATCTCTTTGATCAAATGGTTGTGCATTTTCTCACCAAATTTATTTTATACTTCATCTGTTCTAAATTATGTAGATATAGTGTACGTCTAAATATATAGCAAAATCAACATATGAAAATATTAGAATGGCTTATAATTTAAATCAGAGAGAATATGTCACTGCTTTTCGAAGAGCATGCAGAGTGGAAATCCCATGAATTTCACCAATTTGATACAGCGAAAATGCAATTCAGATTGTTCTCTGCATGTGCTTAACTGCTCATACACGCTTCCATGCAATGCCATACGCTGAGTATATATACCCGTCATGGGCTCCTCCCAAACCCATAGCTCTAGGGCTGAAACCAGCAAAGCCAACAGAACTGCACAGTGTAGTAGTTGCACATAGGCGTCCGCGCGTCGTCCTAGCTGGTACC

>Zm.BA1_3_UTR

AGTAGTAGTAGAAGTTTCTTAAGGTAGCATCCCGTGTGTGTTGGTGTCTGCTAGACGCTAGTACGTCTAATTAGCAAAGTTTAGCTAGTACTCGATCAATTGTCTGTCTAGTTCGCTCAGAGTTAAAGTATATGATGATGCATCTGCATATATGGGCTCTGTAATTCTGTTATCCGCTGATCGCAGATGATACACCGTATGTAATCACATGTATGTATGTTGCCTAGCTACTTCCTCTCGTGTCGATCAAGAATCTGATCTAGCTAGATGGTAAAACTATAGTGCTGCAAACTTTCTGTTTTTCTCATTAATTACCCTCTCGGTCTCAAAAGTAATTTAATTCTATAATTGAACAAAATCACCTTGTAGTATGTCGTATGAAAGACTAAAATGACTCATTTTAAATGCAGTAGTATTAAGAAGGGGGTGTATCTCCAAAGCTAGAAATTAAAACAAATAGCTTCGCAGTTGGCTTTTAAAAAGTTGGGTTGTGAGAAAAA

>ISR4

TTAGTTAGTTAGCGTCAGCCCCTCCAAGGTGGATCAAGACTGCACCGGCAAGCAGTGTAGTCTCTCTTTCTAGATTTGGCAAAGTCACTTGTCGGAGCGGTGTGATCGCACGCTTTAGCGCGGCGAGAGCGTCCTCGCGAGTTATCCCCAGGCTCGCCAAGGCCCGTGTTGCGCGTATCAAGAATCTTAGAGTTCGACTGCTGTTCACAGAGGAGCTAAGGAGATTGGACCGTGCCGCTGAACAGCCAGATCCACCGGGGGCTCCGGACCTAAGCTGCTAAAGATTTCGCAAGCGGAATCCGCCAAATCTATACAGATCCGAACCAGACAGGCGACTACGCCGTTGATCAGGGGTGAAGTTACTTACTATCGGATCTATCGTCGCAAGGAGAGACGGTTTCTGGAAACGGCCCACTCACGTCTGCTGGTCTACACGGGTCTTAAATATCGGATAGAATCGCTTATCCGCGGCTTCTAGCAAGCAGAGAGAACAACGTCTTCTTTCGCGCCCGTGCGACTTCAATAAATTGCGAGCAATTGCCCGTAGCCCAAAAATAAAAATCGATCAGGCTACCAGAACGATCAGGCAGGTACTTATATTGTAATCAAGGGAAATTTTAACGAGTTCCGACAAGGTGGAAGCCAGATTGTATCACTTAAGGCTTCTGCTTCCAACTACTTACCCTCACCACCACTTACGCTTCACCTCAAGAAGTAACTTCGTGGTTCTGTACGCCGGAGAGCTGCTCGGTAATTAACGACTAGGACCAGCGGAGCCTTAGCTTTAGAGATCACTTGAACTACACCACTTTCGACTGGGAAGTAGCAGGCAGCCTTCTCTCCGCGGGTAACGTCGAATCTGCTGATCGGCGTGCAGCTGGCCTTAAATCTGAACTCGTCCGCCTTTTTCTGTTGACCAAGAGTGGAAAAAGTGGCCCGCTCTTTTTAAATCAGCGTGACTTCGCGAAACTCCTTCGTTCTGTGAAGGGTGTGGCTTTTGCTTAGACCTAACGCTCGCCGTGGTACGCTTCGGAACACCTGCGGGGTCGATTCGATCTCCAGGTCGAGTTCAGCTCAGTAAAGGTTTATATCACCGTAAAGTCTGAGCCGTCCGACTGAGCACAAATTAACACAGTATTACGACAGGGAGTATTACAATAGATTTGCAGCGCGGGACCCTCTCAGATCAACGGTTGTACACGATAATTTAGTTAGTTAG

>Zm.Traf29_promoter_and_5_UTR

CCGAAGGTTCTCAAAGAAAATGAGCGAACTCCTAGATGTCACCGCCGTTGACGTGGTGAGTGCATTCACCTACGACACCACCAACGAGTCGTCCACGAGCTTGGACAGGGGTGACAAAAGATGGCCACCGACCTCTTCGACATAGCGGTCGCTGACGAGGAAGACGTGGTGGGGGCAATTTTCCGCAAGAGAAAAAACTCGCGTGATGCCGACGAGCCCAGCGGCGAAACGAGGGATCAACGAGAGCACCCTGGTAGACGTCAGATAAACCACTGCCCCACACGAACCGAGGAAGGAGAAGACACTGTCGCGGACCGGTCGCTAGGGGTGGGCGTTCGGGTTACCCGAAATTTTCGGGTCGGGTAATTCGGGTTTTTAAAATTTCGGGTTTTGAGAATTGATACCCGAAATTACAATGGGTTTTGCAATACCCGAAAATTCGGGTACCCGGAATTTCGGGTTCGGGTTCGGGTATTCCCGAACTACCCGAACTATTATGTTGGCTTCATAAAAACACATATACCCTATTAAATTAGTATAAAAACATAGTTTGAATAATGATATACATGGACATATAAAACATAAGCTATCTACAATCACAAGTTATGCACACTTACACATAATTATAGATGTACAAATTAATAATTAATCATGACATGAGTATATGACACATGAAAGTTCGGGTAATTCGGGTACCCGAATTTTCCGAAATAAATTCGGGTTTTGTAAGTTGCTACCCGAAATTCTCGAACAAAATTCGGGTATTTCGAGTTCAGGTTCGGGTATTCCGGATTCGGGTTTCGGGTTACGGGTTTTTTGCTCAGCCTTACCGGCCGCCCACACAACTGGCGAAGAACGACAACGACCACTTATAGAGTGGAAATACCTATAGAGTGGAAATTTTTGACACGAGGCGGTGGCTGTATGGACAGGACCGGAGGAGGATGCGCGGTACGTAGGCCGATTGTGCCAGCCTATTAGGGCATGTACAGTGGGTGTTTTAAGTTGTGTCTTACAACGTGTCTAGAGGGGTGAATGTAAAAAAACTTAAGACACGTATCTTGACGAAGACACAGTGTCTTAGCTCTATGTTCGAGACAGAAGACTAGCTGATTGGTCATTTTAATTTATTGAATGCTCTGATTGGTACAATGAATATCGTAAGACACATGTTTTAGATATGACCACTGTATTATGTTGTGTTTTAGTTGTATCTTGTACTTGGAGTACCGTGCAGCAGTATCTGGGTTGTACGTGCCCTTATGTAGGGGAAGGGAGTAGAAATACGATCAATGCGAACGCACGTTGATGACGGTGAAAACGGTAGAAACCGGTGGAAGTAGAGATTACAGCAAACATGGAGGGACGGCTTTCGTCCACCCACCGGTCCTTGGCGCGCGCGCGGCGAGCCTACGATCCAAAAGCCGCGACCGCTTGGTGTTTTGGCTGACGCTTGGGAACCTGCCGCAGCTGCATTTCTTGCTTTGGGAGCCATGTACGGCGAACTCATACTTTTGACGTCGGTTCTTTTTCGAAAAAACTTTTGACGGTCAGTTTTCCCCACTCCCGCTATATTTAAAACCCCCCACCACTCCCCCAATATTTATGACTTGACAACAATTCCCACATCGATTCATTGTCACTTGCCAGAAGTCGGGCGGCCATCGATCCCACTCCCACCGCTTACAGCCGGCCCAGTCCTCGTCGGCGCCGGACGGGGCGAAGGTAACCCCCAAGCTTCCAGATTCTCTGTCAACTTCAGTTATGCGTTTTTCTTTTAAGATGAACCTGAAACATTTTAGCTTTAGATCACCGGAGTCATTTTTTTTTTTCATTAGGCGATTGGAGTTAAAACAGGCCGAGTGGGAGAATTTCCTGGATCTTATCTGTTCTCCATCCGCACC

>Zm.Traf29_3_UTR

GCGATCGCTTAATTAAGCTCGCGATGATGGTTTTCGTTGACAGTCTTAGTATTACTACTATCTTCCTCATGCATGTAACGAGTTGCTTCGTTTCTCGTATTATGGTTATGGTTTCCCTTGGATGCATGATGAACTAGTGCTTGCAGTTGATTTTCCCCTTATTAGTTTGAGCTGCTTAATTCTTAGTAGCCCTTTTTTTTTTCTTGTGCTTGTCATGCACGTATGCACACACACATGAGACGGGCAGCTAGCATTTTCTGTGGTTCTATCTCCTCTCGGTTTTGTGCCTGTCATTTGGGGCTTAAATGTATCAGTTGTCTTTTCTCGTGCTTGGCATGCATAGTATATATATGCCCACATGAGCAAACTGCTGCTGCTTCAGTTCCAAGTAGTACAAAAGATTCAAATTAAAATTCCATCGTATTTAACCCTAGATTTTAATTGAATATATTAGATCCGCATAGTTATACCAACATGACTGATTACACATGATACGTGCCCTTTAGTTTTATATCAA

>Sevir.SPO11_promoter_and_5_UTR

TCGAATGACCTAAGGTCACTACCAATGAAAATTTCATTCTCCTTTAATATGCCAACTAGGATCTTTTTATAATGTAGTAAAGAGGTAAAGAAAAGATAGAAGGAATCATTTTCACCCAGATAGAACCACGTGTACACAATTTTCAACTCTTAATGAGTCATAAAGTTAAGTGTCGTGTAGCCTATGAAATAGAAAATATAAAATAAAGTAAGGTAGAAGTAGTTTCATCCATCTTCTATTTCTATTTTGTTAGTACGAAAGTTTCAAAAACAGTAAAAACAAAGAGCTTCGTTACCATGCTTCTAAAGTACCAGTGGTACTTGCAGGTACTTTTCTTTCTTATCCTTCTTAAAAAAAGAATTAAATAGATTTACACATAATTAAAAGGGCATCATAGTAATTTTTTACTATTTTTTACTTGGTCCCACCAAATTGATACTTCTTTTAGGTACTTCATGGCACTTCCTTTGTTTCCACCGTTCAATTTCGTTGGATGGACAGCTCTCCTTTCCTACTCTAAAATTTCTCAAAAATAAATTTTTTTTTGAAATTGGCCTAAGATGTATCCTCTCACCTATGCGCTTTTAGGGGTTCGAGTGTGGTTGGGCTGAGTGCATTGGCGTGATTATTATTTCTTAAAAAAAGTTCTATTGGCAATTCCCAGTGTGATTATTATAGGTGGACCAATGGATTTGGTGGGGTGTAGTTTGGATTGTTTATCCAGATCGAAGCATTTTATCTATTTTTAAAAGTCTTAGTCTTGATTCAGTTAAAATAATGTGCCTTTCTGTGCACACGTAAGGATGGATCCATTCCAGTACCTTCCTGTTCATTTTTATTTTGGAGGAGAGCTTTTGCAGTGATAGCTAGAGTACGTGGTATTGCGCGGAAAACAATACGTACCTTACCAATTTTACGCACGACGGATCAAACAGCTAGCACAGACTCTGCAACATATTTCTCTGTTATGAACCGTTGCAGTTAAGAATTTAAGGGCTTTACTCAACCCTTTCCCAATCGCTCCATTAGATTCCACGTAGAAGGGTTGGATGGAAGAAGTAATGGATCGACTTTTTTTTTTCACAGCCCAAACAAAAAAGAATGAAGGGTGTGAAAATAACGGATCGCCTGTTTCCGAACCAAACTCCTCCATTAAGATCTCCTCCAGTGGTTAAAGTTAAGTACTAGCTCATACACTTAAAGTTTAAAAGACATGTCTTCTGACAGTCAGAGTCAAACACTATCTCTTATAATTATATAGCACTATACGTCAACCTTATATGGATACTTATATGGATACATGTACGAGACTATCTTAAAAAAATAAAAATATGATGTGACGTGTAGCTTAGTTGGCACATCGAAAGAGGTCACATCGGATGTTTGGATGCTAATTAGAAGGACTAAACATGAGCTAATTATAAAACGAATTGCAGAACCCCTATACTAATTCGCGATACGAATCTATTAAGCCTAATTAATCCATCATTAGCAAATGGTTACTGTAGCACCACATTGTCAAATCATGGACTAATTAGGCTTAATAGATTCGTCTCACGAATTAGACTCCATCTGTGCAATTAATTTTATAATTAAATTATATTTAATACTTCTAATTAATATCAAACATCCAATGTGATAGGTACTATAAGTTTTAGGATGATGTTGCCAAACAACCTCTAAGCGAGGTGGGCGTAGCCGCGTAGCCGTTGTTGGGTCAAAAACCTGCGCCGAGTGGGAGAAGCCCGAGCCGCAGCGGCGCACGCATCCCGACCGACTCGTGTCCCGCGG

>Setit.Eef7_intron

GTTTGTGCCTCCTCGTGCTAGTAGCAATTTGACCTTTCTGGTAGTTTGATTTGTTTCTTGGTACTCGCATCTCCGATTCCGTAGGATTTTTGGGTTTTGCGTGCTAACTTGCTTCGAGGATTCGAGTCTCATGCATGGTCTCATCTAGATCCGGATAATTTCTTAGATTCTGTACGTTGATTTGTTCATTGTCCTTGTAGATCGAATGCTTAGAGGTGGCTGCTGCTTTATAGTCGATGCATATAGTAATAGTATTGTTTGATCTAGTTTCCGATCTGAACTGTAAAATTGTTTTGTTCCTGCCTTTCTAAGTGTTTATGTACTTCTGGATTTTAAATTCTGCTTAGATATTTGCTCGTTTGCTCTGTTTAGTGTTTGTCACAATTGATTCATTTATTTTCTTTTAGAGGCTGCTTCTGTGTATCCGGTTGTATAGTTTATGTTTATATAGTTGCAACATCCTTGTTTGATCTAGTTCCTGATCTGGTCTGTAAATTGTTTTGTTACTGCCTTGTAAAAGTTTTTATAATACTGGCTTCCGATTTCAGTGCTGATACTTGCTTGGTTGCTCTGTTTTGTGTTTCTAACAATTGACTCATCTGATGCAG

>Trav.Hsp16.9_3_UTR

AGTTAACAGAGCCATGGACGCAGGTCAAAGCTTGCGGCCTCGGATCTCCGGTTTCAGCTACGCGACTCGTGTGTTCTTACTCTGCTTTGGTAATTGGTATGTTTGGTGTGTACTGTTCAGTGTTTCGAAGTGTCATGTACAACTAGTCTGTACTTTGCCGGTATGTTGAGCTCGGCTCAGTATGTTCTGGGAGTGAATAAATAAAAAAAATGAACCAGATATTGTCGCGTGTACTAGTATACTAATCACCTGCTCTTGCTCTGTTTCATTCACACACACACAAAGAATCCTAAGATCACCTTGGAGGCTTTGACTGATCTTTGTGGAGGCATCTCTCGAACTGGTCCGGACTCCGGAATGATCTGTTTTCTCCCGTCCCAGAACAAGTGCACTTCTACCTCTTAAGATTCTGTTGGTTTTGAGTTTTGTTGTTTTTGTTGGAGATATTGAGGCTACTAATAAAATAAAATTACCCAATAGCTCTACTAAAGTTATCCTGCCATGTAATAATTACACCTTAGAAATAGAGGAAGTTAAAAAAGCTACTCGCAAAATAAACGTACATCTCGCAAAATAAACGCACATCTCGCTTTCCGAGAAGTCAAATT

>GUS_soybean_coding_region_1

ATGGTCCGTCCTGTAGAAACCCCAACCCGTGAAATCAAAAAACTCGACGGCCTGTGGGCATTCAGTCTGGATCGCGAAAACTGTGGAATTGATCAGCGTTGGTGGGAAAGCGCGTTACAAGAAAGCCGGGCAATTGCTGTGCCAGGCAGTTTTAACGATCAGTTCGCCGATGCAGATATTCGTAATTATGCGGGCAACGTCTGGTATCAGCGCGAAGTCTTTATACCGAAAGGTTGGGCAGGCCAGCGTATCGTGCTGCGTTTCGATGCGGTCACTCATTACGGCAAAGTGTGGGTCAATAATCAGGAAGTGATGGAGCATCAGGGCGGCTATACGCCATTTGAAGCCGATGTCACGCCGTATGTTATTGCCGGGAAAAGTGTAC

>GUS_soybean_coding_region_2

GTATCACCGTTTGTGTGAACAACGAACTGAACTGGCAGACTATCCCGCCGGGAATGGTGATTACCGACGAAAACGGCAAGAAAAAGCAGTCTTACTTCCATGATTTCTTTAACTATGCCGGAATCCATCGCAGCGTAATGCTCTACACCACGCCGAACACCTGGGTGGACGATATCACCGTGGTGACGCATGTCGCGCAAGACTGTAACCACGCGTCTGTTGACTGGCAGGTGGTGGCCAATGGTGATGTCAGCGTTGAACTGCGTGATGCGGATCAACAGGTGGTTGCAACTGGACAAGGCACTAGCGGGACTTTGCAAGTGGTGAATCCGCACCTCTGGCAACCGGGTGAAGGTTATCTCTATGAACTGTGCGTCACAGCCAAAAGCCAGACAGAGTGTGATATCTACCCGCTTCGCGTCGGCATCCGGTCAGTGGCAGTGAAGGGCGAACAGTTCCTGATTAACCACAAACCGTTCTACTTTACTGGCTTTGGTCGTCATGAAGATGCGGACTTGCGTGGCAAAGGATTCGATAACGTGCTGATGGTGCACGACCACGCATTAATGGACTGGATTGGGGCCAACTCCTACCGTACCTCGCATTACCCTTACGCTGAAGAGATGCTCGACTGGGCAGATGAACATGGCATCGTGGTGATTGATGAAACTGCTGCTGTCGGCTTTAACCTCTCTTTAGGCATTGGTTTCGAAGCGGGCAACAAGCCGAAAGAACTGTACAGCGAAGAGGCAGTCAACGGGGAAACTCAGCAAGCGCACTTACAGGCGATTAAAGAGCTGATAGCGCGTGACAAAAACCACCCAAGCGTGGTGATGTGGAGTATTGCCAACGAACCGGATACCCGTCCGCAAGGTGCACGGGAATATTTCGCGCCACTGGCGGAAGCAACGCGTAAACTCGACCCGACGCGTCCGATCACCTGCGTCAATGTAATGTTCTGCGACGCTCACACCGATACCATCAGCGATCTCTTTGATGTGCTGTGCCTGAACCGTTATTACGGATGGTATGTCCAAAGCGGCGATTTGGAAACGGCAGAGAAGGTACTGGAAAAAGAACTTCTGGCCTGGCAGGAGAAACTGCATCAGCCGATTATCATCACCGAATACGGCGTGGATACGTTAGCCGGGCTGCACTCAATGTACACCGACATGTGGAGTGAAGAGTATCAGTGTGCATGGCTGGATATGTATCACCGCGTCTTTGATCGCGTCAGCGCCGTCGTCGGTGAACAGGTATGGAATTTCGCCGATTTTGCGACCTCGCAAGGCATATTGCGCGTTGGCGGTAACAAGAAAGGGATCTTCACTCGCGACCGCAAACCGAAGTCGGCGGCTTTTCTGCTGCAAAAACGCTGGACTGGCATGAACTTCGGTGAAAAACCGCAGCAGGGAGGCAAACAATGA

>GUS_maize_coding_region_1

ATGGTGAGGCCCGTTGAGACCCCGACTAGGGAGATCAAGAAGCTGGACGGCCTCTGGGCCTTCTCCCTCGACCGTGAGAACTGCGGCATCGACCAGCGCTGGTGGGAGTCCGCCCTCCAGGAGTCTAGGGCCATCGCCGTGCCCGGTTCCTTCAACGACCAGTTCGCCGACGCCGACATCCGCAACTACGCGGGCAACGTCTGGTATCAGCGCGAGGTGTTCATCCCGAAGGGCTGGGCGGGCCAGCGCATCGTGCTCCGCTTCGACGCCGTGACCCACTACGGCAAGGTCTGGGTGAACAATCAGGAG

>GUS_maize_coding_region_2

GTGATGGAGCACCAGGGCGGTTACACCCCGTTCGAGGCCGACGTGACGCCGTACGTGATCGCCGGGAAGTCCGTCCGCATCACCGTCTGCGTGAACAATGAGCTGAACTGGCAGACCATCCCGCCTGGCATGGTCATCACCGACGAGAACGGCAAGAAGAAGCAGTCCTACTTCCACGACTTCTTCAACTACGCTGGCATCCACCGCTCCGTGATGCTCTACACCACTCCCAACACCTGGGTGGACGACATCACCGTGGTCACCCACGTGGCCCAGGACTGCAACCACGCCTCCGTGGACTGGCAAGTCGTTGCCAACGGCGACGTCAGCGTCGAGCTGCGCGACGCCGACCAGCAAGTCGTTGCCACCGGCCAGGGCACCAGCGGCACCCTCCAAGTCGTCAACCCTCACCTCTGGCAGCCTGGCGAGGGCTACCTCTACGAGCTGTGCGTCACCGCCAAGAGCCAGACTGAGTGCGACATCTACCCTCTCCGCGTCGGCATCAGGAGCGTCGCTGTCAAGGGCGAGCAGTTCCTCATCAACCACAAGCCTTTCTACTTCACTGGTTTCGGCCGCCACGAGGACGCTGACCTGAGGGGCAAGGGTTTCGACAACGTCCTGATGGTCCACGACCACGCTCTGATGGACTGGATCGGTGCCAACAGCTACAGGACCAGTCACTACCCGTACGCTGAGGAGATGCTGGACTGGGCTGACGAGCACGGTATCGTCGTGATCGACGAGACTGCTGCGGTCGGTTTCAACCTGTCTCTGGGCATTGGTTTCGAGGCTGGGAACAAGCCGAAGGAGCTGTACTCTGAGGAAGCTGTCAACGGCGAGACTCAGCAAGCTCATCTCCAGGCGATTAAGGAGCTGATTGCCAGGGACAAGAACCATCCGTCTGTCGTGATGTGGTCTATTGCGAATGAGCCGGACACCAGACCGCAAGGGGCGCGTGAATACTTCGCGCCGCTGGCGGAGGCGACTCGCAAACTGGACCCAACCCGTCCAATCACGTGCGTCAATGTCATGTTCTGCGACGCCCATACGGATACGATCTCGGACCTGTTCGATGTTCTTTGTCTCAATCGGTACTATGGGTGGTATGTTCAGAGCGGGGATCTTGAGACGGCGGAGAAGGTTCTTGAGAAGGAACTCCTGGCGTGGCAAGAGAAGCTCCATCAGCCGATCATTATCACGGAGTACGGGGTTGACACACTTGCGGGCCTTCACAGTATGTACACAGATATGTGGTCGGAGGAATACCAGTGTGCATGGTTGGATATGTACCATCGTGTCTTCGACCGGGTTTCAGCGGTTGTCGGCGAACAAGTCTGGAACTTCGCAGACTTCGCCACGAGCCAAGGGATACTGCGGGTAGGAGGGAACAAGAAGGGAATCTTCACACGGGATCGGAAGCCCAAGTCAGCAGCCTTCCTGTTGCAGAAGCGATGGACAGGAATGAACTTCGGAGAAAAGCCACAGCAAGGCGGAAAGCAGTGA

**Table S6** Positive staining summary table from GUS reporter genes. For root staining, a positive value for root whole mount (WM) refers to broad staining throughout the root, whereas a positive value for a part of the root, such as “primary tip”, refers to staining status in that specific part of the root.

Promoter: Glyma.CALa


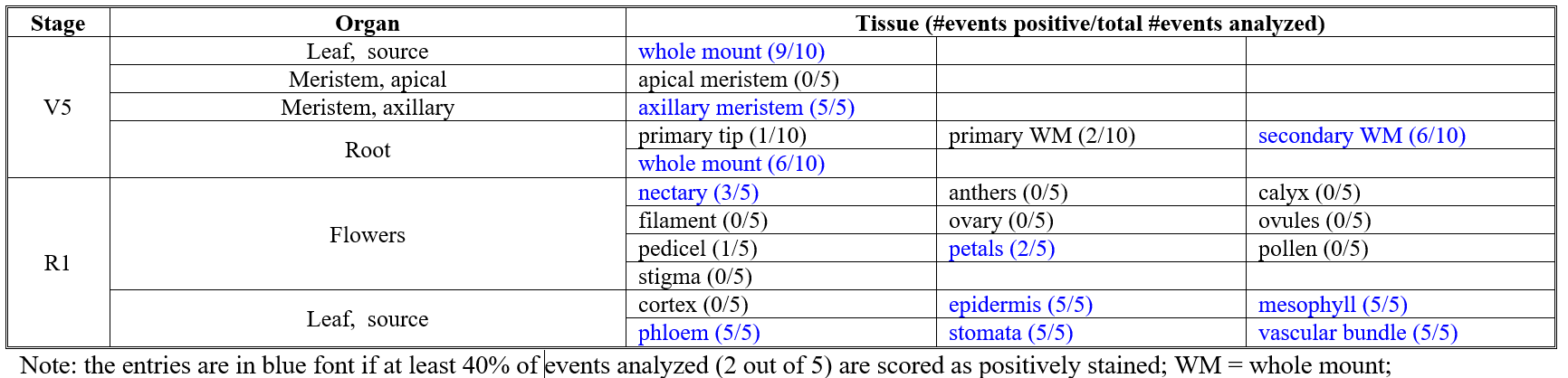


Promoter: Glyma.Mads17


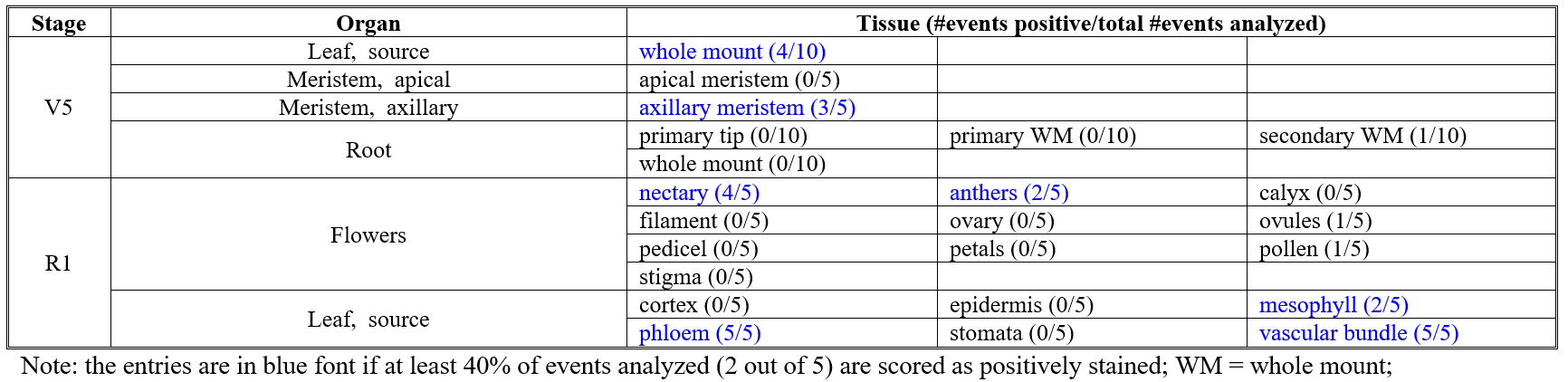


Promoter: Glyma.AP1


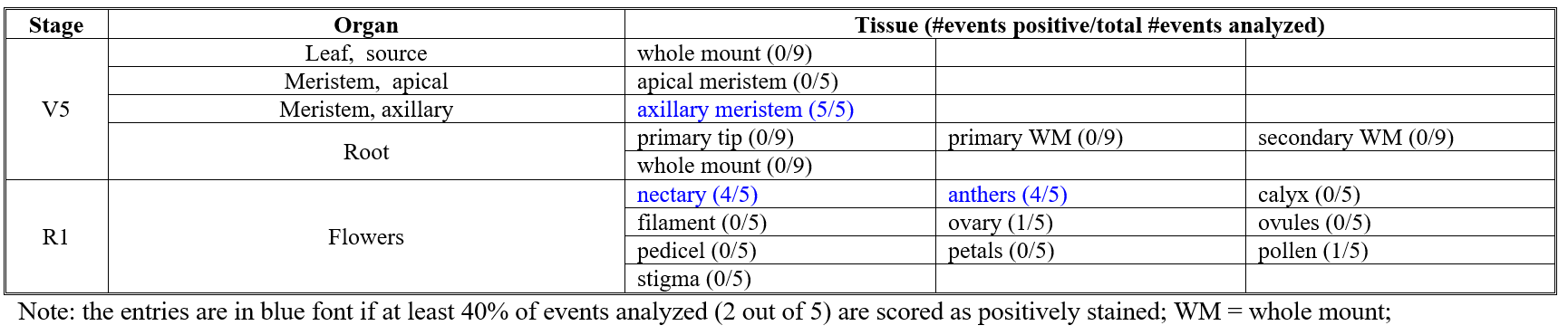


Promoter: Zm.Traf29


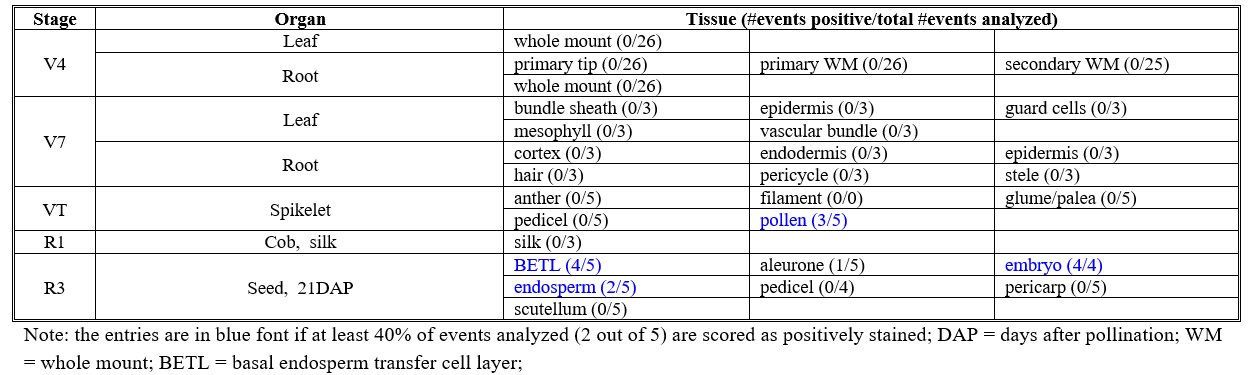


Promoter: Sevir.SPO11


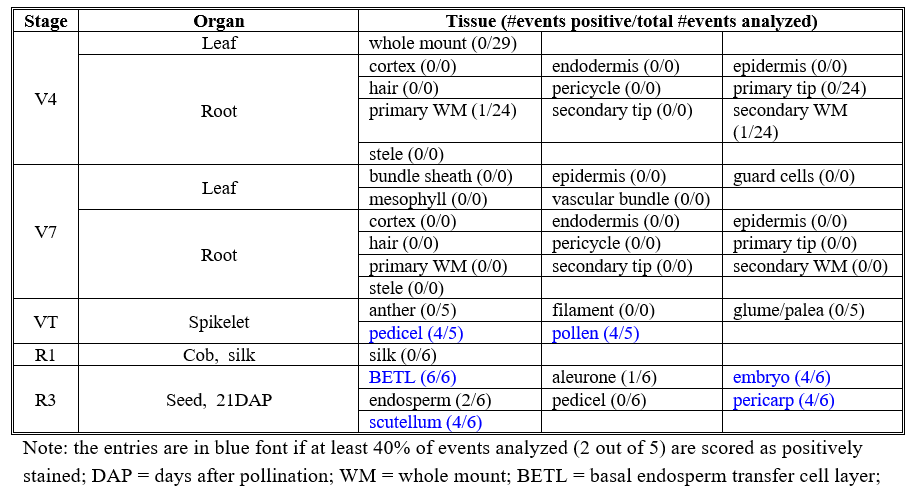


**References**

1. Wang X, Elling AA, Li X, Li N, Peng Z, He G, et al. Genome-wide and organ-specific landscapes of epigenetic modifications and their relationships to mRNA and small RNA transcriptomes in maize. Plant Cell 2009;21:1053-69.
2. Jia Y, Lisch DR, Ohtsu K, Scanlon MJ, Nettleton D, Schnable PS. Loss of RNA-dependent RNA polymerase 2 (RDR2) function causes widespread and unexpected changes in the expression of transposons, genes, and 24-nt small RNAs. PLoS Genet. 2009;5:e1000737.
3. Schnable PS, Ware D, Fulton RS, Stein JC, Wei F, Pasternak S, et al. The B73 maize genome: complexity, diversity, and dynamics. Science 2009;326:1112-5.
4. Bolduc N, Yilmaz A, Mejia-Guerra MK, Morohashi K, O'Connor D, Grotewold E, et al. Unraveling the KNOTTED1 regulatory network in maize meristems. Genes Dev. 2012;26:1685-90.
5. Davidson RM, Hansey CN, Gowda M, Childs KL, Lin H, Vaillancourt B, et al. Utility of RNA Sequencing for Analysis of Maize Reproductive Transcriptomes. Plant Genome 2011; 4.
6. Li P, Ponnala L, Gandotra N, Wang L, Si Y, Tausta L, et al. The developmental dynamics of the maize leaf transcriptome. Nat Genet. 2010;42:1060-67.
7. Kakumanu A, Ambavaram MM, Klumas C, Krishnan A, Batlang U, Myers E, et al. Effects of drought on gene expression in maize reproductive and leaf meristem tissue revealed by RNA-Seq. Plant Physiol. 2012;160:846-67.
8. Paschold A, Jia Y, Marcon C, Lund S, Larson NB, Yeh C-T, et al. Complementation contributes to transcriptome complexity in maize (Zea mays L.) hybrids relative to their inbred parents. Genome Res. 2012;22:2445-54.
9. Singh M, Goel S, Meeley RB, Dantec C, Parrinello H, Michaud C, et al. Production of viable gametes without meiosis in maize deficient for an ARGONAUTE protein. Plant Cell 2011;23:443-58.
10. Zhan J, Thakare D, Ma C, Lloyd A, Nixon NM, Arakaki AM, et al. RNA sequencing of laser-capture microdissected compartments of the maize kernel identifies regulatory modules associated with endosperm cell differentiation. Plant Cell 2015;27:513-31.
11. Yu P, Baldauf JA, Lithio A, Marcon C, Nettleton D, Li C, et al. Root Type-Specific Reprogramming of Maize Pericycle Transcriptomes by Local High Nitrate Results in Disparate Lateral Root Branching Patterns. Plant Physiol. 2016;170:1783-98.
12. Chen J, Strieder N, Krohn NG, Cyprys P, Sprunk S, Engelmann JC, Dresselhaus T. Zygotic genome activation occurs shortly after fertilization in maize. Plant Cell 2017;29:2106-25.
13. Chen J, Zeng B, Zhang M, Xie S, Wang G, Hauck A, et al. Dynamic transcriptome landscape of maize embryo and endosperm development. Plant Physiol. 2014;166:252-64.
14. Shen Y, Zhou Z, Wang Z, Li W, Fang C, Wu M, et al. Global dissection of alternative splicing in paleopolyploid soybean. Plant Cell. 2014;26:996-1008.
